# The histone modification reader ZCWPW1 promotes double-strand break repair by regulating cross-talk of histone modifications and chromatin accessibility at meiotic hotspots

**DOI:** 10.1186/s13059-022-02758-z

**Published:** 2022-09-06

**Authors:** Shenli Yuan, Tao Huang, Ziyou Bao, Shiyu Wang, Xinyue Wu, Jiang Liu, Hongbin Liu, Zi-Jiang Chen

**Affiliations:** 1 Center for Reproductive Medicine, Shandong University, Jinan, 250012 Shandong China; 2 CAS Key Laboratory of Genome Sciences and Information, Collaborative Innovation Center of Genetics and Development, Beijing Institute of Genomics, China National Center for Bioinformation, and Chinese Academy of Sciences, Beijing, China; 3 University of Chinese Academy of Sciences, Beijing, China; 4 Key Laboratory of Reproductive Endocrinology of Ministry of Education, Shandong University, Jinan, 250012 Shandong China; 5 Shandong Provincial Clinical Medicine Research Center for Reproductive Health, Shandong University, Jinan, 250012 Shandong China; 6 National Research Center for Assisted Reproductive Technology and Reproductive Genetics, Shandong University, Jinan, China; 7 CAS Center for Excellence in Animal Evolution and Genetics, Chinese Academy of Sciences, Kunming, China; 8 CUHK-SDU Joint Laboratory on Reproductive Genetics, School of Biomedical Sciences, Chinese University of Hong Kong, Hong Kong, China; 9 Shanghai Key Laboratory for Assisted Reproduction and Reproductive Genetics, Shanghai, 200135 China; 10 Center for Reproductive Medicine, Ren Ji Hospital, School of Medicine, Shanghai Jiao Tong University, Shanghai, 200135 China

## Abstract

**Background:**

The PRDM9-dependent histone methylation H3K4me3 and H3K36me3 function in assuring accurate homologous recombination at recombination hotspots in mammals. Beyond histone methylation, H3 lysine 9 acetylation (H3K9ac) is also greatly enriched at recombination hotspots. Previous work has indicated the potential cross-talk between H3K4me3 and H3K9ac at recombination hotspots, but it is still unknown what molecular mechanisms mediate the cross-talk between the two histone modifications at hotspots or how the cross-talk regulates homologous recombination in meiosis.

**Results:**

Here, we find that the histone methylation reader ZCWPW1 is essential for maintaining H3K9ac by antagonizing HDAC proteins’ deacetylation activity and further promotes chromatin openness at recombination hotspots thus preparing the way for homologous recombination during meiotic double-strand break repair. Interestingly, ectopic expression of the germ-cell-specific protein ZCWPW1 in human somatic cells enhances double-strand break repair via homologous recombination.

**Conclusions:**

Taken together, our findings provide new insights into how histone modifications and their associated regulatory proteins collectively regulate meiotic homologous recombination.

**Supplementary Information:**

The online version contains supplementary material available at 10.1186/s13059-022-02758-z.

## Background

Meiotic homologous recombination during the first meiotic prophase, a process that increases genome diversity in gametes, and thus, is a hallmark not only for sexual reproduction, but also evolution [1–4]. Remarkably, this reaction is initiated by the formation of programmed DNA double-strand breaks (DSBs), which are preferentially formed in permissive regions known as recombination hotspots [5, 6]. Meiotic DSBs are catalyzed by an evolutionarily conserved topoisomerase-like protein complex consisting of SPO11 dimers and their accessory factors [7, 8]. At a DSB, SPO11 becomes covalently bound to each 5′ end of the broken DNA strand, and then, it is endonucleolytially released from the broken ends and one strand is resected in the 5′-3′ direction by the MRE11-RAD50-NBS1 exonuclease complex to expose 3′-overhangs [9–14]. Exposed single-strand DNA (ssDNA) tails are coated by the heterotrimeric ssDNA-binding protein RPA, and then BRCA2 removes the RPA from ssDNA and recruits the RecA recombinase homologs RAD51 and DMC1 [15–17]. RAD51 and DMC1 nucleoprotein filaments subsequently search for and invade homologous duplex DNA to form recombination intermediates [18–22]. Following invasion of the homologous strand, recombination intermediates can be resolved via various DSB repair pathways to produce crossovers or non-crossovers [4, 23, 24].

Meiotic DSBs and crossovers are distributed non-randomly on chromosomes and are regarded as recombination hotspots [25–30], which display multiple levels of temporal and spatial organization. In yeast, most hotspots are positioned within nucleosome-depleted regions at intergenic regions containing promoters, and H3K4me3 is a hotspot-associated histone modification [29, 31–33]. In mice, recombination hotspots are also enriched at a subset of H3K4me3 sites, but unlike budding yeast, the overlap of these hotspots and H3K4me3 does not generally occur near promoter regions [30, 34]. Instead, mammalian DSB hotspots are determined by the meiosis-specific methyltransferase PRDM9, an enzyme with a PR/SET domain in its central region that has both H3K4 and H3K36 methyltransferase activity. The C2H2 zinc fingers at the PRDM9 C-terminal region have DNA-binding activity that enables the specification of recombination hotspots [35–39]. In yeast, the COMPASS subunit Spp1 promotes DSB formation at promoters by tethering H3K4me3 sites to chromosome axes with the DSB formation machinery [40, 41]. In mice, the H3K4me3 and H3K36me3 marks at hotspots catalyzed by PRDM9 appear in leptonema, are maximal in zygonema, and are removed at pachynema [42]. Multiple studies have demonstrated that PRDM9 and its methyltransferase activity determine the locations of DSB formation [39, 43–45].

Beyond the known enrichment for H3K4me3 and H3K36me3 at recombination hotspots, H3 lysine 9 acetylation (H3K9ac) is also enriched at recombination hotspots [34, 42]. It is conspicuous that both H3K4me3 and H3K9ac are established concurrently at the leptotene and zygotene stage and that both are removed at pachytene stage [34, 42]. A study of natural variants of the histone methylation writer PRDM9 reported differential accumulation of H3K9ac marks at meiotic hotspots [46]. However, to date no studies have shown any direct biomolecular links between these epigenetic regulatory marks. Recently, three independent groups found that the histone methylation reader ZCWPW1 could specifically recognize dual histone methylation marks (H3K4me3 and H3K36me3) deposited by PRDM9 at recombination hotspots in mice [47–50]. Much less is known about how ZCWPW1 reads these epigenetic marks and thus participates in the meiotic recombination process to promote DSB repair [51].

In addition to histone modifications, chromatin accessibility is believed to be essential for homologous recombination [52–55]. In budding yeast, meiotic recombination occurs preferentially at specific sites (hotspots) that often reside in open regions at gene promoters [29]. In mice, the chromatin remodeler HELLS, which is recruited to the recombination hotspots by PRDM9, can open chromatin at future DSB hotspots and is required for DSB activity at PRDM9 sites [46, 56]. It is well-established that some active histone modifications such as H3K4me3 and H3K9ac regulate gene expression by recruiting chromatin remodeler proteins, which ultimately makes the DNA in chromatin more accessible to transcription factors [57, 58]. However, for meiotic homologous recombination in mammals, it is still unclear whether and how histone modifications affect chromatin accessibility at recombination hotspots.

In this study, we found that the germ-cell-specific protein ZCWPW1, the first histone methylation reader at meiotic recombination hotspots in mammals, functions to prevent histone deacetylases from removing H3K9ac, thus promoting chromatin openness at recombination hotspots. Interestingly, ectopic expression of ZCWPW1 in human somatic cells is able to promote DSB repair via homologous recombination.

## Results

### The histone methylation reader ZCWPW1 binds at hotspots prior to DSB formation

Three research groups independently reported that the histone modification reader ZCWPW1 can specifically recognize the dual histone modification marks H3K4me3 and H3K36me3 catalyzed by PRDM9 at recombination hotspots [47, 49, 50]. ZCWPW1 and PRDM9 are co-expressed in meiosis prophase I spermatocytes [49], and knockout of *Zcwpw1* in male mice results in the complete failure of meiosis prophase I, which leads to spermatocytes arrested at the zygotene stage (with incomplete DSB repair) [48]. However, it was notable that both the number and position of DSBs were induced normally in the *Zcwpw1*^*−/−*^ mouse testes [49, 50]. These results indicate that ZCWPW1 is dispensable for the formation and location of DSBs but is required for proper DSB repair during the later steps of homologous recombination.

Our initial inquiry in the present study examined whether DSBs are required for ZCWPW1 occupancy at recombination hotspots. We performed ChIP-seq for ZCWPW1 in testes collected from postnatal day (PD) 14 mice deficient in *Spo11*, a protein known to initiate meiotic recombination by generating chromosome breaks (DSBs) [8]. We found that the ZCWPW1 signal was quite similar between wild type (WT) and *Spo11*^*−/−*^ testes for virtually all of the detected ZCWPW1 binding sites (Fig. S1a and S1b), indicating that ZCWPW1 occupancy at recombination hotspots is independent of SPO11 in testes and therefore must precede meiotic DSB formation.

### ZCWPW1 is required for H3K9ac at meiotic recombination hotspots

**Fig. 1:**
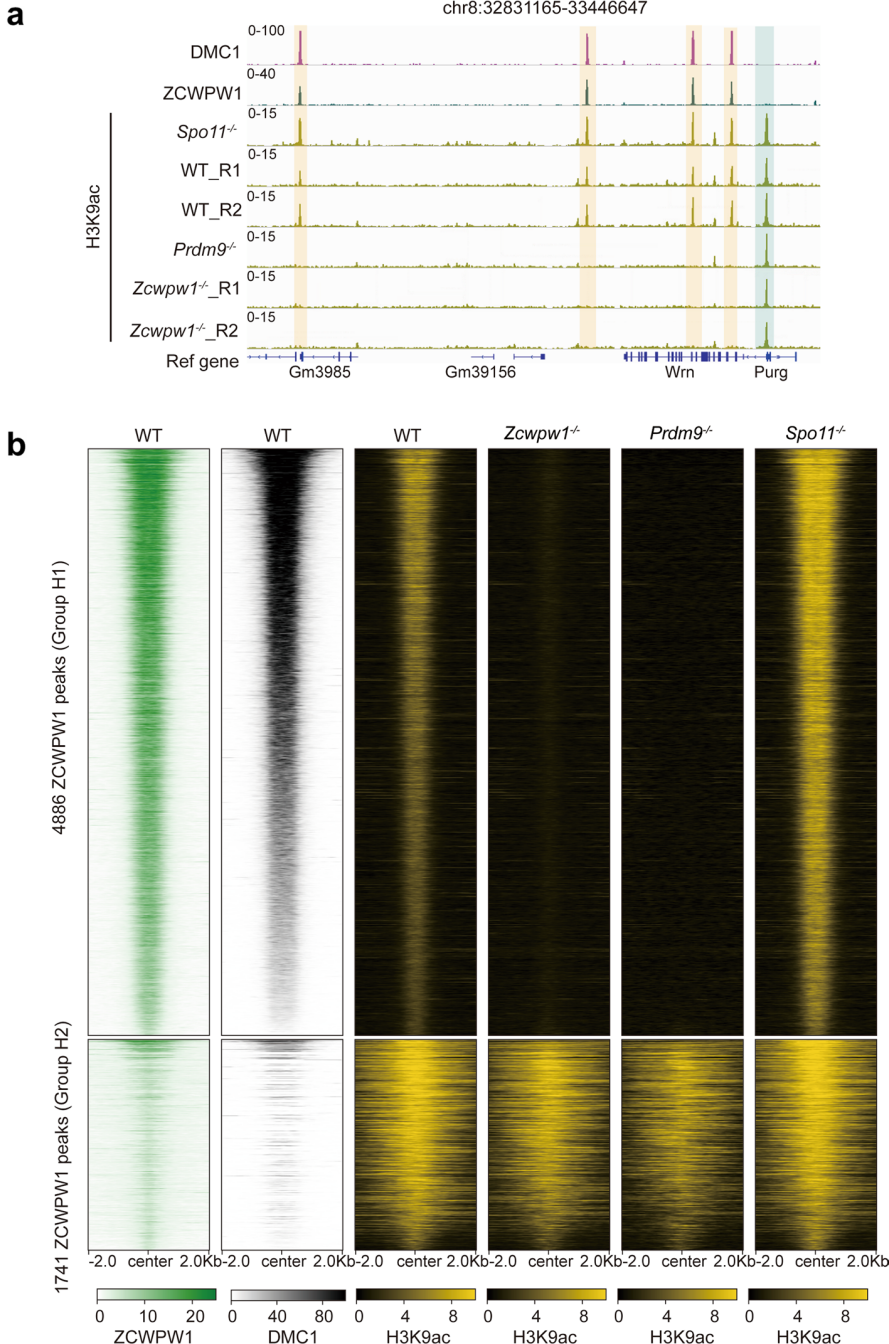
ZCWPW1 is required for H3K9ac modification of recombination hotspots during male meiosis. **a** The genome browser view of DMC1 and ZCWPW1 signals in WT mouse testes and the H3K9ac signal in WT, *Spo11*^*–/–*^, *Prdm9*^*–/–*^, and *Zcwpw1*^*–/–*^ testes. H3K9ac peaks overlapping hotspot sites are indicated by orange shaded areas, while H3K9ac peaks at promoter regions are indicated by blue shaded areas. **b** Heatmap showing the ZCWPW1 and DMC1 signals in WT testes and the H3K9ac signal in WT, *Spo11*^*–/–*^, *Prdm9*^*–/–*^, and *Zcwpw1*^*–/–*^ testes at two distinct groups of ZCWPW1 binding sites (peaks). Group H1 represents those ZCWPW1 binding sites that lost the H3K9ac signal in *Zcwpw1*^*–/–*^ testes, and group H2 represents those ZCWPW1 binding sites that retained the H3K9ac signal in *Zcwpw1*^*–/–*^ testes. The regions of group H1 or H2 in heatmaps were ordered from largest to smallest based on the average signal in all samples

A previous study reported that loss of H3K4me3 at hotspots (due to mutation of the H3K4me3 writer PRDM9) in C57BL/6 mouse strains was accompanied by the loss of H3K9ac [46], indicating that PRDM9-deposited H3K4me3 may be a pre-requisite for H3K9ac modification at meiotic hotspots. However, the molecular mechanism for such cross-talk between histone modifications is poorly understood. Here, we hypothesized that the histone methylation reader ZCWPW1 mediates the cross-talk between H3K4me3 and H3K9ac. To test this, we analyzed the H3K9ac signal on ZCWPW1 binding sites in a published H3K9ac ChIP-seq dataset that was generated from isolated, stage-specific spermatocyte nuclei [42, 47]. We found that 94.5% (13807/14688) of the ZCWPW1 binding sites were enriched with H3K9ac (Additional file 1: Fig. S2a). In our H3K9ac ChIP-seq data from whole testes, we found that 45% (6627/14688) of the ZCWPW1 binding sites were enriched with H3K9ac (Fig. 1a, b and Additional file 1: S2c). The higher level of H3K9ac enrichment in the sorted meiotic cells makes sense, given that ZCWPW1 is specifically expressed in germ cells, thus allowing the elimination of H3K9ac originating from cells that did not express ZCWPW1.

We subsequently compared the H3K9ac signal between WT and *Zcwpw1*^*−/−*^ mouse testes. We first found that the H3K9ac signal at all promoter regions was comparable between WT and *Zcwpw1*^*−/−*^ mouse testes (Additional file 1: Fig. S2b). We have detected 6627 H3K9ac peaks overlapping ZCWPW1 peaks in whole testes (Fig. 1b and Additional file 1: S2c). Interestingly, 74% (4886/6627) (group H1) of the ZCWPW1 binding sites lost the H3K9ac signal in *Zcwpw1*^*−/−*^ mouse testes (Fig. 1a, b group H1 and Additional file 1: S2c), suggesting that ZCWPW1 functionally impacts the H3K9ac at ZCWPW1 binding sites through some unknown mechanism. However, 26% (1741/6627) (group H2) of the ZCWPW1 binding sites retained the H3K9ac signal in *Zcwpw1*^*−/−*^ mouse testes (Fig. 1b group H2 and Additional file 1: S2c). We also found that 73.4% of the group H2 ZCWPW1 binding sites are located in transcript promoter regions, while only 4.1% of the group H1 ZCWPW1 binding sites are located in promoter regions (Additional file 1: Fig. S2d). The ZCWPW1 signal at group H1 ZCWPW1 binding sites was greatly stronger than the group H2 sites (Fig. 1b and Additional file 1: S2e). We also found that the DMC1 signal at group H1 ZCWPW1 binding sites was greatly stronger than the group H2 sites (Fig. 1b and Additional file 1: S2e). These results suggested that the presence of H3K9ac at recombination hotspots is modulated by ZCWPW1.

We also found that no H3K9ac signal was evident at group H1 ZCWPW1 binding sites in *Prdm9*^*−/−*^ mouse testes (Fig. 1a, b). This loss-of-Prdm9-function was consistent with a recent study showing that germ cells expressing natural variants of the histone methylation writer PRDM9 (Prdm9Cst and Prdm9Dom) display differential accumulation of H3K9ac marks at meiotic hotspots [46]. However, we found that the H3K9ac signal was evident at group H1 ZCWPW1 binding sites in *Spo11*^*−/−*^ mouse testes (Fig. 1a, b and Additional file 1: S2e), indicating that the presence of H3K9ac was independent of DSBs, which was consistent with previous reports [34, 46]. Taken together, these results suggest that H3K9ac modification at recombination hotspots is dependent on both the histone methylation writer PRDM9 and the histone methylation reader ZCWPW1.

### ZCWPW1 physically interacts with multiple HDAC proteins and with known chromatin architecture remodeling proteins

**Fig. 2:**
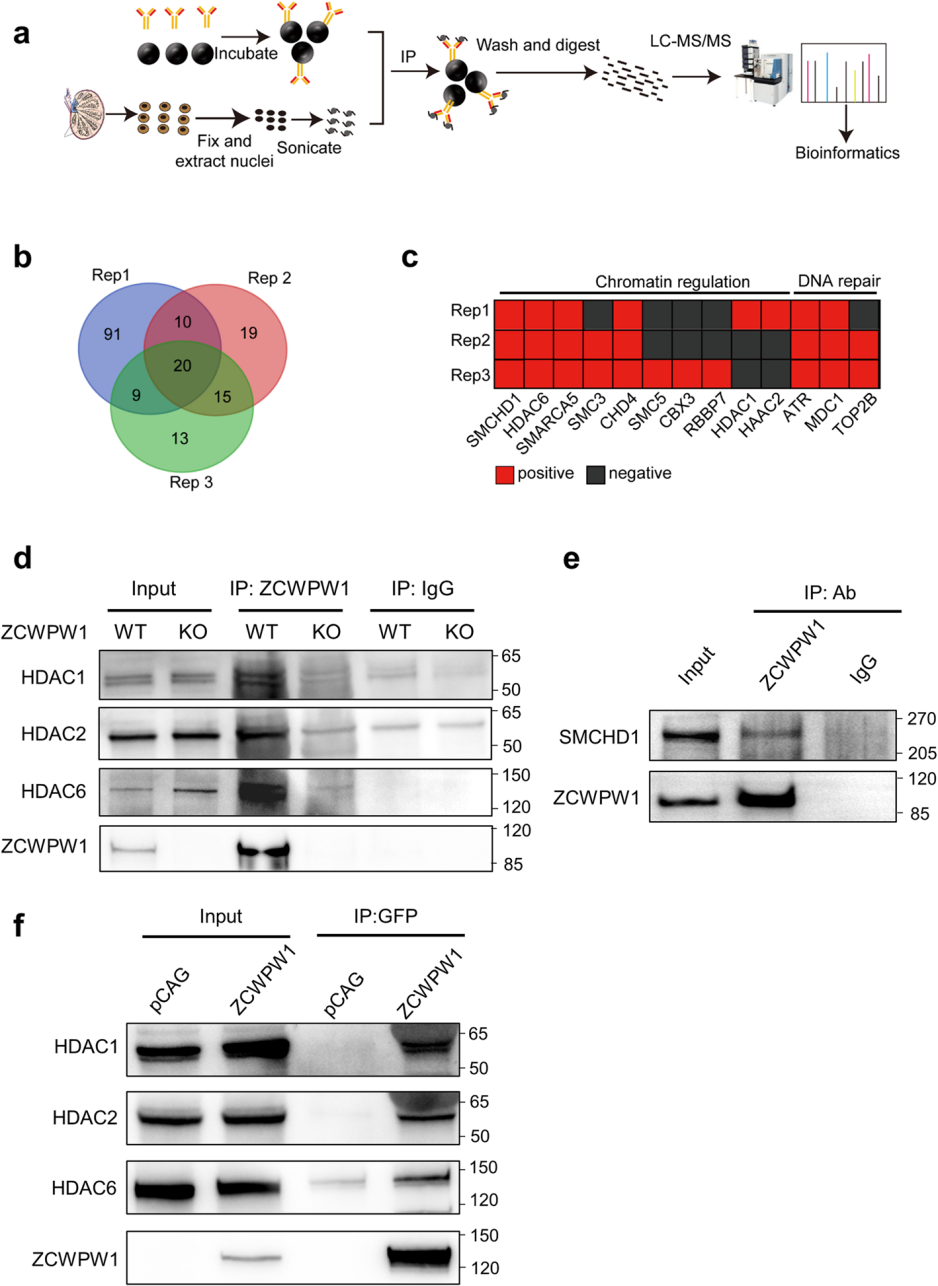
ZCWPW1 physically interacts with multiple HDAC proteins. **a** Schematic diagram illustrating the experimental design for the RIME analysis. **b** Venn diagram showing the overlap of proteins binding to ZCWPW1 among the three repeats (Rep1, Rep2, and Rep3) in the RIME analysis. **c** Example of putative ZCWPW1-interacting proteins. The proteins are associated with chromatin regulation and DNA repair. **d** Co-IP analysis of ZCWPW1 binding proteins from PD14–PD16 testes protein extracts in WT and *Zcwpw1*^*–/–*^ mice. HDAC1, HDAC2, and HDAC6 were immunoprecipitated with ZCWPW1. Data are representative of three independent experiments. **e** Co-IP analysis of ZCWPW1 binding proteins from PD14–PD16 testes protein extracts in WT mice. SMCHD1 was immunoprecipitated with ZCWPW1. Data are representative of three independent experiments. **f** Co-IP analysis of the interaction between ZCWPW1 and HDAC1, HDAC2, and HDAC6 in HeLa cells overexpressing ZCWPW1 for 48 h. Data are representative of three independent experiments

To explore how ZCWPW1 affects H3K9ac at recombination hotspots, we used the rapid immunoprecipitation mass spectrometry of endogenous proteins (RIME) approach to identify proteins that interact with ZCWPW1 in testes (Fig. 2a) [59]. About 177 proteins that putatively interact with ZCWPW1 were detected in at least one of the three samples in our RIME assay (Fig. 2b and Additional file 2: Table S1). Interestingly, several of these putative ZCWPW1-interacting proteins have functional annotations related to chromatin regulation and/or DSB repair (Fig. 2c). Unexpectedly, we found three HDACs among the chromatin-regulating proteins, including HDAC6, HDAC1, and HDAC2 (Fig. 2c).

We subsequently performed Co-immunoprecipitation (Co-IP) assays to confirm the HDAC-ZCWPW1 interactions in testes. The three examined HDACs (HDAC6, HDAC1, and HDAC2) were pulled down using a ZCWPW1 antibody (Fig. 2d), and ZCWPW1 was pulled down using an HDAC antibody (Additional file 1: Fig. S3a, S3b, and S3c). The three examined HDACs (HDAC6, HDAC1, and HDAC2) were also pulled down using a GFP antibody in HeLa cells transfected with ZCWPW1-GFP plasmid (Fig. 2f). In addition, we also used yeast two hybrid assays and found that the HDACs interact with ZCWPW1 (Additional file 1: Fig. S3e and S3f). Apart from HDACs, we also identified several known chromatin-remodeling proteins in our RIME assay, including SMCHD1 [60], SMARCA5 [61], and CHD4 [62]. We chose SMCHD1 and confirmed its interaction with ZCWPW1 using a Co-IP assay (Fig. 2e and Additional file 1: Fig. S3d) and yeast two hybrid assays (Fig. S3e and S3f). Taken together, these results suggest that ZCWPW1 may function in chromatin regulation through its interactions with HDACs and other chromatin-remodeling proteins.

### ZCWPW1 preserves H3K9ac by antagonizing HDACs’ deacetylation activity

**Fig. 3:**
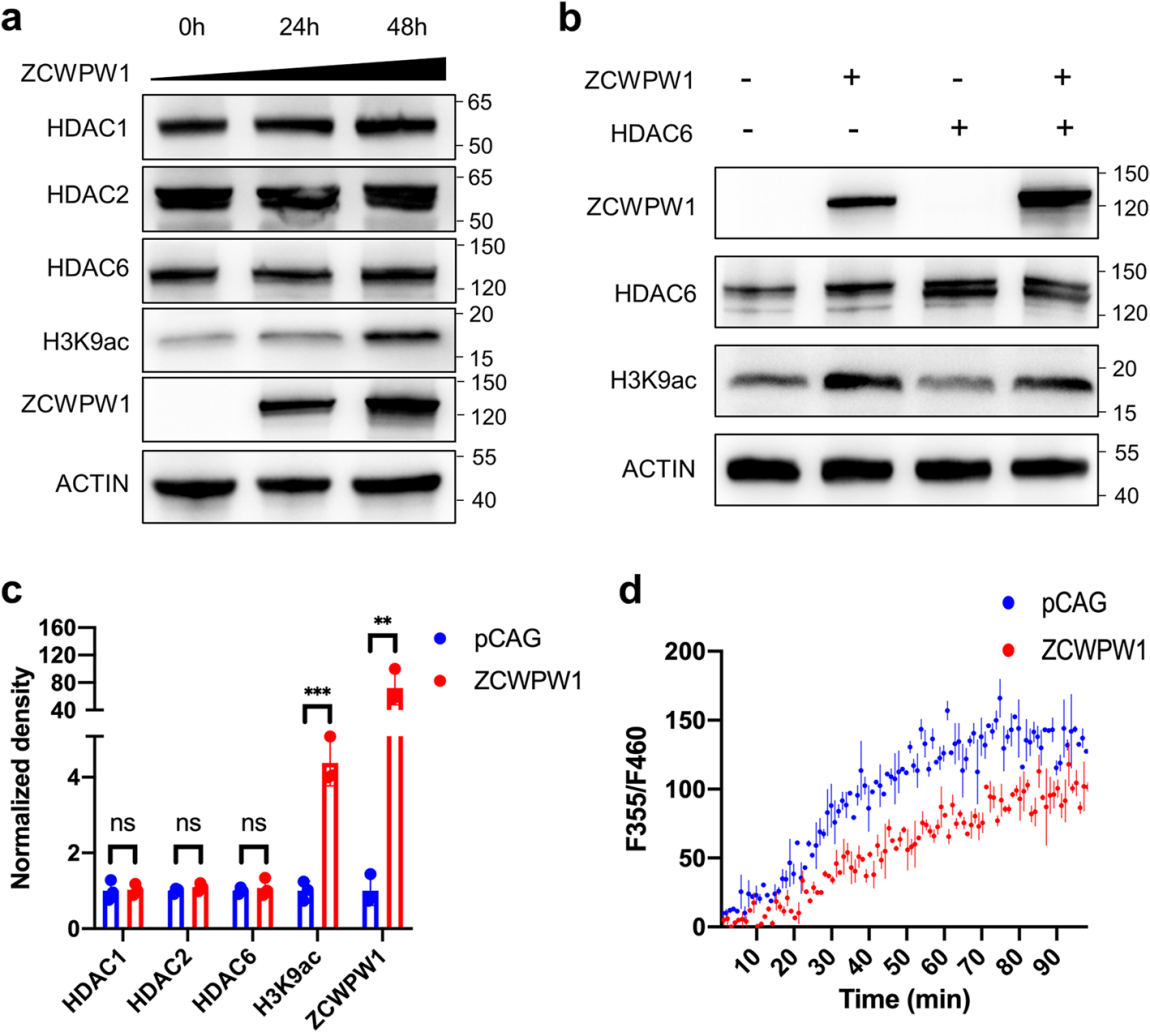
ZCWPW1 preserves H3K9ac by antagonizing HDAC’s deacetylation activity. **a** Immunoblotting of HDAC1, HDAC2, HDAC6, H3K9ac, and ZCWPW1 in HeLa cells transfected with the empty pCAG vector or with ZCWPW1-EGFP-pCAG at the indicated time points. **b** Immunoblot analysis of H3K9ac levels after overexpression of HDAC6 alone or co-expression of HDAC6 and ZCWPW1 in HeLa cells for 48 h. **c** Quantification of HDAC1, HDAC2, HDAC6, H3K9ac, and ZCWPW1 levels in HeLa cells transfected with empty pCAG vector or with ZCWPW1-EGFP-pCAG for 48 h. **P < 0.01, ***P < 0.001 by Student’s *t* test. Data represent the mean ± SEM from three independent experiments. **d** Inhibition of HDAC activity by ZCWPW1. HeLa cells were transfected with pCAG or ZCWPW1-EGFP-pCAG for 36 h, and the nuclear extracts were assessed with histone deacetylase activity assay kits according to the manufacturer’s instructions. Data are representative of three independent experiments

After finding that ZCWPW1 could interact with several HDACs in mouse testes and cell lines (Fig. 2d, f), we investigated the impact of loss-of-Zcwpw1-function on HDACs and acetyl lysine in mouse testes. We first performed immunoblotting analysis of ZCWPW1-interacting HDACs, acetyl lysine, and several histone H3 acetylation marks (H3K9ac, H3K27ac, H3K56ac) in PD14 WT and *Zcwpw1*^*–/–*^ testes. However, the levels of the candidate proteins were comparable between WT and *Zcwpw1*^*–/–*^ testes (Additional file 1: Fig. S4a, S4b and S4c). Further, we expressed ZCWPW1 in HeLa cells, and no obvious differences in the levels of HDAC1/2/6 were detected between the two groups (Fig. 3a–c). These results suggested that the interaction between ZCWPW1 and HDACs had no obvious effect on the protein level of HDACs in mouse testes or in HeLa cells.

Given that knock-out of *Zcwpw1* could greatly reduce the H3K9ac signal at recombination hotspots (Fig. 1b) but did not affect the protein levels of the HDACs that ZCWPW1 interacts with (Additional file 1: Fig. S4a), we hypothesized that ZCWPW1 antagonizes the deacetylation activity of HDACs to maintain the H3K9ac signal. To test this, we expressed ZCWPW1 in HeLa cells. The H3K9ac signal was significantly increased in HeLa cells expressing ZCWPW1 compared to control cells transfected with an empty vector (Fig. 3a–c). Further, we expressed HDAC6 alone or with ZCWPW1 in HeLa cells, and the H3K9ac signal was slightly reduced in the HDAC6 overexpression group but was partly recovered in the ZCWPW1 and HDAC6 co-expression group (Fig. 3b). We also performed HDAC activity assays in HeLa cells, and as expected, the deacetylation activities of HDACs were modestly decreased in cells expressing ZCWPW1 compared to controls (Fig. 3d). Thus, ZCWPW1’s interaction with HDAC enzymes restricts their deacetylation activities, at least partially explaining the elevated H3K9ac levels at recombination hotspots.

It has been reported that ZCWPW1 is enriched at hotspot sites and at numerous non-hotspot sites, including promoter regions (which are enriched with either H3K4me3 or H3K36me3) in HEK293T cells co-overexpressing PRDM9 and ZCWPW1 [50]. When we expressed ZCWPW1 in HeLa cells, we found that the H3K9ac signal overlapping the ZCWPW1 binding sites was significantly greater than that in HeLa cells expressing control plasmids (Additional file 1: Fig. S4d and S4e). Given that ZCWPW1 only recognizes dual histone methylation marks deposited by PRDM9 at hotspots in testes [47, 49], and given that we found that 74% (4886/6627) (group H1) of the ZCWPW1 binding sites lost their H3K9ac signal in *Zcwpw1*^*–/–*^ testes (Fig. 1a, b) while the H3K9ac signal at all promoter regions was comparable in WT and *Zcwpw1*^*–/–*^ testes (Additional file 1: Fig. S2b), we suggest that the function of ZCWPW1 in preserving H3K9ac by antagonizing HDAC protein deacetylation activity is partially dependent on its ability to recognize H3K4me3 and/or H3K36me3.

### ZCWPW1 functions to support meiotic DSB repair by maintaining chromatin in an open state

**Fig. 4:**
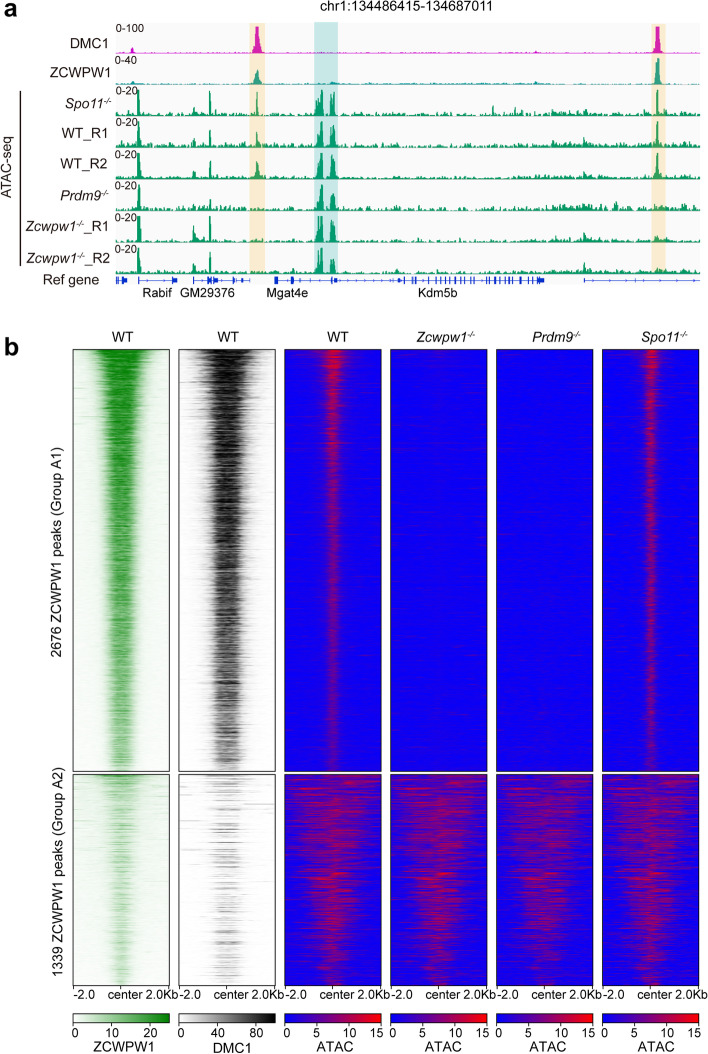
ZCWPW1 functions in opening chromatin to support DSB repair during male meiosis. **a** Genome browser view of the binding signals for DMC1 and ZCWPW1 in WT mouse testes and the ATAC signal in WT, *Spo11*^*–/–*^, *Prdm9*^*–/–*^, and *Zcwpw1*^*–/–*^testes. The blue shaded area represents the non-hotspot ATAC signal at promoter regions, while the orange shaded area represents the ATAC signal at hotspot sites. **b** Heatmap showing the ZCWPW1and DMC1 signals in WT testes and the ATAC signal in WT, *Spo11*^*–/–*^, *Prdm9*^*–/–*^, and *Zcwpw1*^*–/–*^ testes at two groups of ZCWPW1 binding sites (peaks). Group A1 indicates ZCWPW1 binding sites that lost the ATAC signal in *Zcwpw1*^*–/–*^ testes, while group A2 indicates ZCWPW1 binding sites that retained the ATAC signal in *Zcwpw1*^*–/–*^testes. The regions of group A1 or A2 in heatmaps were ordered from largest to smallest based on the median signal of ZCWPW1 ChIP-seq.

It is well-established that H3K4me3 and H3K9ac mark active promoters and modulate chromatin structure to promote transcription [57, 58]. Given that ZCWPW1 is known to bind H3K4me3, and considering that our data show a role for ZCWPW1 in preserving H3K9ac, we hypothesized that ZCWPW1 functions in the regulation of chromatin accessibility at recombination hotspots. In the ATAC-seq data from WT mouse testes, we found that 4015 of 14688 ZCWPW1 binding sites were open (the open ZCWPW1 binding site was indicated as the ZCWPW1 peak that overlapped with at least 20% of an ATAC peak) (Fig. 4b). Next, we compared the ATAC signal in WT and *Zcwpw1*^*−/−*^ testes. We found that 67% (2676/4015) (group A1) of the ZCWPW1 binding sites lost the ATAC signal in *Zcwpw1*^*−/−*^ mouse testes (Fig. 4a, b), indicating that ZCWPW1 regulates chromatin openness at ZCWPW1 binding sites, while 33% (1339/4015) (group A2) of the ZCWPW1 binding sites retained the ATAC signal in *Zcwpw1*^*−/−*^ mouse testes (Fig. 4b). We also found that 61.3% of the group A2 ZCWPW1 binding sites were located at transcript promoter regions, while only 4.7% of the group A1 ZCWPW1 binding sites were located at promoter regions (Additional file 1: Fig. S5b), Further, we found that both the ZCWPW1 and DMC1 signals at group A1 ZCWPW1 binding sites were much stronger than at group A2 sites (Fig. 4b and Additional file 1: Fig.S5a). These results suggest that chromatin accessibility at recombination hotspots is modulated by ZCWPW1.

We subsequently explored the potential association between ZCWPW1’s impact on chromatin accessibility and H3K9ac by measuring the overlap of the ZCWPW1 binding sites at which ATAC signals or H3K9ac peaks were lost in *Zcwpw1*^*−/−*^ mouse testes. We found that 84% (2250/2676) of the group A1 ZCWPW1 binding sites showed simultaneous reductions in chromatin accessibility and H3K9ac signal intensity (Additional file 1: Fig. S5c and S5d).

ZCWPW1 is known to function as a reader of histone methylation catalyzed by PRDM9, and our ATAC-seq data confirmed that the chromatin accessibility of group A1 ZCWPW1 binding sites is drastically reduced in *Prdm9*^*−/−*^ mouse testes (Fig. 4a, b and Additional file 1: Fig. S5a), which was consistent with previous studies [46, 63, 64]. In addition, we found that chromatin openness was independent of DSBs by comparing ATAC signals in WT and *Spo11*^*−/−*^ mouse testes (Fig. 4a, b and Additional file 1: Fig. S5a), which was also consistent with previous studies [46, 63, 64]. Taken together, these results suggest that chromatin openness at recombination hotspots is also dependent on both the histone methylation writer PRDM9 and the histone methylation reader ZCWPW1.

### Ectopic expression of the germ-cell-specific protein ZCWPW1 in human somatic cells is able to enhance DSB repair through homologous recombination

**Fig. 5:**
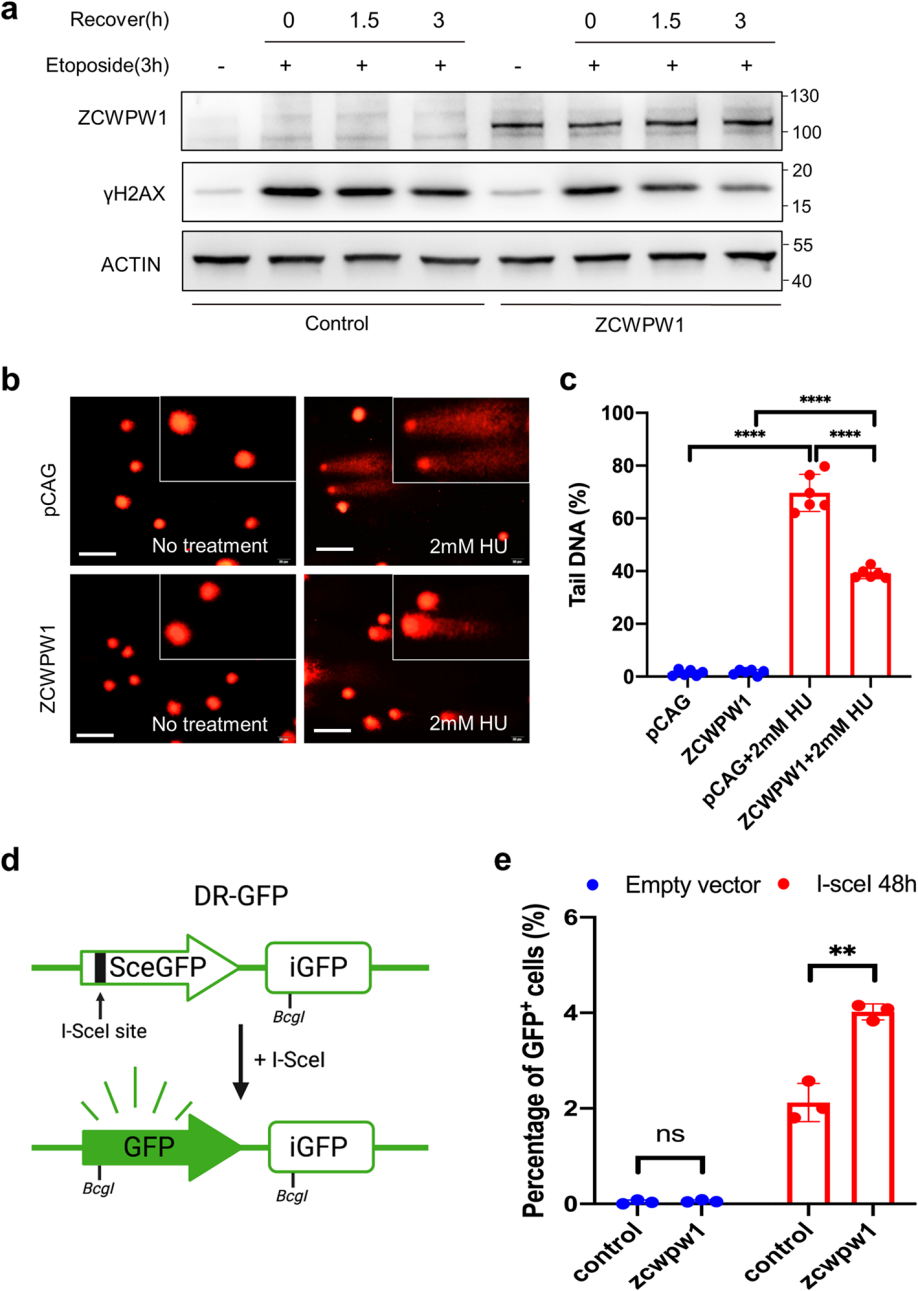
Ectopic expression of ZCWPW1 promotes DSB repair in human somatic cells. **a** After exposure to ETO for 3 h, the expression levels of γH2AX were measured by western blot in control and ZCWPW1-overexpressing HeLa cells. **b** Neutral comet assay to evaluate the extent of DSB repair in HEK293T cells treated with hydroxyurea (HU) for 16 h after transfection with pCAG or ZCWPW1-EGFP-pCAG for 24h. Scale bar, 20μm. **c** Bar plot showing the ratio of tail DNA in the neutral comet assays. ****P < 0.0001 by two-tailed unpaired Student’s *t* test. Data are representative of six independent experiments. **d** Schematic illustration of the GFP-based homologous recombination reporter assay. **e** Expression of ZCWPW1 promotes homologous recombination. U2OS DR-GFP cells expressing ZCWPW1 were electroporated with a pCBASce construct and were assayed for homologous recombination efficiency by monitoring GFP levels at 48 h post electroporation. **P < 0.01 by Student’s *t* test. Data represent the mean ± SEM from three independent experiments

It has been reported that histone acetylation and chromatin accessibility are necessary for DSB repair in somatic cells [65, 66]. Given the observed impacts of ZCWPW1 in regulating histone acetylation and chromatin accessibility, we tested whether ectopic expression of the germ-cell-specific protein ZCWPW1 in human cells affects DSB repair. We first expressed ZCWPW1 in HeLa cells and found that the length of the cell doubling time was comparable between the control and ZCWPW1 overexpression groups (Fig. S6a and S6b). We also performed a cell cycle assay using flow cytometry in control and ZCWPW1-overexpressing HeLa cells, and the proportion of cells in S-phase was comparable between the two groups (Additional file 1: Fig. S6c and S6d). To evaluate the DNA damage repair capacity between control and ZCWPW1-overexpressing HeLa cells induced by etoposide (ETO), γH2AX levels were measured by western blot as an indicator of DSBs. After exposure to ETO for 3 h, γH2AX levels were greatly increased in both control and ZCWPW1-overexpressing HeLa cells (Fig. 5a and Additional file 1: Fig. S6e). However, the clearing rate of γH2AX signals increased in HeLa cells expressing ZCWPW1 compared to Control (Fig. 5a and Additional file 1: Fig. S6e), which suggested that ZCWPW1 overexpression promotes the progression of DSB repair in HeLa cells.

We also expressed ZCWPW1 in HeLa cells and then treated them with Hydroxyurea to induce DSBs, and the cells expressing ZCWPW1 showed reduced fluorescence intensity for the DSB damage marker γH2AX (Additional file 1: Fig. S7a and S7b). Further, immunoblotting of cells treated with DSB-inducing agents (including hydroxyurea/camptothecin/ETO) showed that cells expressing ZCWPW1 had greatly reduced γH2AX levels compared to control cells, indicating that ZCWPW1 can enhance DSB repair (Additional file 1: Fig. S7c, S7d and S7e). In addition, we performed a neutral comet assay to evaluate the repair following agent-induced DSBs. Tail DNA was less abundant in the cells expressing ZCWPW1 than in control cells (Fig. 5b, c), indicating more severe DNA damage in the control cells. Taken together, these results confirmed that ZCWPW1 overexpression promotes the progression of DSB repair in HeLa cells.

It is well-known that the meiotic DSBs in germ cells are invariably repaired via homologous recombination, while the repair of DSBs in somatic cells rarely relies on homologous recombination. This phenomenon may indicate that there are several germ-cell-specific factors that contribute to the high efficiency of homologous recombination in the repair of meiotic DSBs. Thus, it is quite intriguing to ask whether expression of the germ-cell-specific protein factors in somatic cells might improve the rate of DSB repair via homologous recombination. To assess whether ZCWPW1 promotes DSB repair via homologous recombination, we used a previously constructed U2OS cell line with a chromosomally-integrated copy of the DR-GFP reporter to measure the repair efficiency of I-SceI-induced DSBs by homologous recombination [67, 68]. We expressed ZCWPW1 in these reporter cells and found that cells expressing ZCWPW1 displayed a significant increase in the frequency of homologous recombination compared to controls (Fig. 5d, e and Additional file 1: Fig. S7f). Taken together, ectopic expression of the germ-cell-specific protein ZCWPW1 in human somatic cells is able to enhance the efficiency of homologous recombination for DSB repair.

## Discussion

**Fig. 6:**
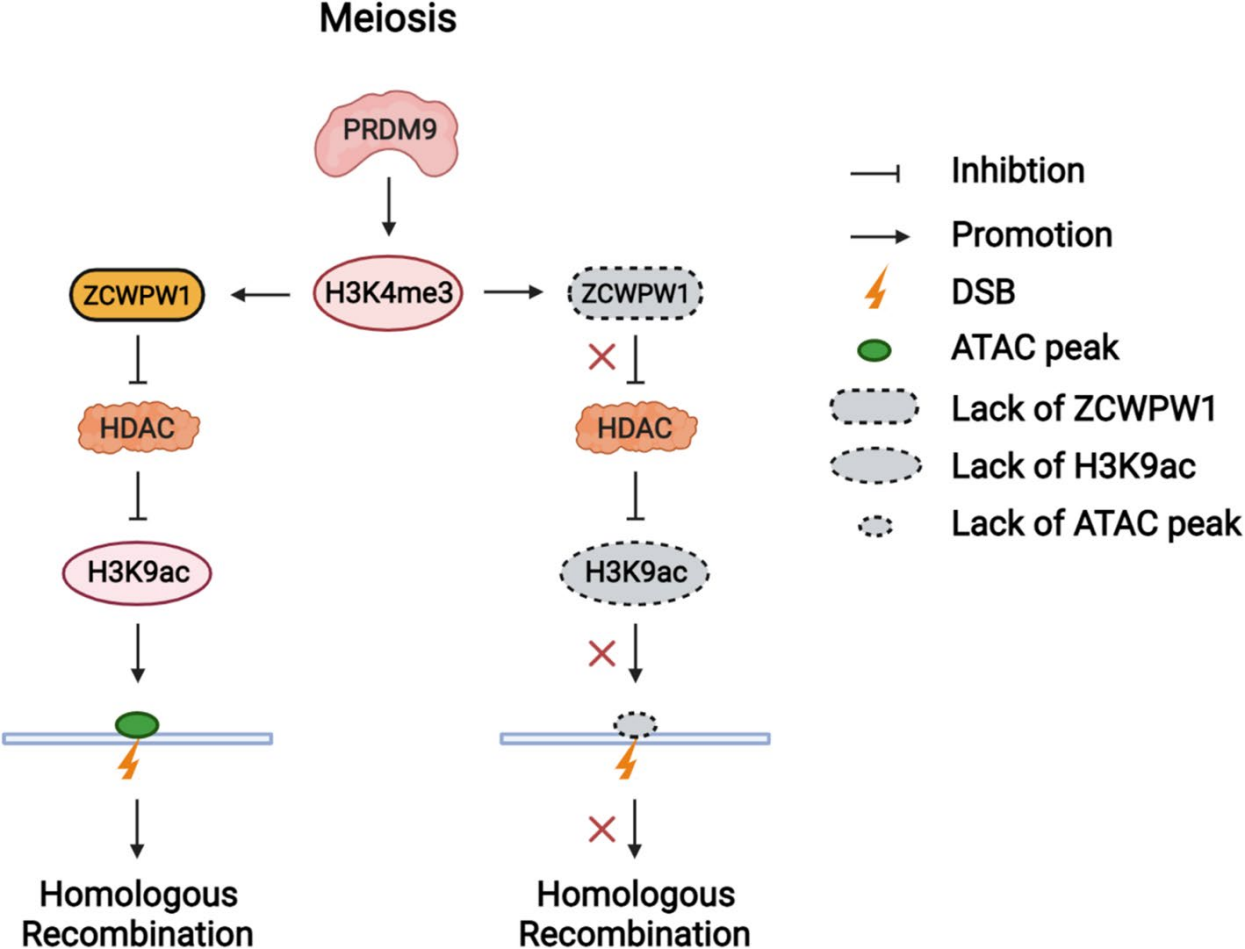
Model for how ZCWPW1 and PRDM9 regulate chromatin accessibility at homologous recombination hotspots during meiosis in mammals. PRDM9 deposits H3K4me3 at recombination hotspots, and ZCWPW1 recognizes H3K4me3 to protect H3K9ac by antagonizing HDAC activity. The retaining of H3K4me3 and H3K9ac create open chromatin surrounding PRDM9-binding sites. The working model was created with BioRender.com

PRDM9 writes H3K4me3 and H3K36me3 marks at meiotic hotspots via the methyltransferase activity of its PR/SET domain, thus specifying recombination hotspots in mammals [39]. In addition to H3K4me3 and H3K36me3, H3K9ac marks are enriched at meiotic hotspots [34, 42]. H3K4me3 and H3K9ac are established concurrently during the leptotene and zygotene stage, and they are removed at the pachytene stage [42]. It has been reported that H3K9ac at hotspot sites is independent of DSB formation but depends on PRDM9 methyltransferase activity [34, 46], suggesting the possibility of cross-talk between H3K4me3 and H3K9ac at recombination hotspots. However, it remains poorly understood which factors might mediate such cross-talk between histone methylation and acetylation at recombination hotspots. Our work shows that H3K9ac at recombination hotspots is dependent on both the histone methylation writer PRDM9 and the histone methylation reader ZCWPW1 (Fig. 6).

We originally assumed that ZCWPW1 regulates H3K9ac by recruiting histone acetylation writer proteins. However, the protein-protein interaction results from our ZCWPW1 RIME analysis did not indicate any interaction between ZCWPW1 and known histone acetylation writers. Unexpectedly, the histone methylation reader ZCWPW1 maintained H3K9ac at recombination hotspots during meiosis, specifically by antagonizing the deacetylation activities of HDAC proteins, which could partly explain how the histone methylation reader ZCWPW1 regulates histone acetylation at meiotic recombination hotspots. Nevertheless, it is still unknown how H3K9ac is established at recombination hotspots. Here, we speculate that PRDM9 recruits acyltransferases to deposit H3K9ac directly or through other factors that recognize PRDM9-catalyzed histone methylations.

The histone modification reader ZCWPW1 specifically recognizes dual histone methylation marks deposited by PRDM9 at hotspots in testes [47, 49, 50]. In *Zcwpw1*-null mice, the H3K9ac signal at hotspot sites was lost, while the H3K9ac signal at promoter regions was retained. Wells et al. found that ZCWPW1 was located at hotspot sites as well as numerous non-hotspot sites including promoter regions (which were enriched with either H3K4me3 or H3K36me3) in HEK293T cells ectopically expressing PRDM9 and ZCWPW1 [50]. In HeLa cells overexpressing ZCWPW1, the H3K9ac signal was increased, specifically, the H3K9ac signal overlapping the ZCWPW1 binding sites was significantly greater than that in the control group. These findings suggest that ZCWPW1 preserves H3K9ac by antagonizing HDAC activity at least partly due to its ability to recognize H3K4me3 and/or H3K36me3.

In mammals, meiotic homologous recombination hotspots are marked by a special combination of histone modifications, such as H3K4me3, H3K36me3 and H3K9ac [42, 46]. It has been reported that Prdm9-dependent recombination hotspot regions tend to be accessible regions of the chromatin [46, 64]. Remarkably, the chromatin accessibility at recombination hotspots precedes DSB formation but depends on PRDM9 [46, 63, 64]. Our findings indicated that the chromatin accessibility at recombination hotspots is dependent on both the histone methylation writer PRDM9 and the histone methylation reader ZCWPW1. It is known that the chromatin remodeling enzyme HELLS is recruited to hotspots through PRDM9 rather than being recruited as a consequence of PRDM9-dependent histone methylations [46]. Further, HELLS and PRDM9 form a pioneer complex to open chromatin at meiotic DSB hotspots and together regulate the DSB activity at hotspot sites [46, 56]. Considering that ZCWPW1 is recruited to meiotic hotspots based on PRDM9-dependent epigenomic modifications, it appears likely that the histone methylation reader ZCWPW1 functions to maintain chromatin openness rather than performing the initial opening of chromatin at meiotic hotspots.

It is well-established that H3K4me3 and H3K9ac at promoter regions are regarded as active histone modification marks, and these usually recruit chromatin remodeler proteins to make the promoter regions more accessible and thus regulate gene transcription [57, 58]. Both of these histone modification marks as well as chromatin accessibility are enriched at recombination hotspots, which are established concurrently during the leptotene and zygotene stages [42, 46, 64]. However, it remains unknown whether and how histone modifications affect chromatin accessibility at recombination hotspots during meiotic homologous recombination in mammals. In our study, we found that the histone methylation reader ZCWPW1 could promote chromatin openness, which may be due to its ability to maintain H3K9ac at recombination hotspots.

Both histone modification and chromatin remodeling are critical for DSB repair [65, 69–71], and it has been reported that PRDM9 might function to promote homolog-templated repair [72]. Previous studies found that DSB formation at hotspots is independent of ZCWPW1, but meiotic DSBs are not repaired following knockout of *Zcwpw1* [47–50], and it remains unclear how ZCWPW1 influences DSB repair. Here, we confirmed the requirement of ZCWPW1 to preserve H3K9ac and chromatin accessibility at recombination hotspots, and this might promote homologous recombination during meiotic DSB repair. In our RIME assay, several chromatin remodelers and DSB repair factors were also identified as potential partners of ZCWPW1, and some of these chromatin remodeling factors, such as SMARCA5 and CHD4, have been shown to promote chromatin relaxation in response to DNA damage [73–76]. The possible function of ZCWPW1 and its potential chromatin remodeler partners in the repair of meiotic DSBs represents a major question in meiosis that deserves further investigation.

Further, we have also found that the H3K4me3 peaks at hotspot regions could be detected in *Zcwpw1*^*−/−*^ testes, while these same H3K4me3 peaks are lost in *Prdm9*^*−/−*^ testes (Additional file 1: Fig. S8). Recall that meiotic DNA double-strand break formation is dependent on PRDM9 methyltransferase activity, that is, the H3K4me3 and H3K36me3 are marks deposited by PRDM9 [39, 43–45]. DSBs still occur at PRDM9-dependent hotspots in *Zcwpw1*^*−/−*^ testes, suggesting that the SPO11 machinery still has access to these sites to induce DSBs [49, 50]. So, these results support a working model wherein PRDM9 binds to hotspot sites and writes H3K4me3 and H3K36me3 [39, 43–45]. This leads to the recruitment of SPO11 machinery required for the formation of DSBs [77, 78]. After these PRDM9-catalyzed epigenetic modifications are deposited, ZCWPW1 can specifically read these H3K4me3 and H3K36me3 marks in the vicinity of DSB sites, where ZCWPW1 functions to maintain the H3K9ac and chromatin accessibility to promote DSB repair [42, 47].

Beyond the exploration of ZCWPW1’s roles in meiotic DSB repair, we also found that simple ectopic expression of the germ-cell-specific protein ZCWPW1 in somatic cells could elevate the H3K9ac signal at ZCWPW1 binding sites and promote DSB repair following treatment with DSB-inducing agents. Here, using a homologous recombination reporter assay, we confirmed that ZCWPW1 could improve the rate of DSB repair via homologous recombination. A recent study reported a simple strategy to improve homology-directed repair efficiency by engineering CRISPR-Cas9-methyltransferase fusion protein (PRDM9) [79]. These findings emphasize the importance of histone modification and chromatin structure for the choice of DSB repair pathway and provide a new strategy for increasing the homology-directed repair efficiency, thus showing the promise of gene editing.

## Conclusions

In summary, our study has shown that the histone methylation reader ZCWPW1 is able to mediate the cross-talk between histone methylation and histone acetylation and that it functions to promote chromatin openness at DSB hotspots, and together these roles of ZCWPW1 pave the way for homologous recombination during meiotic DSB repair (Fig. 6).

## Methods

### Mice

The *Zcwpw1* knockout mice and *Prdm9* knockout mice were generated in our previous studies [47, 48]. The mouse *Spo11* gene (GenBank accession number: NM_012046.2; Ensembl: ENSMUSG00000005883) is located on mouse chromosome 2 and comprises 13 exons, with the ATG start codon in exon 1 and the TAA stop codon in exon 13. Exon 2 to exon 12 were selected as the target site, which covered 79.12% of the coding region. The *Spo11* knockout mice were generated by deleting exon 2 to exon 12 using the CRISPR/Cas9-mediated genome editing system in the C57BL/6 genetic background (Cyagen Biosciences, Guangzhou, China).

The *Spo11* knockout founders were genotyped by PCR followed by DNA sequencing analysis. The homozygous mutant mice were generated by intercrossing of heterozygous mutants, and genotyping was performed by PCR amplification of genomic DNA extracted from mouse tails. PCR primers for the *Spo11* mutant allele were Forward: 5′-TAT CAA CTG GGG CCT GTG GTC AG-3′ and Reverse: 5′-GTG TGC AGG CTT GCG ACA GTG-3′, yielding a 440 bp fragment. PCR primers for the *Spo11* WT allele were Forward: 5′-TCC GTT CAG TGT GGT TCT CC-3′ and Reverse: 5′-GTG TGC AGG CTT GCG ACA GTG-3′, yielding a 222 bp fragment.

All mice were maintained with free access to water and food in a specific pathogen-free facility under a 12-h light/dark cycle. All experimental protocols were approved by the regional ethics committee of Shandong University.

### Antibodies

The primary antibodies used for ChIP-seq and CUT&TAG were rabbit anti-ZCWPW1 (1:100 dilution; homemade) [48] and rabbit anti-H3K9ac (1:100 dilution; Active motif #39917). Primary antibodies used for immunoprecipitation (IP) and western blotting (WB) were rabbit anti-ZCWPW1 (5 μg for IP, 1:1000 dilution for WB; homemade), rabbit anti-HDAC1 (5 μg for IP, 1:1000 dilution for WB; Proteintech #10197-1-AP), rabbit anti-HDAC2 (5 μg for IP, 1:1000 dilution for WB; Proteintech #12922-3-AP), rabbit anti-HDAC6 (5 μg for IP, 1:1000 dilution for WB; Proteintech #12834-1-AP), rabbit anti-GFP (5 μg for IP, 1:1000 dilution for WB; Proteintech # 50430-2-AP), rabbit anti-SMCHD1 (5 μg for IP, 1:1000 dilution for WB; Proteintech #25589-1-AP), normal rabbit IgG (5 μg for IP; Sigma-Aldrich #12-370), mouse anti-β actin (1:5000 dilution for WB; Proteintech #66009-1-Ig), mouse anti-phospho-histone H2AX (pSer139) (1:2000 dilution for WB; Millipore #05-636), mouse anti-H3K9ac (1:1000 dilution for WB; PTM-Bio #PTM-156), rabbit anti-H3K27ac (1:2000 dilution for WB; Active motif #39133), rabbit anti-H3K56ac (1:1000 dilution for WB; PTM-Bio #PTM-118), and rabbit anti-acetyl lysine (1:1000 dilution for WB; Abcam #ab190479). Primary antibodies used for immunocytology were mouse anti-phospho-histone H2AX (pSer139) (1:1000 dilution; Millipore #05-636) and rabbit anti-GFP (1:1000 dilution; Invitrogen #A-11122). Primary antibodies were detected with Alexa Fluor 488- or 594-conjugated secondary antibodies (1:500 dilution; Abcam #ab150077, #ab150120) for 1 h at room temperature. The slides were washed three times with PBS and mounted using mounting medium with DAPI (Abcam #ab104139).

### Constructs

Complementary DNA (cDNA) for mouse *Zcwpw1* and *Smchd1* was synthesized by Wuhan GeneCreate Biological Engineering. The *Zcwpw1* was cloned into pCAG-GFP (Addgene, #11150) with a C-terminal fusion EGFP tag or pcDNA3.1(+) with a C-terminal fusion HA-V5 tag for transient expression in cell lines. Mouse *Hdac1* (#P10549), *Hdac2* (#P5693), and *Hdac6* (#P10426) expression plasmids were purchased from Wuhan Miaoling Biological Technology. The I-SceI expression plasmid (pCBASce) used in the homologous recombination reporter assay was kindly provided by Professor Jun Huang from Zhejiang University. All constructs used in this study were confirmed by DNA sequencing.

### Cell culture and transfection

HeLa, HEK293T, and U2OS cells were cultured in Dulbecco’s modified Eagle’s medium (DMEM) supplemented with 10%(v/v) fetal bovine serum and 1% penicillin streptomycin at 37°C in a 5% CO_2_ incubator. For transient transfection experiments, cells were transfected with expression vectors using the X-tremeGENE HP DNA Transfection Reagent (Roche, 6366244001) following the manufacturer’s instructions. HeLa and HEK293T cells were purchased from the National Collection of Authenticated Cell Cultures, and U2OS cells were kindly provided by Professor Qiang Chen from Wuhan University. U2OS DR-GFP cell lines were kindly provided by Professor Jun Huang from Zhejiang University [67, 80].

### Homologous recombination reporter assays

Homologous recombination reporter assays were performed as described previously [67]. Briefly, U2OS DR-GFP cells were seeded into 6-well plates. After attachment, cells were transfected with 2 μg empty vector or ZCWPW1-HA-V5 in pcDNA3.1(+) for 24 h then transfected with 3 μg I-SceI expression plasmid (pCBASce). Cells were harvested after 48 h post pCBASce transfection and GFP expression was measured by flow cytometry analysis. Means were obtained from three independent experiments.

### Neutral comet assay

The neutral comet assay was performed using the Comet Assay Kit (Trevigen, #4250-050-K) according to the manufacturer’s instructions. Briefly, HEK293T cells were cultured in 6-well plates and transfected with pCAG (Addgene, #11150) or ZCWPW1-pCAG using Lipofectamin 3000 Transfection Reagent (Invitrogen, # L3000001) following the manufacturer’s protocol. After 24 h, cells were treated with 2 mM hydroxyurea for 16 h. A total of 1 × 10^4^ cells in 10 μl PBS were mixed with 100 μl 0.8% low-gelling agarose and were layered as microgels on microscope slides. Slides were then immersed in lysis solution at 4°C for 1–2 h in the dark. After lysis, the slides were incubated in alkaline unwinding solution for 20 minutes at room temperature. Electrophoresis was performed at neutral pH at 1 V/cm for 10–15 min. After neutralization, samples were stained with ethidium bromide for 10 min and imaged with an Olympus fluorescence microscope. DNA damage was quantified by measuring the comet tail length using Comet Assay Software Pect (CASP 1.2.3 beta 1).

### Co-IP and western blotting

Testes or transfected cells were lysed with Pierce IP lysis buffer (25 mM Tris-HCl pH 7.4, 150 mM NaCl, 1 mM EDTA, 1% NP-40, and 5% glycerin; Thermo Scientific #87787) with protease inhibitors (Roche, #04693132001) on ice for 30 min, then centrifuged at 12,000×*g* for 15 min at 4°C. The supernatant was transferred into a new tube and incubated with primary antibody or control IgG with rotation overnight at 4°C. Then, the antibodies were isolated by adsorption to Pierce protein A/G beads (Thermo Scientific, #88802) for 2 h. After washing, SDS loading buffer was added to the beads and boiled. Samples were separated by SDS-PAGE and immunoblotted with primary and secondary antibodies. Immunoreactive bands were detected and analyzed with a BIO-RAD ChemiDoc MP Imaging System and Image Lab Software (BIO-RAD, USA).

### Yeast two hybrid assay

The yeast two-hybrid assay was performed by GeneCreate Biological Engineering, Wuhan, China. Briefly, full length mouse *Zcwpw1* cDNA was subcloned into the pGBKT7 vector as bait. Full length mouse *Hdac1*, *Hdac2*, *Hdac6*, and *Smchd1* cDNAs were subcloned into the pGADT7 vector as prey. The bait and prey plasmids were co-transformed into the yeast two-hybrid gold strain yeast, and the positive transformants were selected on nutrition-restricted plates (SD/-Leu/-Trp/-His).

### Immunocytology

HeLa cells were cultured on coverslips transfected with pCAG (Addgene, #11150) or ZCWPW1-pCAG using X-tremeGENE HP DNA Transfection Reagent following the manufacturer protocol. After 24 h, cells were treated with 2mM Hydroxyurea for 16 h, 1 μM Camptothecin for 3 h, or 50 μM ETO for 6 h. Transfected cells on glass coverslips in 6-well plates were fixed in 4% paraformaldehyde in PBS pH 7.4 for 10 min at room temperature. The samples were incubated for 10 min with PBS containing 0.3% Triton X-100. After washing in PBS three times, the cells were blocked with 1% BSA and 22.52 mg/mL glycine in PBST (PBS+ 0.1% Tween 20) for 30 min at room temperature. Then, the samples were incubated with diluted primary antibody in 1% BSA in PBST in a humidified chamber for 2 h at room temperature or overnight at 4°C. After washing in PBS three times, the cells were incubated with secondary antibodies in 1% BSA for 1 h at room temperature in the dark. The coverslips were mounted with a drop of mounting medium containing DAPI. The coverslips were sealed with nail polish to prevent drying and movement under the microscope.

### Microscopy

Immunostained slides were imaged by confocal microscopy (Andor Dragonfly spinning disc confocal microscope driven by Fusion Software). Projection images were then prepared using Photoshop (Adobe) software packages or Bitplane Imaris (version 8.1) software. For live cell laser micro-point irradiation, U2OS cells were seeded in a glass-bottom dish (NEST, Cat# 801001). After attachment, the indicated expression plasmids were transfected into U2OS cells. Laser micro-point irradiation was conducted 24 h after transfection, and images were captured at the indicated time point. Laser micro point irradiation was performed under an inverted confocal microscope (Dragonfly, LeicaDMI8) with a micro-point laser workstation (Andor).

### HDAC activity assay

The HDAC activity assay was performed using the Histone Deacetylase Activity Assay Kit (ABCAM, #ab156064) according to the manufacturer’s instructions [81]. The HDAC activity assay is based on an AMC-conjugated acetylated peptide. AMC is a fluorescent dye whose fluorescence is quenched when conjugated to the acetylated peptide. Once the HDACs de-acetylate the acetylated peptide, it becomes susceptible to cleavage by an enzyme (the Developer component), which results in releasing free AMC, the fluorescence of which can be measured by a fluorescence microplate reader (Ex/Em 355/460 nm). Briefly, HeLa cells were cultured in a 6-well plate transfected with indicated plasmids using X-tremeGENE HP DNA Transfection Reagent following the manufacturer’s protocol. After 24 h, 10 μl nuclear extracts from transfected HeLa cells were mixed with HDAC assay buffer, substrate, and developer and then incubated at room temperature for 2 h. Fluorescence intensity was measured with a Perkin Elmer EnVision (Excitation 355 nm, Emission 460 nm).

### Rapid immunoprecipitation mass spectrometry of endogenous proteins (RIME)

RIME is a technique ideally suited for the identification of transcriptional co-factors and chromatin-associated proteins [82]. ZCWPW1-interacting proteins at chromatin were interrogated by RIME at Active Motif. Briefly, cells from PD13–14 or PD16–PD18 WT mouse testes were crosslinked in 1% formaldehyde for 15 min and then quenched with 125 mM glycine for 5 min. Cells were lysed and sonicated to shear the DNA to an average length of 300–500 bp. ZCWPW1 was immunoprecipitated from 100 mg of chromatin using rabbit anti-ZCWPW1 antibody or normal rabbit IgG, followed by pull-down using protein A/G magnetic beads. Protein complexes were digested with trypsin, and digested peptides were analyzed by LC-MS/MS.

### RIME data analysis

RIME analysis was performed according to published protocols [59] using ZCWPW1 and IgG antibodies. Briefly, The RIME raw data were processed using Proteome Discoverer (v1.4) and Mascot and/or SEQUEST as search engines. The identified proteins were filtered according to their false discovery rate and spectral count. Only the proteins with -10lgP≥20 and at least 1 unique peptide were retained. Moreover, to improve the confidence of the identified proteins, only proteins with at least 5 spectral counts were considered as positive. To exclude non-specificity, the proteins identified in the ZCWPW1 antibody group should not be identified in the negative IgG controls in the same batch experiments.

### ChIP-seq library preparation and sequencing

The ChIP-seq libraries were prepared as previously described [47] with further modifications primarily for DNA purification. In brief, 2 × 10^6^ cells from PD13–PD14 testes were cross-linked in 100 μl of 1% formaldehyde in PBS at room temperature for 10 min and then quenched with 25 μl 1.25M glycine solution and washed with PBS. The cells were then incubated in 150 μl lysis buffer (50 mM Tris-HCl pH 8.0, 10 mM EDTA pH8.0, 0.5% SDS, 1mM PMSF, and 1× proteinase inhibitor cocktail) for 20 min on ice then sonicated using a Diagenode Bioruptor sonication device for 23 cycles (30s ON and 30s OFF). A total of 150 μl 300mM SDS-free RIPA buffer (10 mM Tris-HCl pH 7.5, 300 mM NaCl, 1 mM EDTA, 0.5 mM EGTA, 1% Triton X-100, 0.1% Na-deoxycholate, 1mM PMSF, 1×proteinase inhibitor cocktail, and 20 mM Na-butyrate) and 200 μl 140mM SDS-free RIPA buffer was added to the samples. After centrifugation at 13,000×*g* for 10 min at 4°C, 40 μl of the supernatant was removed and used as the sample input. The remaining supernatant was transferred to a 1 ml tube containing suspended antibody-coated Protein A beads, followed by incubation on a tube rotator overnight at 4°C. Next, the incubated Protein A beads were washed once with RIPA buffer containing 250 mM NaCl, washed three times with RIPA buffer containing 500 mM NaCl, and washed once with TE buffer (10 mM Tris-HCl pH 8.0, 1mM EDTA). Next, the beads were transferred to a new 0.5 ml tube, followed by incubation in 100 μl ChIP elution buffer (10mM Tris-HCl pH8.0, 5mM EDTA, 300mM NaCl, and 0.5% SDS) containing 5 μl proteinase K (Qiagen, 20mg/ml stock) at 55°C for 2 h and then at 65°C for 4 h. The eluate was transferred to a fresh 0.5 mL tube, and the enriched DNA was purified by 1.8X SPRIselect beads, followed by dissolution in 50 μl TE buffer. Finally, the NEBNext Ultra II DNA Library Prep Kit for Illumina (NEB, E7645S) was used for library construction according to the product instructions. Libraries were sequenced using the Illumina X-ten and NovaSeq 6000 platform in PE150 mode (Novogene, Beijing, China).

### Cleavage under targets and tagmentation (CUT&Tag) assay

The CUT&Tag assay was performed as previously described [83] with modifications using the Hyperactive Universal CUT&Tag Assay Kit for Illumina (Vazyme Biotech, #TD903). Briefly, 1 × 105 cells were washed with 500 μl wash buffer and centrifuged at 600×*g* for 5 min at room temperature. Cell pellets were resuspended with 100 μl wash buffer. A total of 10 μl concanavalin A-coated magnetic beads were washed twice with 100 μl binding buffer and then added to the cell tubes and incubated at room temperature for 10–15 min. After removing the supernatant, the bead-bound cells were resuspended with 50 μl antibody buffer containing 3 μg rabbit anti-ZCWPW1 antibody or 2 μg rabbit anti-H3K9ac antibody. After incubation at room temperature for 2 h or overnight at 4°C, the primary antibody was carefully discarded and 0.5 μl Guinea pig anti-rabbit IgG (4A BIOTECH, #ABIN101961) diluted with 50 μl Dig-wash buffer was added to the cells. The cells were then incubated with rotation at room temperature for 1 h. The cells were then washed gently with 200 μl Dig-wash buffer three times, and 2 μl pA/G–Tnp together with 98 μl Dig-300 buffer was added to the samples. After incubating at room temperature for 1 h, the samples were washed gently with 200 μl Dig-300 buffer three times. Then, 10 μl 5 × TTBL mixed with 40 μl Dig-300 buffer was added to each sample and the samples were incubated at 37 °C for 1 h. The interactions were quenched by adding 5 μl 20 mg/ml Proteinase K, 100 μl Buffer L/B, and 20 μl DNA extraction beads and incubating the samples at 55°C for 10 min. The supernatant was discarded and the beads were washed once with 200 μl Buffer WA and twice with 200 μl Buffer WB and resuspended with 22 μl nuclease free water. For library amplification, 15 μl of purified DNA was mixed with 25 μl of 2× CAM, along with 5 μl of uniquely barcoded i5 and i7 primers from the TruePrep Index Kit V2 for Illumina (Vazyme Biotech, # TD202). A total volume of 50 μl of sample was placed in a thermal cycler (BIO-RAD, #T100) using the following program: 72 °C for 3 min; 98 °C for 3 min; 12–16 cycles of 98 °C for 10 s, 60 °C for 5 s, and 72°C for 1 min; and holding at 12 °C. To purify the PCR products, 2× volumes of VAHTS DNA Clean Beads (Vazyme Biotech, #N411) were added and incubated at room temperature for 5 min. The beads were washed twice with 200 μl fresh 80% ethanol and eluted in 22 μl ddH2O. All CUT&Tag libraries were sequenced by Novogene using the Illumina NovaSeq 6000 platform in PE150 mode (Novogene, Beijing, China).

### ATAC-seq library preparation and sequencing

The ATAC-seq libraries of mouse testes were prepared as previously described [84, 85] with minor modifications using the ATAC-Seq Assay Kit for Illumina (Novoprotein, #N248). Briefly, 0.5–1.5×10^5^ cells were washed with 500 μl PBS and centrifuged at 600×*g* for 5 min at room temperature. Cell pellets were lysed in 50 μl lysis buffer for 5 min on ice to prepare the nuclei. Immediately after lysis, 950 μl wash buffer was added and mixed thoroughly, and the tubes were centrifuged at 500×*g* at 4°C for 5 min to remove the supernatant. Nuclei were then incubated with the 40 μl Tn5 transposome and tagmentation buffer at 37°C for 30min. To end the tagmentation, 10 μl stop buffer was added directly into the reaction. To purify the DNA, 2× volumes of Novoprotein Tagment DNA extraction beads (Novoprotein, #N245) were added and incubated at room temperature for 5 min. The beads were washed twice with 200 μl fresh 80% ethanol and eluted in 37 μl elution buffer. For library amplification, 35 μl of purified DNA was mixed with 10 μl 5× Amplimix along with 2.5 μl of uniquely barcoded i5 and i7 primers from NovoNGS Index Kit for Illumina (Novoprotein, #N239B). A total volume of 50 μl of sample was placed in a Thermal Cycler (BIO-RAD, #T100) with the following program: 72 °C for 3 min; 98 °C for 30 s; 12–15 cycles of 98°C for 15 s, 60°C for 15 s, and 72°C for 8 s; 72°C for 2 min; and holding at 12°C. To purify the PCR products, 1.2× volumes of Novoprotein DNA Clean Beads (Novoprotein, #N240) were added and incubated at room temperature for 5 min. The beads were washed twice with 200 μl fresh 80% ethanol and eluted in 20 μl elution buffer. All ATAC-seq libraries were sequenced using the Illumina NovaSeq 6000 platform in PE150 mode.

### ChIP-seq bioinformatics analysis

The ChIP-seq raw reads were cropped to 100 bp, and the low-quality reads were removed using Trimmomatic v0.32 [86]. Paired reads were mapped to the mouse genome (version mm10) by Bowtie2 v2.3.4.2 with parameters “-X 2000 -no-discordant –no-contain” [87]. Reads with low mapping quality (MAPQ < 10) and PCR duplicated reads were removed by Samtools and Picard [88, 89]. Reads of two replicates were merged to call the necessary peaks using MACS2 v2.1.0 [90] with the parameters “ --SPMR -q 0.05 --nomodel” for H3K9ac and “ --SPMR -p 0.001 --nomodel” for ZCWPW1. The fold enrichment for ZCWPW1 peaks against random Poisson distribution with lambda should be large than 3. The normalized signals of H3K9ac, ZCWPW1, H3K4me3, and DMC1 were indicated as Fold Change for the treatment over input lambda using macs2 bdgcmp, and the signals were transformed into Bigwig using bedGraphToBigWig. ChIP-seq signal tracks were visualized by Integrative Genomics Viewer (IGV) [91]. Deeptools2 [92] computeMatrix was used to calculate the normalized signal of each 40 bp bin in the regions of the peak center ± 2k bp. Deeptools plotHeatmap, plotProfile, and R (3.4.4) were used to generate the profile plot and heatmap. ZCWPW1 peaks that overlapped with H3K9ac peaks by at least 1 bp were consider the ZCWPW1 binding sites marked with H3K9ac.

### CUT&Tag bioinformatics analysis

The CUT&Tag raw reads were cropped to 40 bp, and the low-quality reads were removed using Trimmomatic v0.32 [86]. Paired reads were mapped to the human genome (version hg19) by Bowtie2 v2.3.4.2 with parameters “-X 2000 -no-discordant –no-contain” [87]. Reads with low mapping quality (MAPQ < 10) and PCR duplicated reads were removed by Samtools and Picard [88, 89]. The CUT-TAG peaks were called by using MACS2 v2.1.0 [90] with parameters “--SPMR -B -q 0.01 --nomodel --nolambda --extsize 200”. The fold enrichment for the peaks against random Poisson distribution with lambda should be large than 10. The normalized signals of CUT-TAG were indicated as Fold Change of the treatment over lambda control (whole genome) using macs2 bdgcmp and were transformed into Bigwig using bedGraphToBigWig. ATAC-seq signal tracks were visualized by Integrative Genomics Viewer (IGV).

### ATAC-seq bioinformatics analysis

The ATAC-seq raw reads were cropped to 40 bp, and the low-quality reads were removed using Trimmomatic v0.32 [86]. Paired reads were mapped to the mouse genome (version mm10) by Bowtie2 v2.3.4.2 with parameters “-X 2000 -no-discordant –no-contain” [87]. Reads with low mapping quality (MAPQ < 10) and PCR duplicated reads were removed by Samtools and Picard [88, 89]. Reads of two replicates were merged to call the necessary peaks by using MACS2 v2.1.0 [90] with parameters “--SPMR -B -q 0.01 --nomodel --nolambda --extsize 200”. The fold enrichment for the peaks against random Poisson distribution with local lambda should be large than 4. The normalized signals of ATAC-seq were indicated as fold change of the treatment over lambda control (whole genome) using macs2 bdgcmp and were transformed into Bigwig by bedGraphToBigWig. ATAC-seq signal tracks were visualized by Integrative Genomics Viewer (IGV) [91]. Deeptools2 [92] computeMatrix was used to calculate normalized signal of each 40 bp-size bin in the regions of the peak center ± 2k bp. Deeptools plotHeatmap, plotProfile, and R (3.4.4) were used to generate the profile plot and heatmap. The open ZCWPW1 binding site was indicated as the ZCWPW1 peak that overlapped with at least 20% of an ATAC peak. It was considered that the openness of a ZCWPW1 binding site was greatly reduced or lost in ZCWPW1 knockout testes if a ZCWPW1 binding site had an ATAC peak in WT testes and the ATAC signal in WT was at least 2-fold greater than in ZCWPW1 knockout.

### Statistics

Statistical analysis was carried out with GraphPad Prism 6. Unpaired *t* tests were used to analyze differences between two groups. All tests and *p*-values are provided in the corresponding legends and/or figures.

## Supplementary Information

Additional file 1: Figure S1. ZCWPW1 binds at hotspot sites prior to DSB formation. Figure S2. Genome-wide properties of ZCWPW1-associated H3K9ac signal. Figure S3. Confirmation of ZCWPW1 binding proteins in mouse testes by Co-IP and yeast two hybrid. Figure S4. ZCWPW1 preserves the H3K9ac signal. Figure S5. Genome-wide properties of the ZCWPW1-associated ATAC signal. Figure S6. Ectopic expression of ZCWPW1 did not affect the cell cycle or doubling time. Figure S7. Ectopic expression of ZCWPW1 promotes DSB repair in somatic cells. Figure S8. H3K4me3 peaks at hotspot regions could be detected in *Zcwpw1*^*−/−*^ testes. Figure S9. Uncropped western blot gel images in Fig. 2d, e and f. Figure S10. Uncropped western blot gel images in Fig. 3a, b and 5a. Figure S11. Uncropped western blot gel images in Figure S3a-S3d and S4a-S4c. Figure S12. Uncropped western blot gel images in Figure S6a and S7c-S7f.

Additional file 2: Table S1. Proteins that putatively interact with ZCWPW1 were detected in RIME assay.

Additional file 3: Table S2. Summary of all ChIP-seq, ATAC-seq and CUT&TAG experiments performed, indicating target, sample, genotype and data source.

Additional file 4. Review History.

## Data Availability

Data generated in this study have been deposited in the Genome Sequence Archive (GSA) with the accession number CRA005763 and HRA002599. The ChIP-seq data of ZCWPW1 in WT and H3K4me3 in WT and *Prdm9*^*−/−*^ mouse testes were downloaded from GSA (CRA002088) [47, 49]. The H3K9ac data for Spermatocyte SCP3^+^(positive) H1T^-^(negative) spermatocytes were downloaded from GSE (GSE121760) [42]. The DMC1 SSDS sequencing data were downloaded from GSE (GSE93955) [44]. All dataset information are summarized in Additional file 3: Table S2.
