# Supplementary material for: The histone modification reader ZCWPW1 promotes double-strand break repair by regulating cross-talk of histone modifications and chromatin accessibility at meiotic hotspots
Source: Genome Biol. 2022 Sep 6;23:187. doi: 10.1186/s13059-022-02758-z (PMC9446545; doi:10.1186/s13059-022-02758-z)
Supplement: Supplementary file 1 — Additional file 1: Figure S1. ZCWPW1 binds at hotspot sites prior to DSB formation. Figure S2. Genome-wide properties of ZCWPW1-associated H3K9ac signal. Figure S3. Confirmation of ZCWPW1 binding proteins in mouse testes by Co-IP and yeast two hybrid. Figure S4. ZCWPW1 preserves the H3K9ac signal. Figure S5. Genome-wide properties of the ZCWPW1-associated ATAC signal. Figure S6. Ectopic expression of ZCWPW1 did not affect the cell cycle or doubling time. Figure S7. Ectopic expression of ZCWPW1 promotes DSB repair in somatic cells. Figure S8. H3K4me3 peaks at hotspot regions could be detected in Zcwpw1−/− testes. Figure S9. Uncropped western blot gel images in Fig. 2d, e and f. Figure S10. Uncropped western blot gel images in Fig. 3a, b and 5a. Figure S11. Uncropped western blot gel images in Figure S3a-S3d and S4a-S4c. Figure S12. Uncropped western blot gel images in Figure S6a and S7c-S7f. [file 13059_2022_2758_MOESM1_ESM.docx]

**
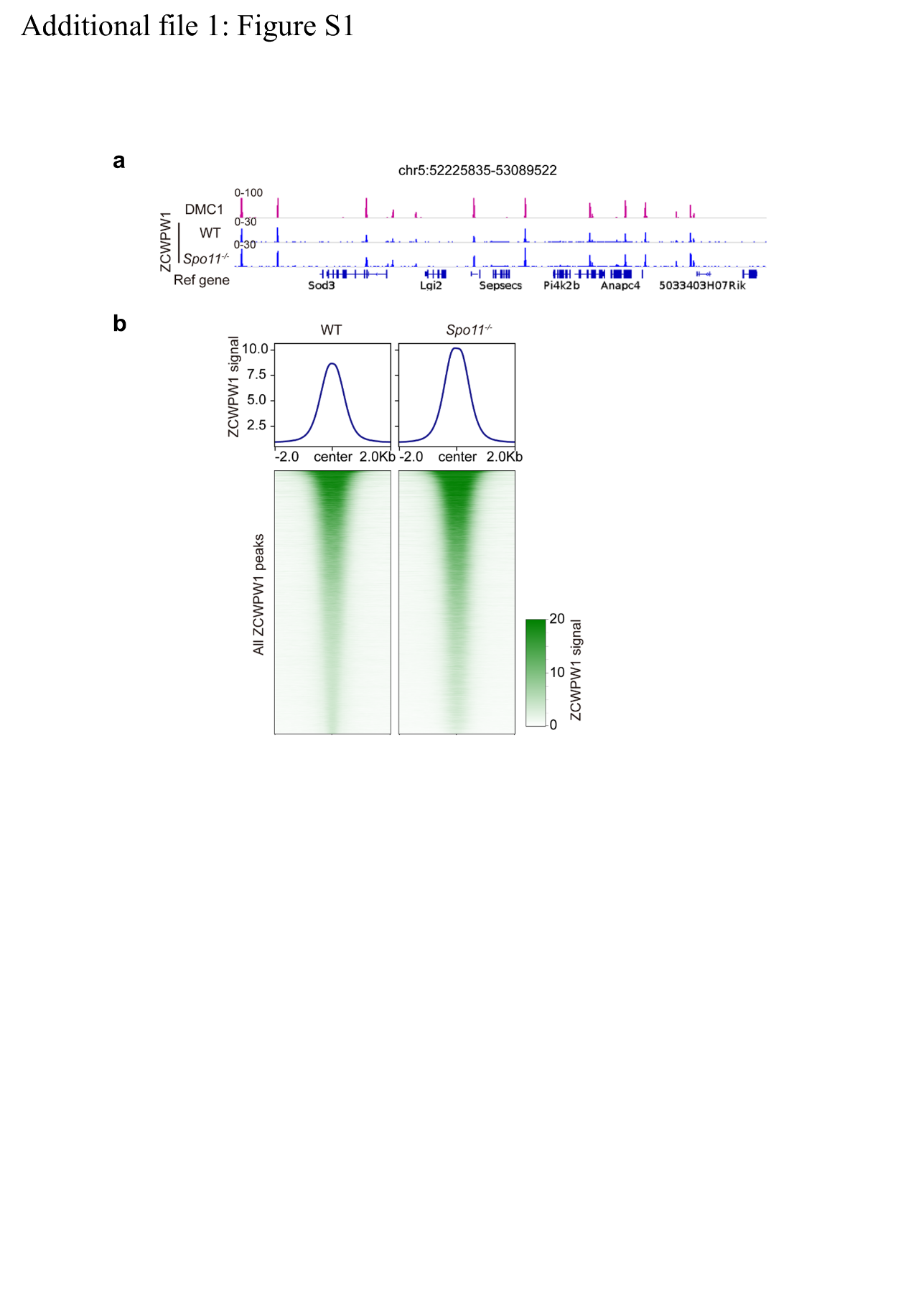
**

**Figure S1. Zcwpw1 binds at hotspot sites prior to DSB formation**

**(a)** Genome browser view of the DMC1 signal in WT mouse testes and the ZCWPW1 signal in WT and *Spo11^–/–^* testes. **(b)** Profile and heatmap showing enrichment of the ZCWPW1 signal in WT and *Spo11^–/–^* testes. The regions in heatmaps were ordered from largest to smallest based on the average signal in all samples.

**
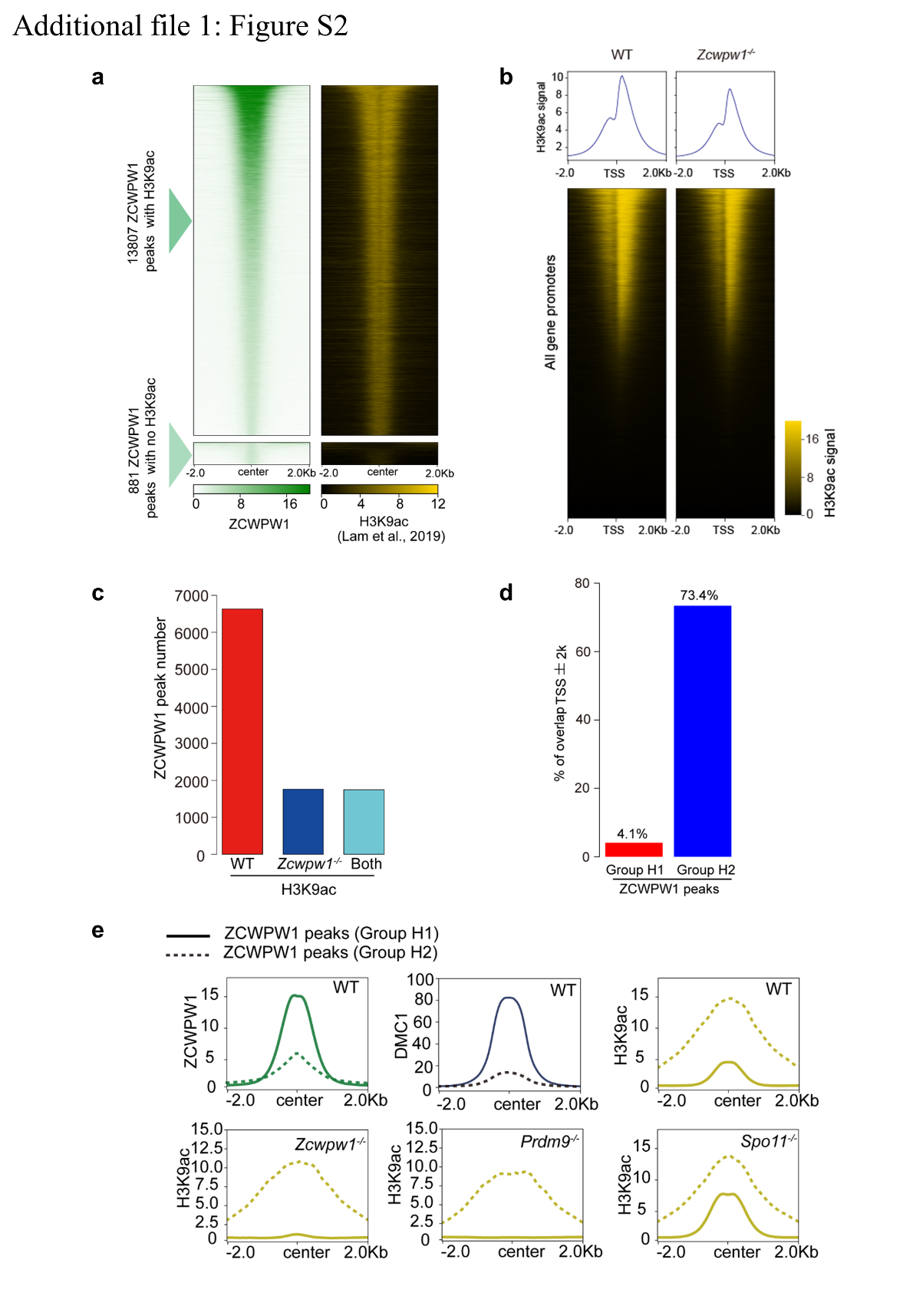
**

**Figure S2. Genome-wide properties of ZCWPW1-associated H3K9ac signal**

**(a)** Heatmap showing that the ZCWPW1 binding sites were enriched with H3K9ac signal in the leptotene and zygotene spermatocytes. The regions in heatmaps were ordered from largest to smallest based on the average signal in all samples. **(b)** Profile plot and heatmap showing the H3K9ac signal at all promoters in WT and *Zcwpw1^–/–^* testes (TSS±2kb). The regions in heatmaps were ordered from largest to smallest based on the average signal in all samples. **(c)** Bar plot showing the number of ZCWPW1 peaks (binding sites) that overlapped with H3K9ac peaks in WT and *Zcwpw1^–/–^* testes separately, as well as both overlapped with H3K9ac peaks in WT and *Zcwpw1^–/–^* testes. **(d)** Bar plot showing the proportion of the two groups (Group H1 and H2) ZCWPW1 peaks at promoter regions (TSS±2kb). **(e)** Profile plot of the average signal of ZCWPW1 and DMC1 in WT testes and the H3K9ac signal in WT, *Spo11^–/–^*, *Prdm9^–/–^,* and *Zcwpw1^–/–^* testes at two groups of ZCWPW1 binding sites (peaks). Group H1 indicates ZCWPW1 binding sites that lost the H3K9ac signal in *Zcwpw1^–/–^* testes, while Group H2 indicates ZCWPW1 binding sites that retained the H3K9ac signal in *Zcwpw1^–/–^* testes.

**
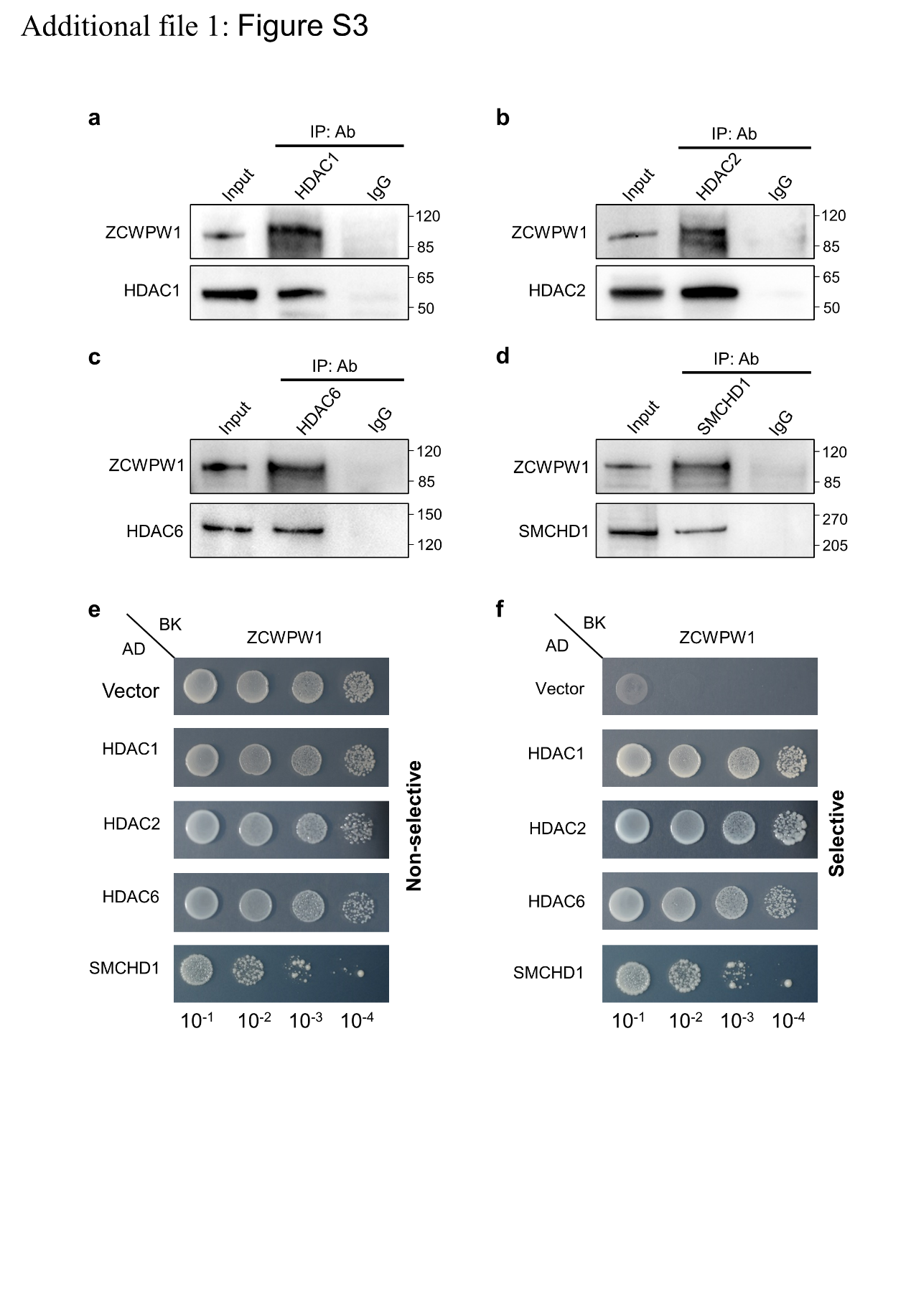
**

**Figure S3. Confirmation of ZCWPW1 binding proteins in mouse testes by Co-IP and yeast two hybrid**

**(a-d)** Co-IP analysis of HDAC1, HDAC2, HDAC6, and SMCHD1 with ZCWPW1 from PD14–PD16 testes protein extracts. ZCWPW1 was immunoprecipitated with HDAC1 (a), HDAC2 (b), HDAC6 (c), and SMCHD1 (d). Data are representative of three independent experiments. **(e-f)** Yeast two hybrid interactions. ZCWPW1was used as prey, and HDAC1, HDAC2, HDAC6, and SMCHD1 were used as bait. Data are representative of three independent experiments.


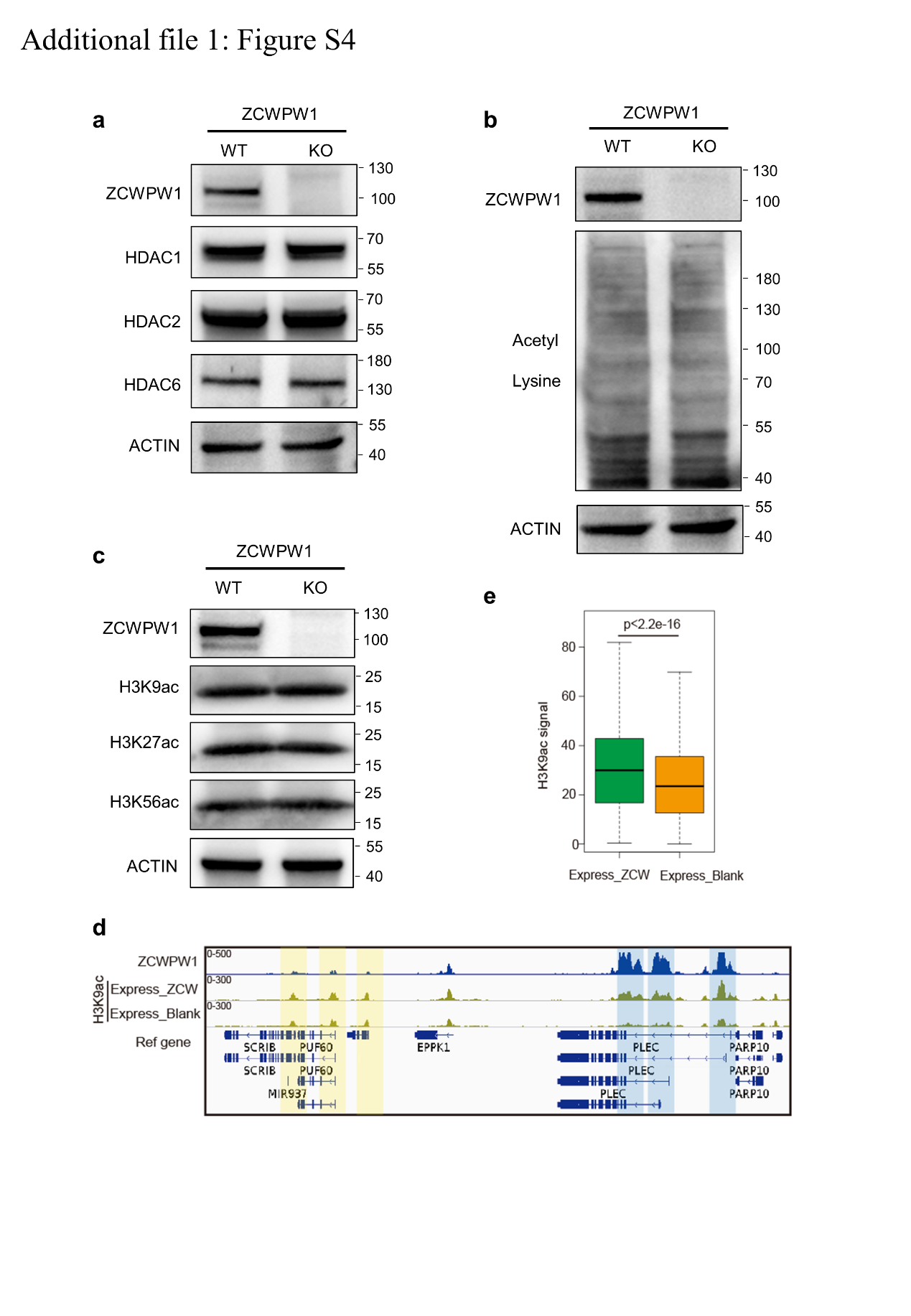


**Figure S4. ZCWPW1 preserves the H3K9ac signal.**

**(a-c)** Immunoblot analysis of ZCWPW1-interacting HDACs (a), acetyl Lysine (b) and histone H3 acetylation (K9, K27, K56) (c) in PD14 WT and *Zcwpw1^–/–^* testes. Data are representative of three independent experiments. **(d)** Genome browser view of the CUT&TAG signal of ZCWPW1 and H3K9ac in HeLa cells transfected with ZCWPW1 plasmid and control (blank). **(e)** Boxplot to compare the H3K9ac signal at ZCWPW1 peaks in HeLa cells transfected with ZCWPW1 plasmid and control (blank). The Wilcoxon rank sum test was used.

**
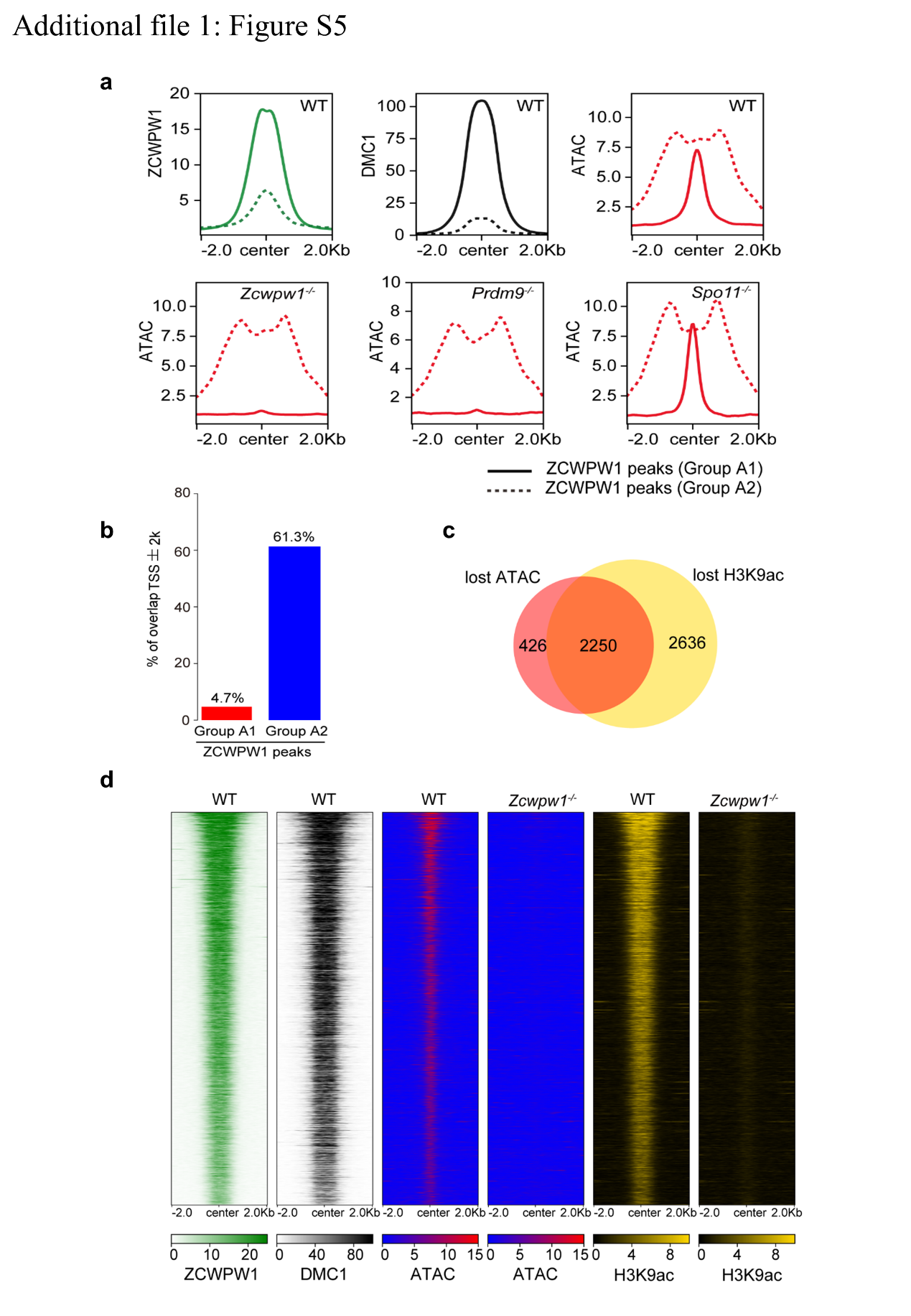
**

**Figure S5. Genome-wide properties of the ZCWPW1-associated ATAC signal**

**(a)** Profile plot of the average ZCWPW1 and DMC1 signals in WT testes and the ATAC signal in WT, *Spo11^–/–^*, *Prdm9^–/–^*, and *Zcwpw1^–/–^* testes at two groups ZCWPW1 binding sites (peaks). Group A1 indicates ZCWPW1 binding sites that lost the ATAC signal in *Zcwpw1^–/–^* testes, while Group A2 indicates ZCWPW1 binding sites that retained the ATAC signal in *Zcwpw1^–/–^* testes. **(b)** Bar plot showing the proportion of ZCWPW1 peaks in the two groups (Group A1 and A2) at promoter regions (TSS±2kb). **(c)** Venn diagram showing the overlap between the ZCWPW1 binding sites at which ATAC signals and H3K9ac signals are lost in *Zcwpw1^–/–^* testes. **(d)** Heatmap showing the ZCWPW1 and DMC1 signals in WT testes and ATAC and H3K9ac signals in WT and *Zcwpw1^–/–^* testes at ZCWPW binding sites where the loss of ZCWPW1 affected both the ATAC and H3K9ac signals. The regions in heatmaps were ordered from largest to smallest based on the median signal of ZCWPW1 ChIP-seq.

**
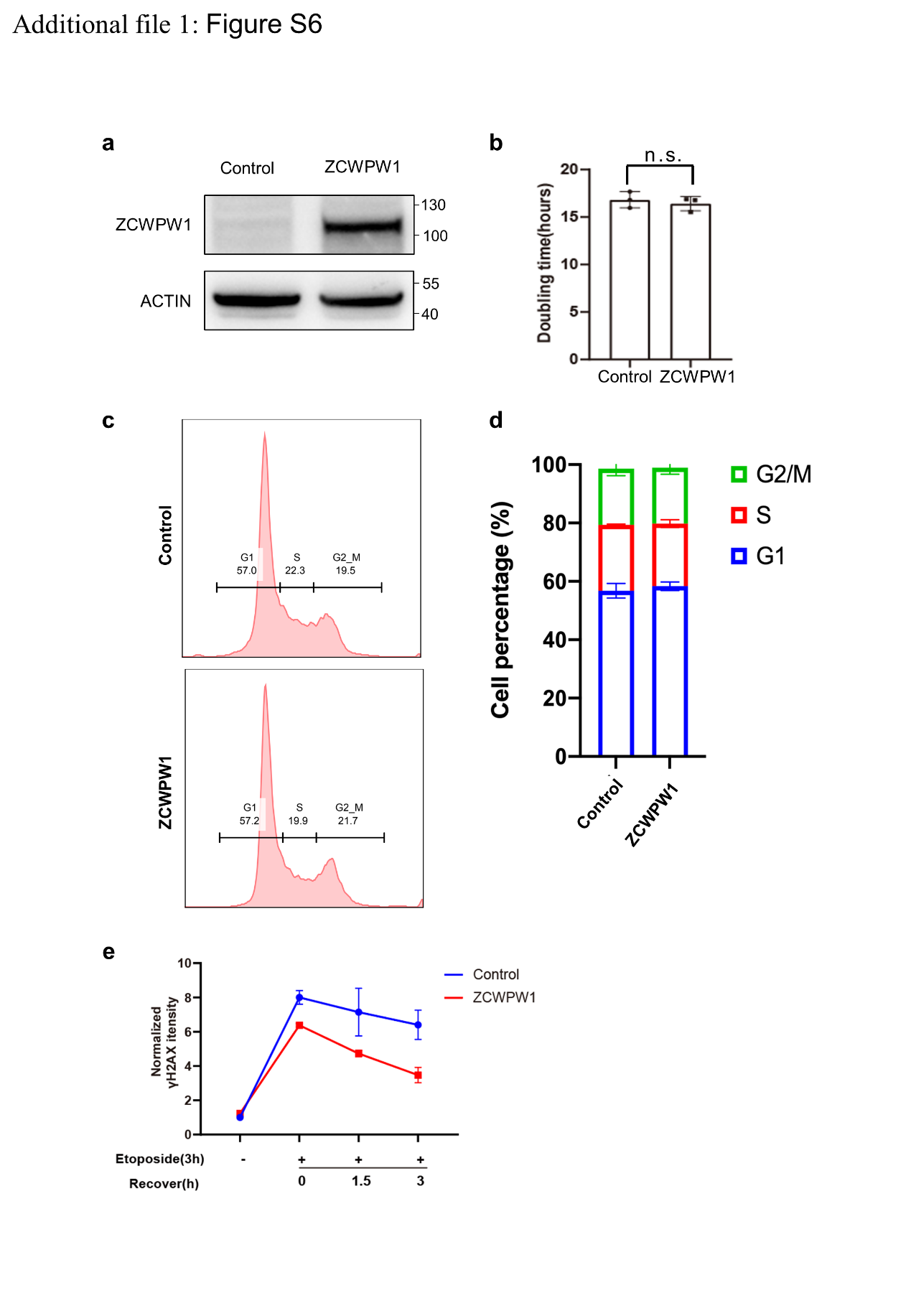
**

**Figure S6. Ectopic expression of ZCWPW1 did not affect the cell cycle or doubling time**

**(a)** Immunoblot analysis of ZCWPW1 in HeLa cells transfected with ZCWPW1 or control plasmid. **(b)** Bar plot showing the doubling time of HeLa cells transfected with ZCWPW1 or control plasmid. Data are representative of three independent experiments. **(c-d)** The cell cycle was determined by flow cytometry assay in control and ZCWPW1-overexpressing HeLa cells. The proportion of cells in S-phase was comparable between the two groups. **(e)** Quantitation of γH2AX signals corresponding to recover time. The clearing rate of γH2AX signals increased in HeLa cells expressing ZCWPW1 compared to Control.

**
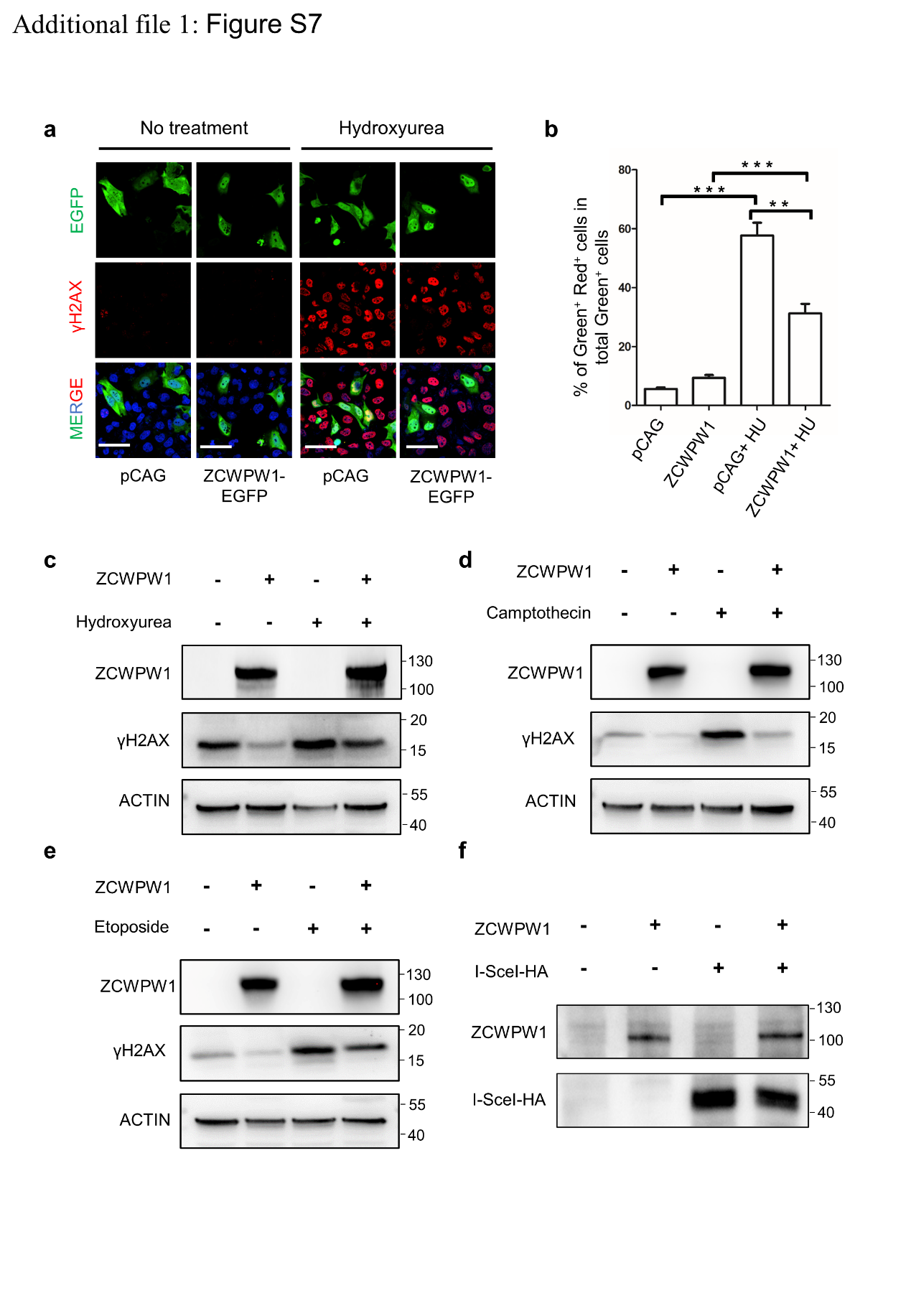
**

**Figure S7. Ectopic expression of ZCWPW1 promotes DSB repair in somatic cells**

**(a)** HeLa cells expressing pCAG or ZCWPW1-EGFP-pCAG were treated with hydroxyurea for 13 h, then stained by anti-EGFP and anti-γH2AX antibodies, and representative fluorescence images are shown. Scale bar, 30μm. **(b)** Bar plot showing the quantification of Green^+^ Red^+^ cells out of the total Green^+^ cells. **P < 0.01, ***P < 0.001 by two-tailed unpaired Student’s *t*-test. **(c-e)** Immunoblotting of ZCWPW1 and γH2AX in HeLa cells transfected with pCAG or ZCWPW1-EGFP-pCAG for 24 h, after exposure to the DSB-inducing agents Hydroxyurea (HU) for 13 h, Camptothecin for 3 h, or Etoposide for 6 h. Data are representative of three independent experiments. **(f)** Immunoblotting of ZCWPW1 and I-SceI-HA in DR-GFP U2OS cells**.** Data are representative of three independent experiments.

**
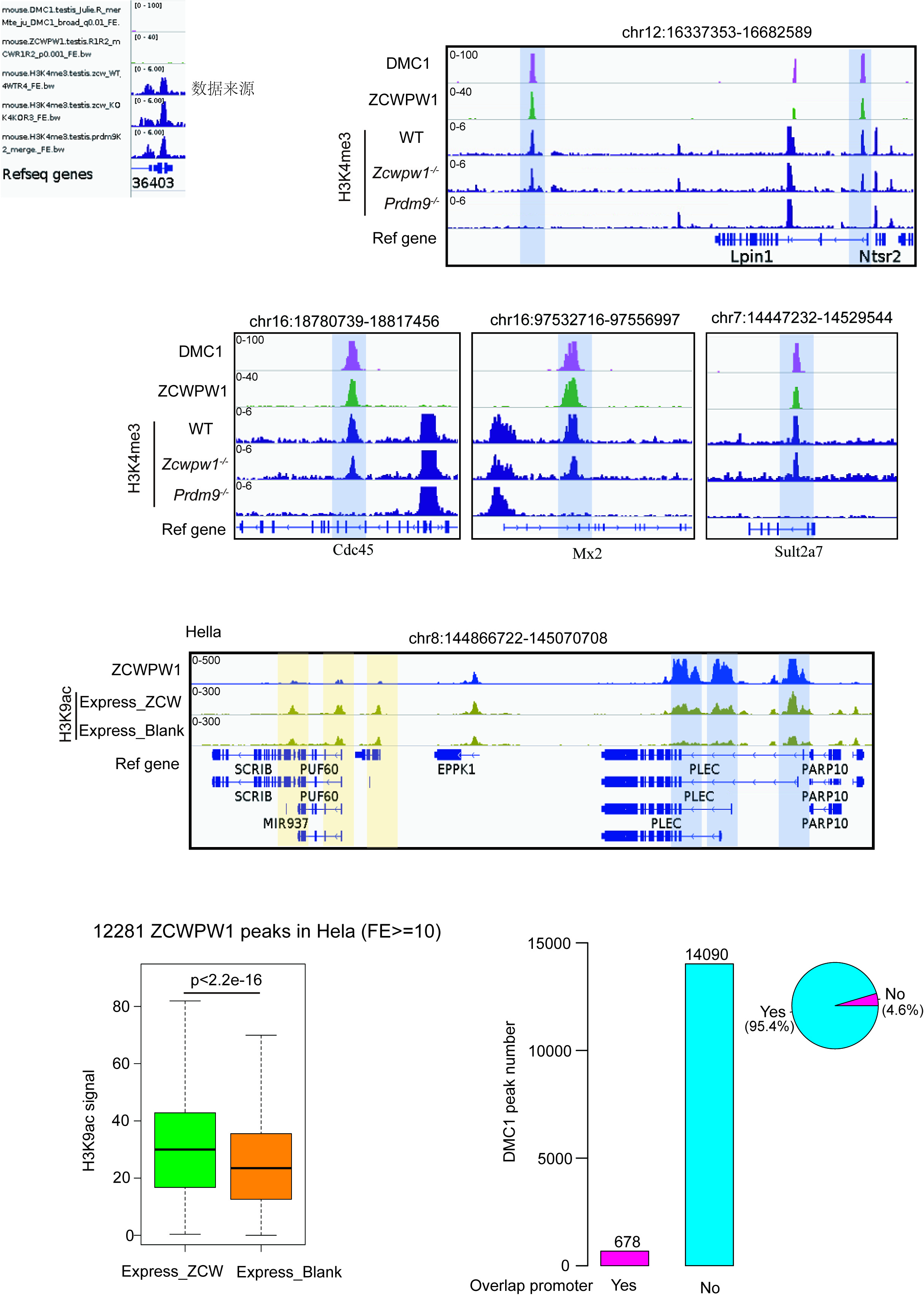
**

**Figure S8.** **H3K4me3 peaks at hotspot regions could be detected in *Zcwpw1^−/−^* testes**

Genome browser view of DMC1 and ZCWPW1 signals in WT mouse testes and the H3K4me3 signal in WT, *Prdm9^−/−^* and *Zcwpw1^−/−^* testes.

**
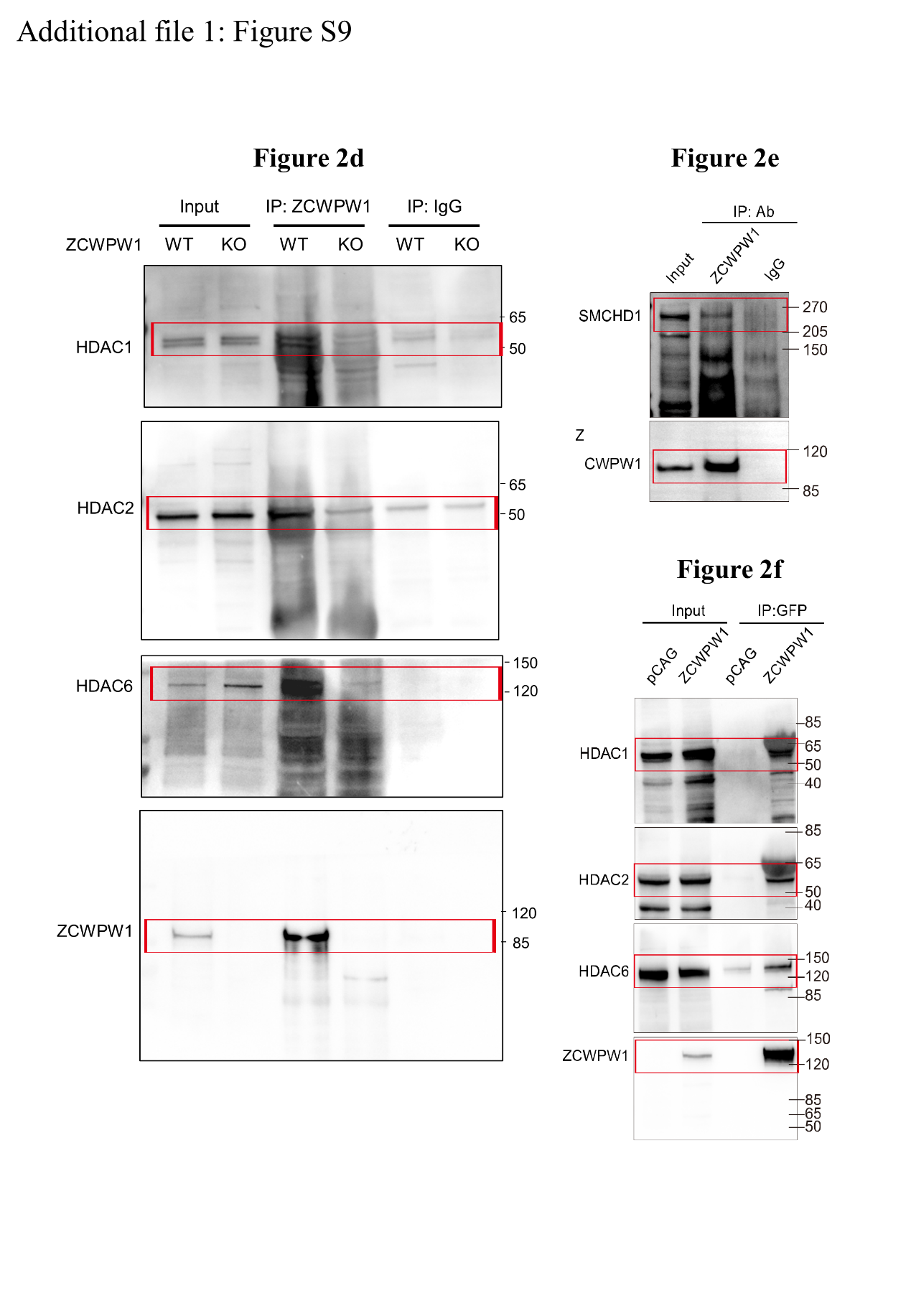
**

**Figure S9. Uncropped western blot gel images in Figure 2d, 2e and 2f.** Red boxes highlight lanes used in figures.


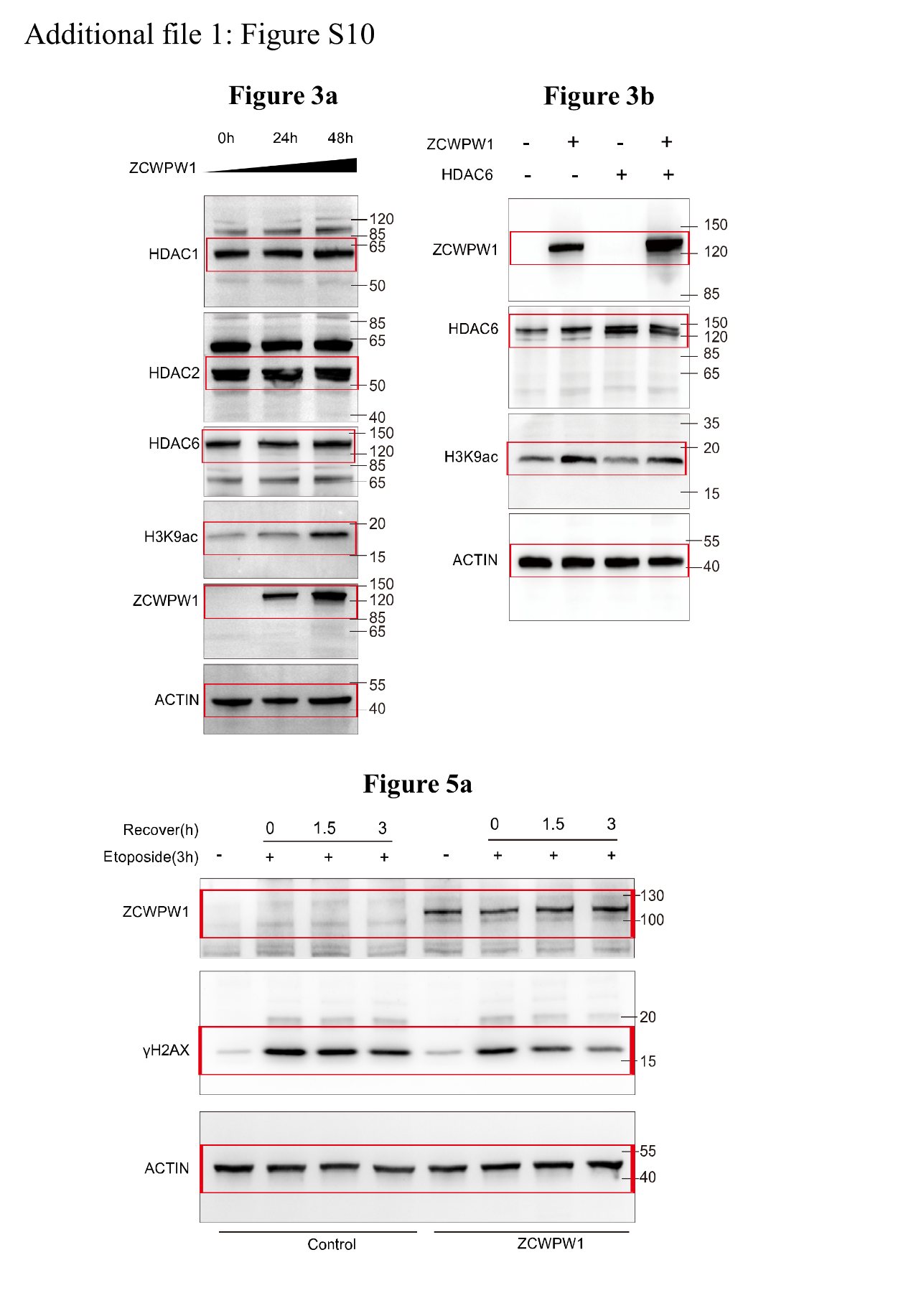


**Figure S10. Uncropped western blot gel images in Figure 3a, 3b and 5a.** Red boxes highlight lanes used in figures.


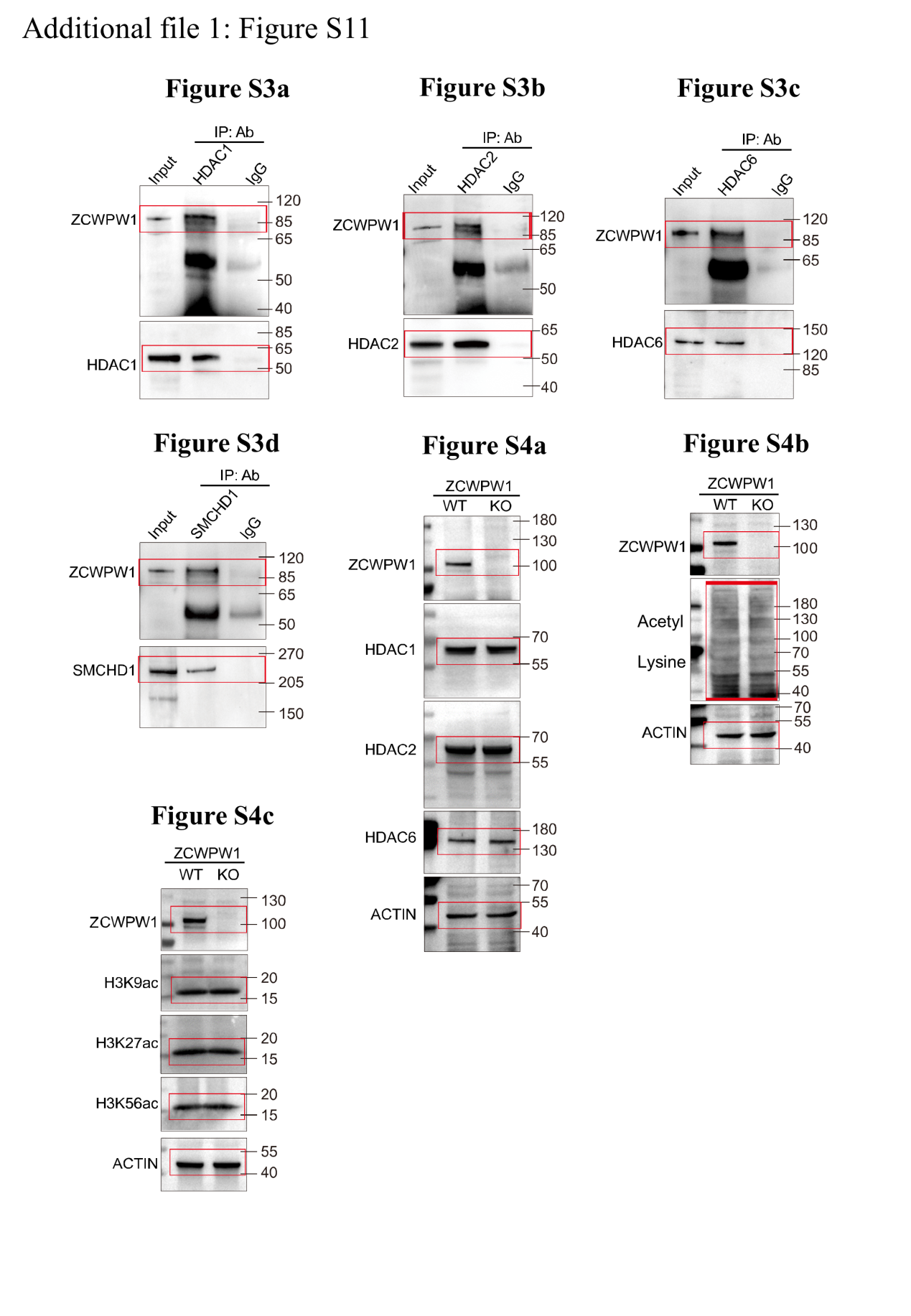


**Figure S11. Uncropped western blot gel images in Figure S3a-S3d and S4a-S4c.** Red boxes highlight lanes used in figures.


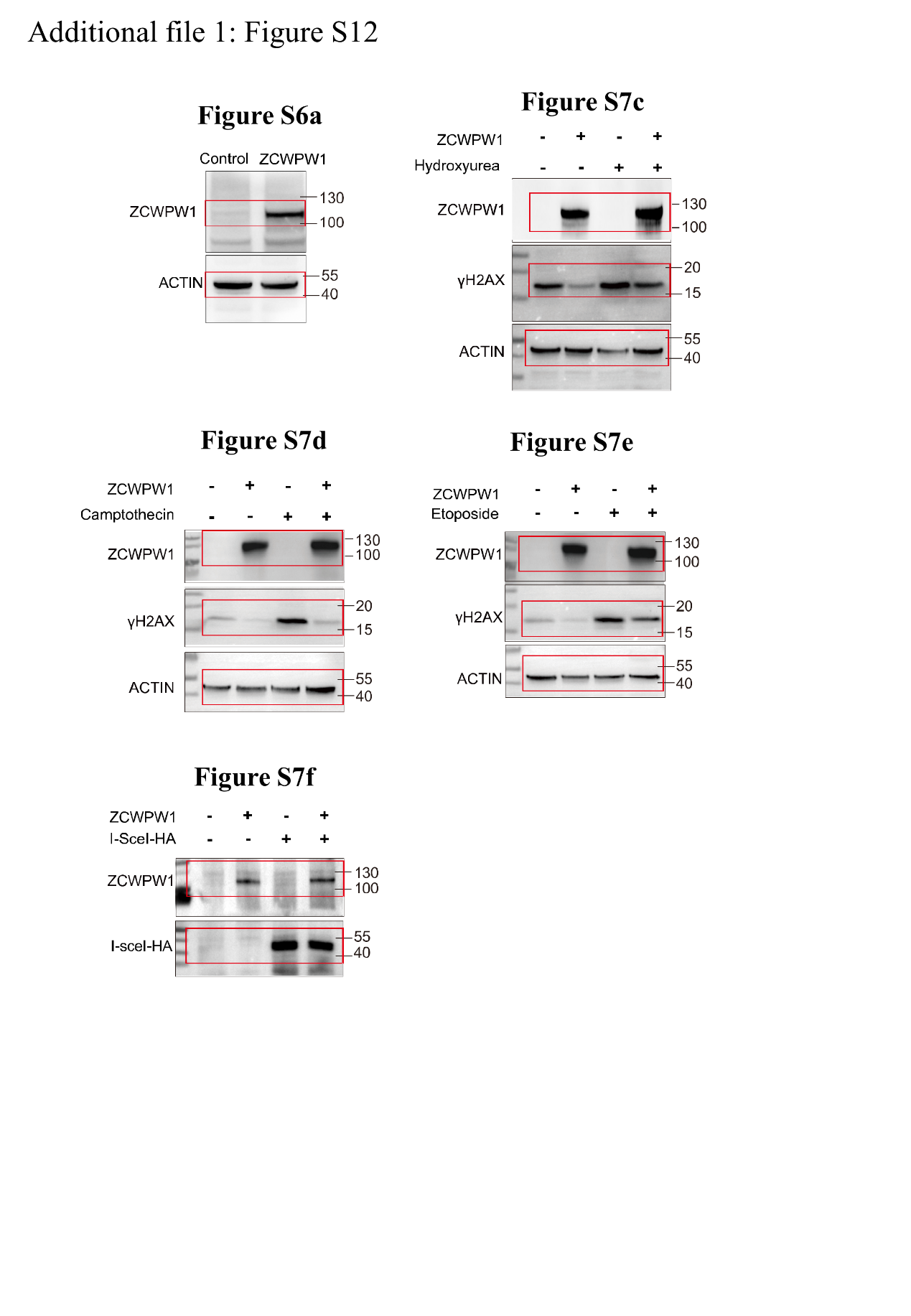


**Figure S12. Uncropped western blot gel images in Figure S6a and S7c-S7f.** Red boxes highlight lanes used in figures.
