# Supplementary material for: The histone modification reader ZCWPW1 promotes double-strand break repair by regulating cross-talk of histone modifications and chromatin accessibility at meiotic hotspots
Source: Genome Biol. 2022 Sep 6;23:187. doi: 10.1186/s13059-022-02758-z (PMC9446545; doi:10.1186/s13059-022-02758-z)
Supplement: Supplementary file 4 — Additional file 4. Review History. [file 13059_2022_2758_MOESM4_ESM.docx]

Review History

**First round of review**

**Reviewer 1**

**Were you able to assess all statistics in the manuscript, including the appropriateness of statistical tests used?**

N/A

**Comments to author:**

In the paper by Yuan et al. the authors address the potential mechanism by which the histone methylation reader ZCWPW1 facilitates homologous recombination during meiosis. Previous studies by this group and others have shown that this protein is necessary for completing homologous recombination and meiosis. Meiotic recombination in mammals is initiated at preferred sites, called hotspots (HS), where the histone methyltransferase PRDM9 binds. It subsequently marks these sites with dual H3K4me3 and H3K4me36. ZCWPW1 recognizes this dual mark but how this is coupled with DNA repair is unclear.

Here the authors find that in the absence of the histone methylation reader ZCWPW1, another histone modification, H3K9ac, that was previously found enriched at HS, is missing. They and others show that in wildtype mice the acetylation mark is independent of DSB formation but dependent on PRDM9 methyltransferase activity. This indicates that the H3K9ac mark is introduced after PRDM9 dependent modifications and its maintenance/introduction requires ZCWPW1. IP experiments suggest that ZCWPW1 interacts with histone deacetylases indicating that it might be counteracting deacetylation of H3K9ac at HS. ATAC-seq experiments in the ZCWPW1 show that the absence of H3K9ac correlates with a lack of chromatin openness. The authors then tried to assess whether ZCWPW1 could improve HR in somatic cells.

In summary, this is an important finding that provides a step forward in our understanding of the epigenetic mechanisms controlling meiotic recombination. The experiments are well conducted and the data supports the conclusions but the paper suffers from some lack of depth and details. Whereas the main finding (lack of H3K9ac in ZCWPW1 ko mice) is clear, the data supporting the mechanism is too preliminary and some controls are missing. The authors should provide more evidence or nuance their conclusions.

Major points:

1) Figure 1: It is unclear where the set of ZCWPW1 binding sites are from. Are these detected from experiments done by the authors or is published data (Mahgoub et al.). How are the group II peaks defined? Based on Mahgoub findings I suspect that they are coincidental/background signals found at promoters. This has implications for further analyses, and would explain why H3K9ac remains unchanged. The authors show data on group II throughout the manuscript but make no effort on trying to explain to the reader what might be going on there. Even from the heatmaps you can notice that the signals (ZCWPW1, DMC1, all of them) are different. The authors note that these are "weak" but this aspect is largely unexplored.

2) In figure 3, there is again a split between group I and group II but the numbers are different. Why is that? Are these split based on different criteria? What is the criteria? Here again, the fact that group II remain "open" supports the notion that they are promoters./

3) By RIME and Co-IP, the authors showed that "ZCWPW1 physically interacts with multiple HDAC proteins". However, the IgG control in the Co-IP picture (Figure 2D) shows clear bands, although they are weaker than the target bands. Did the author perform Co-IP with the ZCWPW1 KO mouse? Also, did the authors perform RIME in KO cells?

4) Figure 2E,F. The overexpression experiment in Hela cells provides a good indication for ZCWPW1 acting to counter histone deacetylases. But it is very unclear how this might happen in somatic cells. Whether the overexpressed ZCWPW1 interacts with HDACs in Hela cells is not shown in the manuscript. It is unclear whether the decreased deacetylation activity is because of the ZCWPW1/ HDACs interaction, or any other changes caused by overexpression of ZCWPW1.

Given that H3K9ac at the Group II peaks (likely promoters) doesn't change in mouse testes, how do the authors envision that ZCWPW1 affect deacetylation in Hela cells? Does it require a dual H3K4me3/K36 mark? If overexpression of ZCWPW1 can globally change the activity of HDACs by protein-protein interaction, ZCWPW1 could also modulate H3K9ac at non-hotspot sites in testis. This is important to elaborate because this is a key experiment to support the proposed mechanism. Also, did the authors assess the total acetylation in ZCWPW1 KO mice? I realize this might be challenging due to the perturbed cellularity compared to controls, but it might be interesting.

5) PRDM9 dependent H3K4me3 can be seen peaking at zygotene in meiotic spreads. Have you stained spread with H3K9Ac in wt and KO?

6) Figure 4: It is unclear that the overexpression of ZCWPW1 is aiding DNA repair. It would strengthen the paper if the authors could show time points at which γH2AX is the same in both samples (upon DSB induction) and it gets removed faster. As shown it cannot be ruled out that DNA damage is different in both conditions.

7) A recent manuscript (https://doi.org/10.1101/2022.03.15.484540) showed that dual histone methyl marks introduced by PRDM9 may impact the choice of DNA repair pathway. Here there are no histone methylation changes, what may be the possible mechanism that promotes HR in the cell line with ectopic expression of ZCWPW1? Just more "openness"?

8) ZCWPW1 KO mice phenotype varies between males and females. It would be interesting to add this point to the discussion.

===

This box is for your report. All comments included here will be passed to the authors

**Reviewer 2**

**Were you able to assess all statistics in the manuscript, including the appropriateness of statistical tests used?**

Yes

**Comments to author:**

Yuan et al. present a manuscript further exploring the epigenetic regulation of recombination hotspots in male mice, with an emphasis on the role of the epigenetic reader ZCWPW1 on hotspot H2K9ac modification and chromatin accessibility. Overall this is a very timely and interesting set of observations with a relatively newly discovered meiosis-specific epigenetic reader that has gained some attention in the last year or two. This group is one of the original groups to identify ZCWPW1's role in double-strand break repair, although the exact mechanism of action of ZCWPW1 is still not understood. Further, several groups have reported on the fact that recombination hotspots are marked by multiple histone modifications, including the relatively understudied (in meiosis) H3K9ac mark. The authors provide an interesting model whereby ZCWPW1 is recruited to hotspots via PRDM9-dependent modification at hotspots, where it promotes the active histone mark H3K9ac through inhibition of histone deacetylase enzymes and thus is proposed to facilitate DSB repair.

Overall, I am positive about the manuscript. I do have suggested clarifications to the text, would like to see some further development of the Discussion, and a couple issues around data presentation and support for the proposed model.

Comments

The title might be a little misleading. The authors do not formally or biochemically show 'interplay between chromatin marks'. While they do show that H3K9ac deposition or retention at hotspots is dependent on ZCWPW1, I assume the interplay comes from the fact that ZCWPW1 has domains that bind H3K4me3 and H3K36me3, which are deposited by PRDM9. So, the thought is that ZCWPW1 acts as a link between these 3 histone modifications. However, that is not at all clear from the title, and it still isn't fully understood, how that is important for chromatin accessibility. More on this below.

Abstract. Line 40. 'which provides a sufficiently long window for …DSB repair.' This is speculation and should be stated as a hypothesis or the authors predicted model of how the system works. This was not formally shown in the manuscript. Perhaps just include the word 'suggests'.

Results line 128. "Here, we speculate that the histone methylation reader ZCWPW1 may somehow mediate a relationship between H3K4me3 and H3K9ac." This proposition is presented with little justification. Is there any reason to assume an epigenetic binding protein such as ZCWPW1 would regulate deposition of another histone modification? I understand the proposed link between P9-dependent K4me3 and K9ac. Seems like an unnecessary post-hoc justification.

Results line 132. "ZCWPW1 binding sites were enriched with H3K9ac." Given what is known already about H3K9ac at hotspots, and that ZCWPW1 binds at hotspots dependent on PRDM9, is this surprising result? Seems expected.

Results line 136. "allowing the elimination of H3K9ac originating from cells that did not express ZCWPW1". This statement is confusing and/or inconsistent with the model presented at the beginning of the sentence. I understand the argument for higher overlap of K9ac in sorted cells with ZCWPW1. But K9ac is only deposited at hotspots depending on P9. So bulk ChIP-seq would only provide signal for cells that express P9 (ie leptotene/zygotene) so there would be no 'elimination of H3K9ac' from other cells because it wouldn't be a hotspot locations to begin with.

Results line 153. "indicating that such H3K9ac was independent of DSBs." Shown previously (Spruce et al. 2020).

Results line 183. "The H3K9ac signal was significantly increased…" and "no difference in the levels of HDAC1/2/6 was detected…". These two observations are not well-supported. First, the use of 'significant' suggests replication and statistical testing, which is not provided, therefore should not be used in this context. The n = 1 western blot suggests that H3K9ac is increased but quantification is not provided. Further, HDAC1 and HDAC6 by the very blot provided in Figure 2E look to be increased at least as much as H3K9ac. Yet is claimed in the text is otherwise. Again, replication nor quantification is lacking for these observations. This is a major concern around these results and need to be addressed if the claim is made in the manuscript.

Results sections pertaining to Figures 1B and 3B. It isn't immediately obvious from the text that there are two different Group I and Group II sets of ZCWPW1 peaks between these different analyses. I believe that the differences in these groups are somewhat compared in Supplemental Fig. 4B. Why not just use Group designation from Figure 1 for Figure 3 and then discuss similarities and differences?

Results ATAC-seq. Generally, several manuscripts have now shown that recombination hotspots in male meiosis are accessible via ATAC-seq and DNAse hypersensitivity, and that this is dependent on PRDM9, and occurs in the absence of SPO11 (Li et al. Cell Research 28(11). 2018, Spruce et al. G&D. 2020, Yamada et al. Genes&Development. 34. 2020). These should be referenced when results in the wild-type strain are presented here. However, a novel, and interesting, observation is that chromatin accessibility is lost in ZCWPW1 null, which I would like to see the implications of discussed further.

The lack of open chromatin at hotspots in the absence of ZCWPW1 is surprising. Several reports showed that in the absence of ZCWPW1, DSBs still occur at PRDM9-dependent hotspots, suggesting that the SPO11 machinery still has access to these sites to induce DSBs. How do the authors suggest SPO11 dependent DSBs occur at PRDM9-dependent hotspots in the absence of open chromatin? One assumes that in the absence of ZCWPW1 PRDM9, H3K4me3, and H3K36me3 persist at PRDM9-dependent hotspots, which is why DMC1 signal still occurs at those locations. This would fit the model of PRDM9 -> H3K4me3 -> ZCWPW1 -> H3K9ac -> repair. However, has the presence of H3K4me3 at hotspots been shown in ZCWPW1 mutant? This last question seems critical to the model presented here. If H3K4me3 is lacking in ZCWPW1 mutant (an experiment I couldn't find a result for if it was done here or previously), then it is not at all surprising that H3K9ac and ATAC-seq signal is also lacking. This would refute the authors model and potentially suggest different roles for ZCWPW1 in meiosis.

Potential Discussion topics:

Could you shed more light on the activities of the three HDACs? Do they target the identical residues on histones?

The observation that ectopic expression of ZCWPW1 ex vivo increased homologous recombination in a reporter system is interesting. How do the authors think that works? Based on the model from meiotic recombination, ZCWPW1 is recruited to hotspots via histone modifications deposited by P9. But these would be absent in the ex vivo Hela and U2OS systems. How would ZCWPW1 be recruited to sites of DSBs?

Minor Comments

The set of first experiment in the results section referred to the Supplementary figure 1. Is it typical, editorially, to start the manuscript with supplemental data? Why not include the ZCWPW1 ChIP results in Spo11 null mice in the main figure? Incidentally, it might be mentioned that the same result (presence of binding or modification in Spo11 null), has also been observed for H3K4me3, H3K9ac, and chromatin accessibility, all suggesting they precede DSBs.

Please include molecular weights positions based on markers in all Western blot figures, including Fig 2D, 2E. 4A, 4B. Further, full length images of Western blots (not cropped) either in supplement or supporting figure resource is highly recommended.

Please include further detail about filtering of RIME data. Bioinformatic analysis steps are included for -seq based analysis. What cutoffs were used to determine the final inclusion/exclusion list of 177 interacting proteins. Was PRDM9 identified?

Figure 4D. What is the n of this experiment? Please show individual data points on bar graphs.

Please include reference (Imai et al. Elife. Oct. 2020) for HELLS in the introduction

**Reviewer 3**

**Were you able to assess all statistics in the manuscript, including the appropriateness of statistical tests used?**

N/A

**Comments to author:**

022 ZCWPW1 paper

Overview:

Meiotic recombination in mammals is modulated by the histone methylase PRDM9 and the reader ZCWPW1, but precisely how these factors work mechanistically, is unclear. Here the authors report genome-wide analysis of chromatin marks (ChIP-seq) and chromatin accessibility (using ATAC-seq) in meiotic cells comparing controls to meiocytes lacking PRDM9, ZCWPW1 and SPO11 expression. Overall, the findings are quite interesting if a little provisional. The major observation of interest is that H3K9Ac mark is dependent on both the writer (PRDM9) and the reader (ZCWPW1) activity.

A number of issues are outstanding. For example, it is unclear ambiguous which datasets are new and which are reanalysis of published work. The authors would do well to make this much clearer in any revised manuscript. Secondly, the description of data analysis and its interpretation often lacks clarity, and may be often be over stated—but I am sure this can be addressed. Finally, the ectopic expression of ZCWPW1 in HeLa cells is potentially interesting, but appears to lack important controls concerning cell cycle distribution (again, something that should be quite easy to determine, but may alter the author's interpretations of their observations).

Specific comments:

Fig 1B. Please indicate in these and any other heatmaps by which parameter the data are ordered vertically.

Line 131: For comparison, what is the expected (random) overlap of ZCWPW1 and H3K9Ac in a null hypothesis that there is no relationship?

Line 139 and 142. Unless I am mistaken it is important to make it clear that Group 1 and Group 2 are DEFINED by their loss/retention of H3K9Ac in the absence of ZCWPW1. This is currently not clear.

Line 144. The term "significantly" is used, yet I don't read any use of statistical tests. Eitehr add a statistical test or revise the text to avoid use of significant, and state, "greatly", or some other adjective that describes the data.

Section 158-167. Is there a negative control for the Mass Spec analysis? The three replicates appear very different, with only minimal overlap. Are the HDACs known non-specific contaminants? It would help if these concerns were addressed in the text.

Line 183, 186. Again, "significant" is used. By what measure? Was this quantitative and reproducible? How large is the effect? Please avoid the potential for overstating the observations. My reading of the data presented is that the effect on H3K9Ac of overexpressing ZCWPW1 is, at best, modest.

Is there a reference for the assay used in Fig 1F? If not, please describe in more detail.

Line 198: Where are the data that show the number of open sites out of the total sites analysed? Please describe clearly. What were the criteria for a site to be considered "open"?

Line 199: The text is ambiguous. Is it 67% of group 1 sites? Or 67% of all sites? Are these group 1 and group 2 sites the same as those defined in Fig 1? If not, please explain how they were defined - by what criteria/threshold etc.

As above, please specify quantitative criteria that is used to decide "open-ness". Is this an arbitrary cut off? Please explain. The data look convincing, but the data description is lacking in clarity.

Line 202: Same issues as the points above. Please clarify.

Line 213. Which ZCWPW1 binding sites does "group 1" refer to? Those in Fig 1 (4886 sites) or in Fig 3 (2676 sites) ? It appears that these are different definitions, but that is not explained and appears confusing (to this reviewer…).

CRITICAL: Line 226. Please specify which agents are being. Fig S5 only shows Hydroxyurea.

CRITICAL: Given that HU and CPT are replication inhibitor/poison (and etoposide is also important for cycling cells): Did ZCWPW1 expression affect the proportion of cells in S-phase (independently of HU treatment)? Or the length of the cell cycle/doubling time? This is an important control/test to report for this entire section of work (Fig 4).

**Authors Response**

**Point-by-point responses to the reviewers’ comments:**

**Reviewer #1:** In the paper by Yuan et al. the authors address the potential mechanism by which the histone methylation reader ZCWPW1 facilitates homologous recombination during meiosis. Previous studies by this group and others have shown that this protein is necessary for completing homologous recombination and meiosis. Meiotic recombination in mammals is initiated at preferred sites, called hotspots (HS), where the histone methyltransferase PRDM9 binds. It subsequently marks these sites with dual H3K4me3 and H3K4me36. ZCWPW1 recognizes this dual mark but how this is coupled with DNA repair is unclear.

Here the authors find that in the absence of the histone methylation reader ZCWPW1, another histone modification, H3K9ac, that was previously found enriched at HS, is missing. They and others show that in wildtype mice the acetylation mark is independent of DSB formation but dependent on PRDM9 methyltransferase activity. This indicates that the H3K9ac mark is introduced after PRDM9 dependent modifications and its maintenance/introduction requires ZCWPW1. IP experiments suggest that ZCWPW1 interacts with histone deacetylases indicating that it might be counteracting deacetylation of H3K9ac at HS. ATAC-seq experiments in the ZCWPW1 show that the absence of H3K9ac correlates with a lack of chromatin openness. The authors then tried to assess whether ZCWPW1 could improve HR in somatic cells.

In summary, this is an important finding that provides a step forward in our understanding of the epigenetic mechanisms controlling meiotic recombination. The experiments are well conducted and the data supports the conclusions but the paper suffers from some lack of depth and details. Whereas the main finding (lack of H3K9ac in ZCWPW1 ko mice) is clear, the data supporting the mechanism is too preliminary and some controls are missing. The authors should provide more evidence or nuance their conclusions.

***Authors’ response:*** *We greatly appreciate this positive appraisal of our work and the encouraging comments. We have now performed new experiments and analyses following this guidance, and we trust the reviewer will agree that these have substantially improved our overall study and manuscript.*

Major points:

**1. Comments:**

Figure 1: It is unclear where the set of ZCWPW1 binding sites are from. Are these detected from experiments done by the authors or is published data (Mahgoub et al.). How are the group II peaks defined? Based on Mahgoub findings I suspect that they are coincidental/background signals found at promoters. This has implications for further analyses, and would explain why H3K9ac remains unchanged. The authors show data on group II throughout the manuscript but make no effort on trying to explain to the reader what might be going on there. Even from the heatmaps you can notice that the signals (ZCWPW1, DMC1, all of them) are different. The authors note that these are "weak" but this aspect is largely unexplored.

***Authors’ response:*** *We apologize for this confusion; please note that we have carefully ensured that the data sources for all of the datasets examined in the present study are now clearly explicated in the revised manuscript. Specifically, regarding this comment, the ZCWPW1 binding sites in wild type testes are from our previously published study(Huang et al., 2020), and the ZCWPW1 binding sites in Spo11^−/−^ testes are from our ChIP-seq analysis conducted in the present study. We have complemented detailed dataset information in the revised Data and Code Availability. To make it clear, we have summarized this information in the revised manuscript in supplementary table 2.*

*We totally agree with the reviewer’s point. We defined group I as those ZCWPW1 binding sites that lost H3K9ac peaks in Zcwpw1^−/−^ testes. Group II represents those ZCWPW1 binding sites that retained H3K9ac peaks in Zcwpw1^−/−^ testes. We found that 73.4% of the Group II ZCWPW1 binding sites are located at transcript promoter regions, while only 4.1% of Group I ZCWPW1 binding sites are located at promoter regions (Fig. S2D). Zcwpw1 and Prdm9 are co-expressed in spermatocytes during meiosis(Mahgoub et al., 2020). The cells co-expressing Zcwpw1 and Prdm9 (mainly leptotene and zygotene spermatocytes) occupy a small proportion in whole testes(Bellve et al., 1977; Mahgoub et al., 2020). The H3K9ac peaks are enriched at promoter regions in both the cells expressing ZCWPW1 and those not expressing ZCWPW1. In Zcwpw1^-/-^ whole testes, even though the H3K9ac peaks at promoter regions overlapping ZCWPW1 peaks were lost in the cells expressing ZCWPW1, these same H3K9ac peaks could also be detected in cells not expressing ZCWPW1. Indeed, the intensity of Group II H3K9ac peaks was slightly reduced in Zcwpw1^−/−^* *and Prdm9^−/−^ whole testes (Fig. 1B).*

*The histone methyltransferase PRDM9 defines the meiotic recombination sites, called hotspots, in mammals, and most of the hotspots are located at non-promoter regions(Baudat et al., 2013). Previously, three groups independently reported that ZCWPW1 recognizes dual marks deposited by PRDM9, and only 11.4% (1674/14688) of ZCWPW1 peaks were enriched at promoter regions(Huang et al., 2020; Mahgoub et al., 2020; Wells et al., 2020). Khil and colleagues developed an SSDS analysis to detect DMC1-bound single-stranded DNA at DSB ends, and only 4.6% (678/14768) of DMC1 peaks were enriched at promoter regions (Fig. R1A and R1B)(Grey et al., 2017; Khil et al., 2012). These results indicate that most of DSBs are induced at non-promoter recombination hotspots. Given that 73.4% of the Group II ZCWPW1 binding sites are located at promoter regions, the differences of ZCWPW1 and DMC1 signals between Group I and Group II are reasonable.*

*Figure R1. (A) Bar plot to show the number of DMC1 peaks at promoter regions (TSS ±2kb). (B) Pie chart to show the proportion of DMC1 peaks at promoter regions (TSS ±2kb)*.

**2. Comments:**

In figure 3, there is again a split between group I and group II but the numbers are different. Why is that? Are these splits based on different criteria? What are the criteria? Here again, the fact that group II remain "open" supports the notion that they are promoters.

***Authors’ response:*** *We apologize for the confusion. We have detected 6627 H3K9ac peaks and 4015 ATAC signals overlapping ZCWPW1 peaks in whole testes. Here to make it clear, we rename Group H1 and Group H2 in figure1 and Group A1 and Group A2 in figure3. Group H1 represents those ZCWPW1 binding sites that lost H3K9ac peaks in Zcwpw1^−/−^ testes; Group H2 represents those ZCWPW1 binding sites that retained H3K9ac peaks in Zcwpw1^−/−^ testes (Fig. 1B). Group A1 indicates ZCWPW1 binding sites that lost ATAC signal in Zcwpw1^−/−^ testes, while Group A2 indicates ZCWPW1 binding sites that retained ATAC signal in Zcwpw1^−/−^ testes (Fig. 4B). We found that 61.3% of Group A2 ZCWPW1 binding sites are located at transcript promoter regions, while only 4.7% of Group A1 ZCWPW1 binding sites are located at promoter regions (Fig. S5B), which indicated that Group A2 remains "open" and supports the notion that they are promoters.*

**3. Comments:**

By RIME and Co-IP, the authors showed that "ZCWPW1 physically interacts with multiple HDAC proteins". However, the IgG control in the Co-IP picture (Figure 2D) shows clear bands, although they are weaker than the target bands. Did the author perform Co-IP with the ZCWPW1 KO mouse? Also, did the authors perform RIME in KO cells?

***Authors’ response:*** *We thank the reviewer for raising this very important question. We have performed Co-IP between ZCWPW1 and HDACs in the wild type and Zcwpw1^−/−^ testes. We found that there are weaker target bands of HDACs in Zcwpw1^−/−^ testes in ZCWPW1 antibody and IgG antibody immunoprecipitation groups (Fig. R2), which indicated that these weaker target bands in the wild type testes IgG control might be non-specific bands. We did not perform RIME assay in Zcwpw1^−/−^ testes, the IgG RIME assay in wild type testes was used as negative control in this experiment.*

*Figure R2. Co-immunoprecipitation analysis of ZCWPW1 binding proteins from PD14-PD16 testes protein extracts. HDAC1, HDAC2, HDAC6 were immunoprecipitated with ZCWPW1, Zcwpw1^−/−^ mice testes protein extracts and IgG antibody as negative controls.*

**4. Comments:**

Figure 2E, F. The overexpression experiment in Hela cells provides a good indication for ZCWPW1 acting to counter histone deacetylases. But it is very unclear how this might happen in somatic cells. Whether the overexpressed ZCWPW1 interacts with HDACs in Hela cells is not shown in the manuscript. It is unclear whether the decreased deacetylation activity is because of the ZCWPW1/ HDACs interaction, or any other changes caused by overexpression of ZCWPW1.

Given that H3K9ac at the Group II peaks (likely promoters) doesn't change in mouse testes, how do the authors envision that ZCWPW1 affect deacetylation in Hela cells? Does it require a dual H3K4me3/K36 mark? If overexpression of ZCWPW1 can globally change the activity of HDACs by protein-protein interaction, ZCWPW1 could also modulate H3K9ac at non-hotspot sites in testis. This is important to elaborate because this is a key experiment to support the proposed mechanism. Also, did the authors assess the total acetylation in ZCWPW1 KO mice? I realize this might be challenging due to the perturbed cellularity compared to controls, but it might be interesting.

***Authors’ response:*** *We appreciate the suggestions from the reviewer. We have now performed Co-IP of ZCWPW1 and HDACs in HeLa cells overexpressing ZCWPW1. Our results showed that ZCWPW1 also interacted with HDACs in HeLa cells (Fig. 2E).*

*ZCWPW1 only recognizes dual histone methylation marks deposited by PRDM9 at hotspots in testes(Huang et al., 2020; Mahgoub et al., 2020; Wells et al., 2020). In Zcwpw1^−/−^ testes, 74% (4886/6627) (Group H1) of the ZCWPW1 binding sites lost H3K9ac signal in Zcwpw1^−/−^ mouse testes (Fig. 1A and 1B), while the H3K9ac signal at all promoter regions was comparable between WT and Zcwpw1^−/−^ mouse testes (Fig. S2B). Wells et al reported enrichment for ZCWPW1 at hotspot sites and abundant non-hotspot sites including promoter regions (where enriched with either H3K4me3 or H3K36me3) in HEK293T cells co-overexpressing PRDM9 and ZCWPW1(Wells et al., 2020). When we expressed ZCWPW1 in HeLa cells, we found that the H3K9ac signal overlapping ZCWPW1 binding sites was significantly greater than that in HeLa cells expressing control plasmids (Fig. S4D and S4E). Thus, we suggest that the function of ZCWPW1’s preserving H3K9ac by antagonizing HDAC proteins’ deacetylation activity is dependent on its recognizing H3K4me3 and/or H3K36me3.*

*We have now performed* *immunoblotting of several HDACs, acetyl lysine and histone acetylation marks in WT and Zcwpw1^−/−^ testes at postnatal day 14, the protein level of the candidates was comparable between the two groups (Fig. S4A, S4B and S4C).*

**5. Comments:**

PRDM9 dependent H3K4me3 can be seen peaking at zygotene in meiotic spreads. Have you stained spread with H3K9Ac in WT and KO?

***Authors’ response:*** *In stage-specific ChIP-seq, Lam et al found that the dynamics of H3K9ac through MPI resemble those of H3K4me3: a signal is first observed at hotspots in leptonema, maximal at zygonema and absent by early pachynema(Lam et al., 2019). We also found that the immunofluorescence staining signal intensity of H3K4me3 could be seen peaking at zygotene stage in spermatocytes(Huang et al., 2020). We have now stained the spreads with H3K9ac in WT and knockout mice and found that the H3K9ac signal intensity was comparable from leptotene to zygotene stage between WT and knockout mice spermatocytes (Spo11^−/−^/Prdm9^−/−^/Zcwpw1^−/−^) (Fig. R3). Although H3K9ac peaks at hotspot regions are lost in Prdm9^−/−^ and Zcwpw1^−/−^ mice, considering that H3K9ac peaks at hotspot regions only occupy small proportion (12834/59420) of all H3K9ac peaks in leptotene and zygotene stage spermatocytes(Lam et al., 2019). Immunofluorescence staining could not detect the exact difference between WT and knockout spermatocytes, and this was a reasonable finding.*

*Figure R3. (A) Chromosome spreads of spermatocytes from the testes of adult WT, Spo11^−/−^, Prdm9^−/−^ and Zcwpw1^−/−^ males immunostained for H3K9ac (green) and SYCP3 (red). Representative images are shown for spermatocytes at the leptotene, zygotene, pachytene (circle indicates the XY body), and pachytene-like stages of the four genotypes.*

**6. Comments:**

Figure 4: It is unclear that the overexpression of ZCWPW1 is aiding DNA repair. It would strengthen the paper if the authors could show time points at which γH2AX is the same in both samples (upon DSB induction) and it gets removed faster. As shown, it cannot be ruled out that DNA damage is different in both conditions.

***Authors’ response:*** *We have now performed new experiments following reviewer’s suggestion, and this content now reads as follows:*

*“To evaluate the DNA damage repair capacity between control and ZCWPW1-overexpressing HeLa cells induced by etoposide (ETO), γH2AX levels were measured by western blot as an indicator of DSBs. After exposure to ETO for 3 h, γH2AX levels were greatly increased in both control and ZCWPW1-overexpressing HeLa cells (Fig. 5A and S6E). However, the clearing rate of γH2AX signals increased in HeLa cells expressing ZCWPW1 compared to Control (Fig. 5A and S6E), which suggested that ZCWPW1 overexpression promotes the progression of DSB repair in HeLa cells.”*

**7. Comments:**

A recent manuscript (https://doi.org/10.1101/2022.03.15.484540) showed that dual histone methyl marks introduced by PRDM9 may impact the choice of DNA repair pathway. Here there are no histone methylation changes, what may be the possible mechanism that promotes HR in the cell line with ectopic expression of ZCWPW1? Just more "openness"?

***Authors’ response:*** *Thanks for your comments. It has been reported that histone methylation such as H3K36me3 and H3K4me3 could be introduced at DSB sites in somatic cells(Bayley et al., 2022; Pfister et al., 2014). In addition, it is well-established that histone methylation, acetylation and chromatin accessibility could promote DSB repair through HR in somatic cells(Manickavinayaham et al., 2019; Ogiwara et al., 2011; Price and D'Andrea, 2013). Previous work has confirmed that ZCWPW1 could bind to a large number of genomic regions (such as promoters) enriched with either H3K4me3 or H3K36me3 in HEK293T cell lines when ZCWPW1 is ectopically expressed(Wells et al., 2020). In this study, we have confirmed that ZCWPW1 could maintain the H3K9ac level by antagonizing HDACs both in testes (Fig 1B) and cell lines (Fig. S4D and S4E), which may further impact the choice of DSB repair pathway. Kindly see the response for essential comment 4.*

**8. Comments:**

ZCWPW1 KO mice phenotype varies between males and females. It would be interesting to add this point to the discussion.

***Authors’ response:*** *Thanks for your comments. The sexual dimorphism has been found in many knockout mice, such as Sycp3^−/−^, Tex11^−/−^, Hspa2^−/−^ and Rad21l^−/−^(Adelman and Petrini, 2008; Dix et al., 1996; Herran et al., 2011; Yuan et al., 2000). We have discussed the sexual dimorphism between Zcwpw1 knockout males and females in our previous study(Li et al., 2019). Briefly, we found that ZCWPW1 accumulates at sex bodies in pachytene spermatocytes, indicating that ZCWPW1 might be involved in sex-body formation or even meiotic sex chromosome inactivation (MSCI) in an unknown way. MSCI functions as a surveillance system in spermatocytes but not in oocytes because oocytes have no Y chromosomes. This may be a key factor to explain the observed sexual dimorphism. The possible function of ZCWPW1 in the formation and regulation of sex bodies represents a major question in meiosis studies that deserves full investigation, which will be part of our next project.*

**Reviewer #2:** Yuan et al. present a manuscript further exploring the epigenetic regulation of recombination hotspots in male mice, with an emphasis on the role of the epigenetic reader ZCWPW1 on hotspot H3K9ac modification and chromatin accessibility. Overall, this is a very timely and interesting set of observations with a relatively newly discovered meiosis-specific epigenetic reader that has gained some attention in the last year or two. This group is one of the original groups to identify ZCWPW1's role in double-strand break repair, although the exact mechanism of action of ZCWPW1 is still not understood. Further, several groups have reported on the fact that recombination hotspots are marked by multiple histone modifications, including the relatively understudied (in meiosis) H3K9ac mark. The authors provide an interesting model whereby ZCWPW1 is recruited to hotspots via PRDM9-dependent modification at hotspots, where it promotes the active histone mark H3K9ac through inhibition of histone deacetylase enzymes and thus is proposed to facilitate DSB repair. Overall, I am positive about the manuscript. I do have suggested clarifications to the text, would like to see some further development of the Discussion, and a couple issues around data presentation and support for the proposed model.

***Authors’ response:*** *We greatly appreciate the positive appraisal of our work. We have now extensively modified our manuscript according to the reviewer’s suggestions.*

Comments

**1. Comments:**

The title might be a little misleading. The authors do not formally or biochemically show interplay between chromatin marks. While they do show that H3K9ac deposition or retention at hotspots is dependent on ZCWPW1, I assume the interplay comes from the fact that ZCWPW1 has domains that bind H3K4me3 and H3K36me3, which are deposited by PRDM9. So, the thought is that ZCWPW1 acts as a link between these 3 histone modifications. However, that is not at all clear from the title, and it still isn't fully understood, how that is important for chromatin accessibility. More on this below.

***Authors’ response:*** *We are very grateful for the Reviewer’s constructive comments. To make it clear, now the title has been revised as follows:*

*“The histone modification reader ZCWPW1 promotes double-strand break repair by regulating cross-talk of histone modifications and chromatin accessibility at meiotic hotspots.”*

**2. Comments:**

Abstract. Line 40. Which provides a sufficiently long window for …DSB repair. This is speculation and should be stated as a hypothesis or the authors predicted model of how the system works. This was not formally shown in the manuscript. Perhaps just include the word 'suggests'.

***Authors’ response:*** *We appreciate this suggestion from the reviewer. In the revised version, this content reads as:*

*“Here, we found that the histone methylation reader ZCWPW1 is essential for maintaining H3K9ac by antagonizing HDAC proteins’ deacetylation activity and further promotes chromatin openness at recombination hotspots thus, preparing the way for homologous recombination during meiotic double-strand break repair.”*

**3. Comments:**

Results line 128. "Here, we speculate that the histone methylation reader ZCWPW1 may somehow mediate a relationship between H3K4me3 and H3K9ac." This proposition is presented with little justification. Is there any reason to assume an epigenetic binding protein such as ZCWPW1 would regulate deposition of another histone modification? I understand the proposed link between P9-dependent K4me3 and K9ac. Seems like an unnecessary post-hoc justification.

***Authors’ response:*** *We agree with the reviewer’s point, and in the revised manuscript, this content reads as:*

*“However, the molecular mechanism for such cross-talk between histone modifications is poorly understood. Here, we hypothesized that the histone methylation reader ZCWPW1 mediates the cross-talk between H3K4me3 and H3K9ac.”*

**4. Comments:**

Results line 132. "ZCWPW1 binding sites were enriched with H3K9ac." Given what is known already about H3K9ac at hotspots, and that ZCWPW1 binds at hotspots dependent on PRDM9, is this surprising result? Seems expected.

***Authors’ response:*** *The overlapping between ZCWPW1 binding sites and DMC1 peaks was 75.7% (11124/14688), while the overlapping between DMC1 peaks and H3K9ac peaks was 86.9% (12834/14768)(Huang et al., 2020; Khil et al., 2012; Lam et al., 2019). Here we want to show how many ZCWPW1 binding sites were overlapping with H3K9ac peaks.*

**5. Comments:**

Results line 136. "Allowing the elimination of H3K9ac originating from cells that did not express ZCWPW1". This statement is confusing and/or inconsistent with the model presented at the beginning of the sentence. I understand the argument for higher overlap of K9ac in sorted cells with ZCWPW1. But K9ac is only deposited at hotspots depending on P9. So bulk ChIP-seq would only provide signal for cells that express P9 (ie leptotene/zygotene) so there would be no 'elimination of H3K9ac' from other cells because it wouldn't be a hotspot locations to begin with.

***Authors’ response:*** *In the H3K9ac ChIP-seq from whole testes, the H3K9ac signals are from leptotene and zygotene cells (LZ cells) and other cells (non-LZ cells). In the H3K9ac ChIP-seq from sorted cells (LZ cells), the H3K9ac signals are only from LZ cells. It is well known that most H3K9ac signal at hotspot sites only exist in LZ cells in the whole testes(Lam et al., 2019). However, meiotic progression is asynchronous in mammals and LZ cells only occupy small proportion in whole testes(Bellve et al., 1977). The H3K9ac signals from non-LZ cells in whole testes will dilute the proportion of H3K9ac signals from LZ cells at hotspot sites. Indeed, only 40.5% (5195/12834) H3K9ac peaks at hotspot sites were detected in whole testes compared with sorted LZ cells(Lam et al., 2019)*

**6. Comments:**

Results line 153. "Indicating that such H3K9ac was independent of DSBs." Shown previously (Spruce et al. 2020).

***Authors’ response:*** *Thanks for your comment. We have now added the reference in the re-drafted text, this content now reads as follows:*

*“However, we found that the H3K9ac signal was evident at Group H1 ZCWPW1 binding sites in Spo11^−/−^ mouse testes (Fig. 1A, 1B and S2E), indicating that the presence of H3K9ac was independent of DSBs, which was consistent with previous reports (Buard et al., 2009; Spruce et al., 2020).”*

**7. Comments:**

Results line 183. "The H3K9ac signal was significantly increased…" and "no difference in the levels of HDAC1/2/6 was detected…". These two observations are not well-supported. First, the use of 'significant' suggests replication and statistical testing, which is not provided, therefore should not be used in this context. The n = 1 western blot suggests that H3K9ac is increased but quantification is not provided. Further, HDAC1 and HDAC6 by the very blot provided in Figure s2E look to be increased at least as much as H3K9ac. Yet is claimed in the text is otherwise. Again, replication nor quantification is lacking for these observations. This is a major concern around these results and need to be addressed if the claim is made in the manuscript.

***Authors’ response:*** *We appreciate this directed emphasis on quantitative precision.*

*And now replication and quantification have been provided in Fig. 3A, 3B and 3C. In the re-drafted text, this content now reads as follows:*

*“Given that knock-out of Zcwpw1 could greatly reduce the H3K9ac signal at recombination hotspots (Fig. 1B) but did not affect the protein levels of the HDACs that ZCWPW1 interacts with (Fig. S4A), we hypothesized that ZCWPW1 antagonizes the deacetylation activity of HDACs to maintain the H3K9ac signal. To test this, we expressed ZCWPW1 in HeLa cells. The H3K9ac signal was significantly increased in HeLa cells expressing ZCWPW1 compared to control cells transfected with an empty-vector (Fig. 3A, 3B and 3C). Further, we expressed HDAC6 alone or with ZCWPW1 in HeLa cells, and the H3K9ac signal was slightly reduced in the HDAC6 overexpression group but was partly recovered in the ZCWPW1 and HDAC6 co-expression group (Fig. 3B). We also performed HDAC activity assays in HeLa cells, and as expected the deacetylation activities of HDACs were modestly decreased in cells expressing ZCWPW1 compared to controls (Fig. 3D). Thus, ZCWPW1’s interaction with HDAC enzymes restricts their deacetylation activities, at least partially explaining the elevated H3K9ac levels at recombination hotspots.”*

**8. Comments:**

Results sections pertaining to Figures 1B and 3B. It isn't immediately obvious from the text that there are two different Group I and Group II sets of ZCWPW1 peaks between these different analyses. I believe that the differences in these groups are somewhat compared in Supplemental Fig. 4B. Why not just use Group designation from Figure 1 for Figure 3 and then discuss similarities and differences?

***Authors’ response:*** *We thank the reviewer for this very interesting idea. We have detected 6627 H3K9ac peaks and 4015 ATAC signals overlapping ZCWPW1 peaks in whole testes. However, 74% (4886/6627) H3K9ac peaks and 67% (2676/4015) ATAC signals were lost in Zcwpw1^−/−^ testes. Here we attempted to investigate the relationship between H3K9ac peaks and ATAC signals with ZCWPW1 binding sites independently. To make it clear, we rename group H1 and group H2 in figure1 and group A1 and group A2 in figure3. Group H1 represents those ZCWPW1 binding sites that lost H3K9ac peaks in Zcwpw1^−/−^ testes; Group H2 represents those ZCWPW1 binding sites that retained H3K9ac peaks in Zcwpw1^−/−^ testes. Group A1 indicates ZCWPW1 binding sites that lost ATAC signal in Zcwpw1^−/−^ testes, while Group A2 indicates ZCWPW1 binding sites that retained ATAC signal in Zcwpw1^−/−^ testes.*

**9. Comments:**

Results ATAC-seq. Generally, several manuscripts have now shown that recombination hotspots in male meiosis are accessible via ATAC-seq and DNAse hypersensitivity, and that this is dependent on PRDM9, and occurs in the absence of SPO11 (Li et al. Cell Research 28(11). 2018, Spruce et al. G&D. 2020, Yamada et al. Genes&Development. 34. 2020). These should be referenced when results in the wild-type strain are presented here. However, a novel, and interesting, observation is that chromatin accessibility is lost in ZCWPW1 null, which I would like to see the implications of discussed further.

***Authors’ response:*** *Thanks for your comments. The related papers have been referenced when results in the wild-type strain are presented. The function of ZCWPW1 on H3K9ac and chromatin accessibility at hotspots has been discussed in the revised manuscript (Page 12-14, Discussion).*

**10. Comments:**

The lack of open chromatin at hotspots in the absence of ZCWPW1 is surprising. Several reports showed that in the absence of ZCWPW1, DSBs still occur at PRDM9-dependent hotspots, suggesting that the SPO11 machinery still has access to these sites to induce DSBs. How do the authors suggest SPO11 dependent DSBs occur at PRDM9-dependent hotspots in the absence of open chromatin? One assumes that in the absence of ZCWPW1 PRDM9, H3K4me3, and H3K36me3 persist at PRDM9-dependent hotspots, which is why DMC1 signal still occurs at those locations. This would fit the model of PRDM9 -> H3K4me3 -> ZCWPW1 -> H3K9ac -> repair. However, has the presence of H3K4me3 at hotspots been shown in ZCWPW1 mutant? This last question seems critical to the model presented here. If H3K4me3 is lacking in ZCWPW1 mutant (an experiment I couldn't find a result for if it was done here or previously), then it is not at all surprising that H3K9ac and ATAC-seq signal is also lacking. This would refute the authors model and potentially suggest different roles for ZCWPW1 in meiosis.

***Authors’ response:*** *We have now performed ChIP-seq assays of H3K4me3 in Zcwpw1^−/−^ testes, and found that the H3K4me3 peaks at hotspot regions retained in Zcwpw1^−/−^ testes (Fig. R4), while these same H3K4me3 peaks are lost in Prdm9^−/−^ testes. Recall that meiotic DNA double-strand break formation is dependent on PRDM9 methyltransferase activity, that is, the H3K4me3 and H3K36me3 are marks deposited by PRDM9(Diagouraga et al., 2018). DSBs still occur at PRDM9-dependent hotspots in Zcwpw1^−/−^ testes, suggesting that the SPO11 machinery still has access to these sites to induce DSBs(Mahgoub et al., 2020; Wells et al., 2020). So, these results support a working model wherein PRDM9 binds to hotspot sites and writes H3K4me3 and H3K36me3(Diagouraga et al., 2018; Powers et al., 2016). This leads to the recruitment of SPO11 machinery required for the formation of DSBs(Kumar et al., 2018; Panizza et al., 2011). After these PRDM9-catalyzed epigenetic modifications are deposited, ZCWPW1 can specifically read these H3K4me3 and H3K36me3 marks in the vicinity of DSB sites, where ZCWPW1 functions to maintain the H3K9ac and chromatin accessibility to promote DSB repair(Huang et al., 2020).*

*Figure R4. (A) Genome browser view of DMC1 and ZCWPW1 signals in WT mouse testes and the H3K4me3 signal in WT, Prdm9^−/−^ and Zcwpw1^−/−^ testes.*

Potential Discussion topics:

**11. Comments:**

Could you shed more light on the activities of the three HDACs? Do they target the identical residues on histones?

***Authors’ response:*** *It has been reported that HDAC1 and HDAC2 could target H3K9ac by biochemical assay(Wagner et al., 2015; Wang et al., 2020). Moreover, we found that the H3K9ac signal was modestly reduced in HeLa cells expressing HDAC6 compared to empty-vector-transfected control cells (Fig. 3B). However, whether HDAC6 can target H3K9ac needs further confirmation.*

**12. Comments:**

The observation that ectopic expression of ZCWPW1 ex vivo increased homologous recombination in a reporter system is interesting. How do the authors think that works? Based on the model from meiotic recombination, ZCWPW1 is recruited to hotspots via histone modifications deposited by P9. But these would be absent in the ex vivo Hela and U2OS systems. How would ZCWPW1 be recruited to sites of DSBs?

***Authors’ response:*** *Thanks for the comments. It has been reported that histone methylation such as H3K36me3 and H3K4me3 could be introduced at DSB sites in somatic cells(Bayley et al., 2022; Pfister et al., 2014). In addition, it is well-established that histone methylation, acetylation and chromatin accessibility could promote DSB repair through HR in somatic cells(Manickavinayaham et al., 2019; Ogiwara et al., 2011; Price and D'Andrea, 2013). Previous work has confirmed that ZCWPW1 could bind to a large number of genomic regions (such as promoters) enriched with either H3K4me3 or H3K36me3 in HEK293T cell lines when ZCWPW1 is ectopically expressed(Wells et al., 2020). When we expressed ZCWPW1 in HeLa cells, we found that the H3K9ac signal overlapping ZCWPW1 binding sites was significantly greater than that in HeLa cells expressing control plasmids (Fig. S4D and S4E). Here, considering the remarkably different context between the testes and cell lines, we speculate that ZCWPW1 might bind to genomic regions with either H3K4me3 or H3K36me3 in cell lines by its CW or PWWP domain function. Kindly see the response to Reviewer 1 essential comment 4.*

Minor Comments

**13. Comments:**

The set of first experiment in the results section referred to the Supplementary figure 1. Is it typical, editorially, to start the manuscript with supplemental data? Why not include the ZCWPW1 ChIP results in Spo11 null mice in the main figure? Incidentally, it might be mentioned that the same result (presence of binding or modification in Spo11 null), has also been observed for H3K4me3, H3K9ac, and chromatin accessibility, all suggesting they precede DSBs.

***Authors’ response:*** *Thanks for your suggestions. Recall that ZCWPW1 is an H3K4me3/H3K36me3 reader, and its binding at recombination hotspots is strongly dependent on the histone modification activity of PRDM9 and also its CW domain’s reader function(Huang et al., 2020). The H3K4me3, H3K9ac and chromatin accessibility at hotspots have been reported that they all precede DSBs(Buard et al., 2009; Chen et al., 2020; Imai et al., 2020; Spruce et al., 2020). So, it is not surprising that the germ cell specific histone methylation reader ZCWPW1 peaks are retained in Spo11 null mice.*

**14. Comments:**

Please include molecular weights positions based on markers in all Western blot figures, including Fig 2D, 2E. 4A, 4B. Further, full length images of Western blots (not cropped) either in supplement or supporting figure resource is highly recommended.

***Authors’ response:*** *Thanks for your suggestions. We have added the molecular weights positions based on markers in all western blot figures, and also all full-length images of western blots have been provided in supporting figure resource.*

**15. Comments:**

Please include further detail about filtering of RIME data. Bioinformatic analysis steps are included for -seq based analysis. What cutoffs were used to determine the final inclusion/exclusion list of 177 interacting proteins. Was PRDM9 identified?

***Authors’ response:*** *Thanks for your comments. Finally, 177 ZCWPW1-interacting proteins were identified in three replicates. However, PRDM9 was not detected among the 177 ZCWPW1 interacting proteins. We have now added the bioinformatic analysis of the RIME-seq data in our revised manuscripts as follows:*

*“RIME analysis was performed according to published protocols(Mohammed et al., 2016) using ZCWPW1 and IgG antibodies. Briefly, The RIME raw data were processed using Proteome Discoverer (v1.4) and Mascot and/or SEQUEST as search engines. The identified proteins were filtered according to their false discovery rate and spectral count. Only the proteins with -10lgP≥20 and at least 1 unique peptide were retained. Moreover, to improve the confidence of the identified proteins, only proteins with at least 5 spectral counts were considered as positive. To exclude non-specificity, the proteins identified in the ZCWPW1 antibody group should not be identified in the negative IgG controls in the same batch experiments.”*

**16. Comments:**

Figure 4D. What is the n of this experiment? Please show individual data points on bar graphs.

***Authors’ response:*** *We have performed the neutral comet assay six times, and the individual data points have been added on bar graphs.*

**17. Comments:**

Please include reference (Imai et al. Elife. Oct. 2020) for HELLS in the introduction

***Authors’ response:*** *Thanks, this has been fixed.*

*“In mice, the chromatin remodeler HELLS, which is recruited to the recombination hotspots by PRDM9, can open chromatin at future DSB hotspots and is required for DSB activity at PRDM9 sites(Imai et al., 2020; Spruce et al., 2020).”*

**Reviewer #3:** 022 ZCWPW1 paper

Overview:

Meiotic recombination in mammals is modulated by the histone methylase PRDM9 and the reader ZCWPW1, but precisely how these factors work mechanistically, is unclear. Here the authors report genome-wide analysis of chromatin marks (ChIP-seq) and chromatin accessibility (using ATAC-seq) in meiotic cells comparing controls to meiocytes lacking PRDM9, ZCWPW1 and SPO11 expression. Overall, the findings are quite interesting if a little provisional. The major observation of interest is that H3K9Ac mark is dependent on both the writer (PRDM9) and the reader (ZCWPW1) activity.

A number of issues are outstanding. For example, it is unclear ambiguous which datasets are new and which are reanalysis of published work. The authors would do well to make this much clearer in any revised manuscript. Secondly, the description of data analysis and its interpretation often lacks clarity, and may be often be over stated—but I am sure this can be addressed. Finally, the ectopic expression of ZCWPW1 in HeLa cells is potentially interesting, but appears to lack important controls concerning cell cycle distribution (again, something that should be quite easy to determine, but may alter the author's interpretations of their observations).

***Authors’ response:*** *We thank the reviewer for his/her positive assessment of our work. Firstly, we apologize for the confusion. To make it clear, we have summarized all datasets used in this study in the revised manuscript in supplementary table 2.*

*Also, we appreciate these very constructive suggestions from the reviewer. In the revised version, we have performed new experiments and analyses following the reviewer’s requests and suggestions.*

Specific comments:

**1. Comments:**

Fig 1B. Please indicate in these and any other heatmaps by which parameter the data are ordered vertically.

***Authors’ response:*** *We used Deeptools to plot the heatmaps (see Methods). In Fig 1B, S1B, S2A, and S2B, the regions in heatmaps were ordered from largest to smallest based on the average signal in all samples (default parameters). In Fig 4B and Fig S5D, the regions were ordered from largest to smallest based on the average signal of ZCWPW1 ChIP-seq.*

**2. Comments:**

Line 131: For comparison, what is the expected (random) overlap of ZCWPW1 and H3K9Ac in a null hypothesis that there is no relationship?

***Authors’ response:*** *We have generated the random regions of ZCWPW1 peaks in the mouse genome, and then calculated the overlap regions with H3K9ac. We find there are only 532 random regions that overlap with H3K9ac, which is strikingly reduced compared with the observed results (Fig. R5).*

*Figure R5. The number of ZCWPW1 peaks overlapping with H3K9ac.*

**3. Comments:**

Line 139 and 142. Unless I am mistaken it is important to make it clear that Group 1 and Group 2 are DEFINED by their loss/retention of H3K9Ac in the absence of ZCWPW1. This is currently not clear.

***Authors’ response:*** *We thank the reviewer for this suggestion. As directed, we have now included all such information in the revised manuscript and figure legends.*

*Line 149 and 154. “Interestingly, 74% (4886/6627) (Group H1) of the ZCWPW1 binding sites lost H3K9ac signal in Zcwpw1^−/−^ mouse testes (Fig. 1A, 1B Group H1 and S2C), suggesting that ZCWPW1 somehow functionally impacts the H3K9ac at ZCWPW1 binding sites. However, 26% (1741/6627) (Group H2) of the ZCWPW1 binding sites still retained H3K9ac signal in Zcwpw1^−/−^ mouse testes (Fig. 1B Group H2 and S2C).”*

*Line 431 and 433. “Group H1 indicated ZCWPW1 binding sites that lost the H3K9ac signal in Zcwpw1^−/−^ testes, while Group H2 indicated ZCWPW1 binding sites that retained the H3K9ac signal in Zcwpw1^−/−^ testes.”*

*Line 517 and 519. “Group H1 indicated ZCWPW1 binding sites that lost the H3K9ac signal in Zcwpw1^−/−^ testes, while Group H2 indicated ZCWPW1 binding sites that retained the H3K9ac signal in Zcwpw1^−/−^ testes.”*

**4. Comments:**

Line 144. The term "significantly" is used, yet I don't read any use of statistical tests. Either add a statistical test or revise the text to avoid use of significant, and state, "greatly", or some other adjective that describes the data.

***Authors’ response:*** *We have comprehensively reworked the text, now using "greatly" to replace "significantly" where there are no statistical tests.*

**5. Comments:**

Section 158-167. Is there a negative control for the Mass Spec analysis? The three replicates appear very different, with only minimal overlap. Are the HDACs known non-specific contaminants? It would help if these concerns were addressed in the text.

***Authors’ response:*** *Detailed information of RIME assay and RIME data analysis have been revised in the manuscript. Briefly, we have performed three independent experiments, and the IgG antibody was used as negative control for the Mass spec analysis. The minimal overlap of three replicates might be due to the batch effect. There are no HDACs in IgG control groups. To verify the candidate molecules identified in ZCWPW1 RIME assay, we have confirmed the interaction between ZCWPW1 and several candidates both in testes and in HeLa cells and cell lines by Co-IP, which was further confirmed by yeast two-hybrids. We have now added the bioinformatic analysis of the RIME-seq data in our revised manuscripts as follows:*

*“RIME analysis was performed according to published protocols(Mohammed et al., 2016) using ZCWPW1 and IgG antibodies. Briefly, The RIME raw data were processed using Proteome Discoverer (v1.4) and Mascot and/or SEQUEST as search engines. The identified proteins were filtered according to their false discovery rate and spectral count. Only the proteins with -10lgP≥20 and at least 1 unique peptide were retained. Moreover, to improve the confidence of the identified proteins, only proteins with at least 5 spectral counts were considered as positive. To exclude non-specificity, the proteins identified in the ZCWPW1 antibody group should not be identified in the negative IgG controls in the same batch experiments.”*

**6. Comments:**

Line 183, 186. Again, "significant" is used. By what measure? Was this quantitative and reproducible? How large is the effect? Please avoid the potential for overstating the observations. My reading of the data presented is that the effect on H3K9Ac of overexpressing ZCWPW1 is, at best, modest.

***Authors’ response:*** *We appreciate this directed emphasis on quantitative precision.*

*And now replication and quantification have been provided in Fig. 3A, 3B and 3C. In the re-drafted text, this content now reads as follows:*

*“Given that knock-out of Zcwpw1 could greatly reduce the H3K9ac signal at recombination hotspots (Fig. 1B) but did not affect the protein levels of the HDACs that ZCWPW1 interacts with (Fig. S4A), we hypothesized that ZCWPW1 antagonizes the deacetylation activity of HDACs to maintain the H3K9ac signal. To test this, we expressed ZCWPW1 in HeLa cells. The H3K9ac signal was significantly increased in HeLa cells expressing ZCWPW1 compared to control cells transfected with an empty-vector (Fig. 3A, 3B and 3C). Further, we expressed HDAC6 alone or with ZCWPW1 in HeLa cells, and the H3K9ac signal was slightly reduced in the HDAC6 overexpression group but was partly recovered in the ZCWPW1 and HDAC6 co-expression group (Fig. 3B). We also performed HDAC activity assays in HeLa cells, and as expected the deacetylation activities of HDACs were modestly decreased in cells expressing ZCWPW1 compared to controls (Fig. 3D). Thus, ZCWPW1’s interaction with HDAC enzymes restricts their deacetylation activities, at least partially explaining the elevated H3K9ac levels at recombination hotspots.”*

**7. Comments:**

Is there a reference for the assay used in Fig 1F? If not, please describe in more detail.

***Authors’ response:*** *To make it clear, we have described this assay in more detail and also added the reference paper information in the revised method.*

*“The HDAC activity assay was performed using the Histone Deacetylase Activity Assay Kit (ABCAM, #ab156064) according to the manufacturer’s instructions(Lee et al., 2013). The HDAC activity assay is based on an AMC-conjugated acetylated peptide. AMC is a fluorescent dye whose fluorescence is quenched when conjugated to the acetylated peptide. Once the HDACs de-acetylate the acetylated peptide, it becomes susceptible to cleavage by an enzyme (the Developer component), which results in releasing free AMC, the fluorescence of which can be measured by a fluorescence microplate reader (Ex/Em 355/460 nm). Briefly, HeLa cells were cultured in a 6-well plate transfected with indicated plasmids using X-tremeGENE HP DNA Transfection Reagent following the manufacturer’s protocol. After 24 h, 10 μl nuclear extracts from transfected HeLa cells were mixed with HDAC assay buffer, substrate, and developer and then incubated at room temperature for 2 h. Fluorescence intensity was measured with a Perkin Elmer EnVision (Excitation 355 nm, Emission 460 nm).”*

**8. Comments:**

Line 198: Where are the data that show the number of open sites out of the total sites analyzed? Please describe clearly. What were the criteria for a site to be considered "open"?

***Authors’ response:*** *In the re-drafted text, this content now reads as follows:*

***“****Here, in the ATAC-seq data from the wild type mouse testes, we detected that 4015 of 14688 ZCWPW binding sites were open.”*

*If the ZCWPW1 binding sites overlap with the ATAC peaks, these sites will be considered “open”.*

**9. Comments:**

Line 199: The text is ambiguous. Is it 67% of group 1 sites? Or 67% of all sites? Are these group 1 and group 2 sites the same as those defined in Fig 1? If not, please explain how they were defined - by what criteria/threshold etc.

***Authors’ response:*** *We apologize for the confusion. We have detected 6627 H3K9ac peaks and 4015 ATAC signals overlapping ZCWPW1 peaks in whole testes. Here to make it clearer, we rename group H1 and group H2 in figure1 and group A1 and group A2 in figure 3. Group H1 represents those ZCWPW1 binding sites lost H3K9ac peaks in Zcwpw1^−/−^ testes; Group H2 represents those ZCWPW1 binding sites retained H3K9ac peaks in Zcwpw1^−/−^ testes. Group A1 indicates ZCWPW1 binding sites with dramatic loss of ATAC signal in Zcwpw1^−/−^ testes, while Group A2 indicates ZCWPW1 binding sites that retained ATAC signal in Zcwpw1^−/−^ testes.*

*In the re-drafted text, this content now reads as follows:*

*“Next, we compared the ATAC signal in WT and Zcwpw1^−/−^ testes. We found that 67% (2676/4015) (Group A1) of the ZCWPW1 binding sites lost the ATAC signal in Zcwpw1^−/−^ mouse testes (Fig. 4A and 4B), indicating that ZCWPW1 regulates chromatin openness at ZCWPW1 binding sites, while 33% (1339/4015) (Group A2) of the ZCWPW1 binding sites retained the ATAC signal in Zcwpw1^−/−^ mouse testes (Fig. 4B).”*

**10. Comments:**

As above, please specify quantitative criteria that is used to decide "open-ness". Is this an arbitrary cut off? Please explain. The data look convincing, but the data description is lacking in clarity.

***Authors’ response:*** *If the ZCWPW1 binding sites overlap the ATAC peaks, these sites will be considered “open”. Detailed information defining "open-ness" has been provided in method. Kindly see our revised method “ATAC-seq bioinformatics analysis”.*

**11. Comments:**

Line 202: Same issues as the points above. Please clarify.

***Authors’ response:*** *We apologize for the confusion. This has been fixed. Kindly see the response for essential comment 9.*

**12. Comments:**

Line 213. Which ZCWPW1 binding sites does "group 1" refer to? Those in Fig 1 (4886 sites) or in Fig 3 (2676 sites)? It appears that these are different definitions, but that is not explained and appears confusing (to this reviewer…).

***Authors’ response:*** *We apologize for the confusion. We have detected 6627 H3K9ac peaks and 4015 ATAC signals overlapping ZCWPW1 peaks in whole testes. Here to make it clear, we rename group H1 and group H2 in figure1 and group A1 and group A2 in figure3. Group H1 represents those ZCWPW1 binding sites that lost H3K9ac peaks in Zcwpw1^−/−^ testes; Group H2 represents those ZCWPW1 binding sites that retained H3K9ac peaks in Zcwpw1^−/−^ testes. Group A1 indicates ZCWPW1 binding sites with dramatic loss of ATAC signal in Zcwpw1^-/-^ testes, while Group A2 indicates ZCWPW1 binding sites that retained ATAC signal in Zcwpw1^−/−^ testes. We now use new definitional group names in all related sections of the revised text.*

*Line 213. "Group 1" ZCWPW1 binding sites refer to those in Fig 3(2676 sites), now renamed as group A1.*

**13. Comments:**

CRITICAL: Line 226. Please specify which agents are being. Fig S5 only shows Hydroxyurea.

***Authors’ response:*** *In the re-drafted text, this content now reads as follows:*

*“We also expressed ZCWPW1 in HeLa cells and then treated them with Hydroxyurea to induce DSBs, and the cells expressing ZCWPW1 showed reduced fluorescence intensity for the DSB damage marker γH2AX (Fig. S7A and S7B).”*

**14. Comments:**

CRITICAL: Given that HU and CPT are replication inhibitor/poison (and etoposide is also important for cycling cells): Did ZCWPW1 expression affect the proportion of cells in S-phase (independently of HU treatment)? Or the length of the cell cycle/doubling time? This is an important control/test to report for this entire section of work (Fig 4).

***Authors’ response:*** *We thank the reviewer for raising this very important question. In the revised version, we have added such information as follows:*

*“Given the observed impacts of ZCWPW1 in regulating histone acetylation and chromatin accessibility, we tested whether ectopic expression of the germ-cell-specific protein ZCWPW1 in human cells affects DSB repair. We first expressed ZCWPW1 in HeLa cells and found that the length of the cell doubling time was comparable between the control and ZCWPW1 overexpression groups (Fig. S6A and S6B). We also performed a cell cycle assay using flow cytometry in control and ZCWPW1-overexpressing HeLa cells, and the proportion of cells in S-phase was comparable between the two groups (Fig. S6C and S6D).”*

***References:***

*Adelman, C.A., and Petrini, J.H. (2008). ZIP4H (TEX11) deficiency in the mouse impairs meiotic double strand break repair and the regulation of crossing over. PLoS Genet 4, e1000042.*

*Baudat, F., Imai, Y., and de Massy, B. (2013). Meiotic recombination in mammals: localization and regulation. Nat Rev Genet 14, 794-806.*

*Bayley, R., Borel, V., Moss, R.J., Sweatman, E., Ruis, P., Ormrod, A., Goula, A., Mottram, R.M.A., Stanage, T., Hewitt, G., et al. (2022). H3K4 methylation by SETD1A/BOD1L facilitates RIF1-dependent NHEJ. Mol Cell 82, 1924-1939 e1910.*

*Bellve, A.R., Cavicchia, J.C., Millette, C.F., O'Brien, D.A., Bhatnagar, Y.M., and Dym, M. (1977). Spermatogenic cells of the prepuberal mouse. Isolation and morphological characterization. J Cell Biol 74, 68-85.*

*Buard, J., Barthes, P., Grey, C., and de Massy, B. (2009). Distinct histone modifications define initiation and repair of meiotic recombination in the mouse. EMBO J 28, 2616-2624.*

*Chen, Y., Lyu, R., Rong, B., Zheng, Y., Lin, Z., Dai, R., Zhang, X., Xie, N., Wang, S., Tang, F., et al. (2020). Refined spatial temporal epigenomic profiling reveals intrinsic connection between PRDM9-mediated H3K4me3 and the fate of double-stranded breaks. Cell Res 30, 256-268.*

*Diagouraga, B., Clement, J.A.J., Duret, L., Kadlec, J., de Massy, B., and Baudat, F. (2018). PRDM9 Methyltransferase Activity Is Essential for Meiotic DNA Double-Strand Break Formation at Its Binding Sites. Mol Cell 69, 853-865 e856.*

*Dix, D.J., Allen, J.W., Collins, B.W., Mori, C., Nakamura, N., Poorman-Allen, P., Goulding, E.H., and Eddy, E.M. (1996). Targeted gene disruption of Hsp70-2 results in failed meiosis, germ cell apoptosis, and male infertility. Proc Natl Acad Sci U S A 93, 3264-3268.*

*Grey, C., Clement, J.A., Buard, J., Leblanc, B., Gut, I., Gut, M., Duret, L., and de Massy, B. (2017). In vivo binding of PRDM9 reveals interactions with noncanonical genomic sites. Genome Res 27, 580-590.*

*Herran, Y., Gutierrez-Caballero, C., Sanchez-Martin, M., Hernandez, T., Viera, A., Barbero, J.L., de Alava, E., de Rooij, D.G., Suja, J.A., Llano, E., et al. (2011). The cohesin subunit RAD21L functions in meiotic synapsis and exhibits sexual dimorphism in fertility. EMBO J 30, 3091-3105.*

*Huang, T., Yuan, S., Gao, L., Li, M., Yu, X., Zhan, J., Yin, Y., Liu, C., Zhang, C., Lu, G., et al. (2020). The histone modification reader ZCWPW1 links histone methylation to PRDM9-induced double-strand break repair. Elife 9.*

*Imai, Y., Biot, M., Clement, J.A., Teragaki, M., Urbach, S., Robert, T., Baudat, F., Grey, C., and de Massy, B. (2020). PRDM9 activity depends on HELLS and promotes local 5-hydroxymethylcytosine enrichment. Elife 9.*

*Khil, P.P., Smagulova, F., Brick, K.M., Camerini-Otero, R.D., and Petukhova, G.V. (2012). Sensitive mapping of recombination hotspots using sequencing-based detection of ssDNA. Genome Res 22, 957-965.*

*Kumar, R., Oliver, C., Brun, C., Juarez-Martinez, A.B., Tarabay, Y., Kadlec, J., and de Massy, B. (2018). Mouse REC114 is essential for meiotic DNA double-strand break formation and forms a complex with MEI4. Life Sci Alliance 1, e201800259.*

*Lam, K.G., Brick, K., Cheng, G., Pratto, F., and Camerini-Otero, R.D. (2019). Cell-type-specific genomics reveals histone modification dynamics in mammalian meiosis. Nat Commun 10, 3821.*

*Lee, S.J., Lindsey, S., Graves, B., Yoo, S., Olson, J.M., and Langhans, S.A. (2013). Sonic hedgehog-induced histone deacetylase activation is required for cerebellar granule precursor hyperplasia in medulloblastoma. PLoS One 8, e71455.*

*Li, M., Huang, T., Li, M.J., Zhang, C.X., Yu, X.C., Yin, Y.Y., Liu, C., Wang, X., Feng, H.W., Zhang, T., et al. (2019). The histone modification reader ZCWPW1 is required for meiosis prophase I in male but not in female mice. Sci Adv 5, eaax1101.*

*Mahgoub, M., Paiano, J., Bruno, M., Wu, W., Pathuri, S., Zhang, X., Ralls, S., Cheng, X., Nussenzweig, A., and Macfarlan, T.S. (2020). Dual histone methyl reader ZCWPW1 facilitates repair of meiotic double strand breaks in male mice. Elife 9.*

*Manickavinayaham, S., Velez-Cruz, R., Biswas, A.K., Bedford, E., Klein, B.J., Kutateladze, T.G., Liu, B., Bedford, M.T., and Johnson, D.G. (2019). E2F1 acetylation directs p300/CBP-mediated histone acetylation at DNA double-strand breaks to facilitate repair. Nat Commun 10, 4951.*

*Mohammed, H., Taylor, C., Brown, G.D., Papachristou, E.K., Carroll, J.S., and D'Santos, C.S. (2016). Rapid immunoprecipitation mass spectrometry of endogenous proteins (RIME) for analysis of chromatin complexes. Nat Protoc 11, 316-326.*

*Ogiwara, H., Ui, A., Otsuka, A., Satoh, H., Yokomi, I., Nakajima, S., Yasui, A., Yokota, J., and Kohno, T. (2011). Histone acetylation by CBP and p300 at double-strand break sites facilitates SWI/SNF chromatin remodeling and the recruitment of non-homologous end joining factors. Oncogene 30, 2135-2146.*

*Panizza, S., Mendoza, M.A., Berlinger, M., Huang, L., Nicolas, A., Shirahige, K., and Klein, F. (2011). Spo11-accessory proteins link double-strand break sites to the chromosome axis in early meiotic recombination. Cell 146, 372-383.*

*Pfister, S.X., Ahrabi, S., Zalmas, L.P., Sarkar, S., Aymard, F., Bachrati, C.Z., Helleday, T., Legube, G., La Thangue, N.B., Porter, A.C., et al. (2014). SETD2-dependent histone H3K36 trimethylation is required for homologous recombination repair and genome stability. Cell Rep 7, 2006-2018.*

*Powers, N.R., Parvanov, E.D., Baker, C.L., Walker, M., Petkov, P.M., and Paigen, K. (2016). The Meiotic Recombination Activator PRDM9 Trimethylates Both H3K36 and H3K4 at Recombination Hotspots In Vivo. PLoS Genet 12, e1006146.*

*Price, B.D., and D'Andrea, A.D. (2013). Chromatin remodeling at DNA double-strand breaks. Cell 152, 1344-1354.*

*Spruce, C., Dlamini, S., Ananda, G., Bronkema, N., Tian, H., Paigen, K., Carter, G.W., and Baker, C.L. (2020). HELLS and PRDM9 form a pioneer complex to open chromatin at meiotic recombination hot spots. Genes Dev 34, 398-412.*

*Wagner, F.F., Zhang, Y.L., Fass, D.M., Joseph, N., Gale, J.P., Weiwer, M., McCarren, P., Fisher, S.L., Kaya, T., Zhao, W.N., et al. (2015). Kinetically Selective Inhibitors of Histone Deacetylase 2 (HDAC2) as Cognition Enhancers. Chem Sci 6, 804-815.*

*Wang, Z.A., Millard, C.J., Lin, C.L., Gurnett, J.E., Wu, M., Lee, K., Fairall, L., Schwabe, J.W., and Cole, P.A. (2020). Diverse nucleosome Site-Selectivity among histone deacetylase complexes. Elife 9.*

*Wells, D., Bitoun, E., Moralli, D., Zhang, G., Hinch, A., Jankowska, J., Donnelly, P., Green, C., and Myers, S.R. (2020). ZCWPW1 is recruited to recombination hotspots by PRDM9 and is essential for meiotic double strand break repair. Elife 9.*

*Yuan, L., Liu, J.G., Zhao, J., Brundell, E., Daneholt, B., and Hoog, C. (2000). The murine SCP3 gene is required for synaptonemal complex assembly, chromosome synapsis, and male fertility. Mol Cell 5, 73-83.*

**Second round of review**

**Reviewer 1**

The revised manuscript has addressed most of the points raised earlier in the reviews.

One outstanding point is that the formal demonstration that H3K4me3 is still at DSB hotspots in the absence of ZCWPW1 ( (reviewer figure 4) should be included in the manuscript. It is curious that this hasn’t been shown before, and it strengthens the author's points.

**Reviewer 2**

Yuan et al. have resubmitted a greatly revised manuscript on the topic of ZCWPW1-mediated stabilization of H3K9ac and chromatin accessibility at meiotic recombination hotspots.

The authors provided useful comments and/or corrections to most of the reviewer's concerns. In some cases, the authors have performed additional experiments to support their manuscript and overall model of ZCWPW1 function.

Generally, I am satisfied with the editorial changes to the manuscript. However, in a few cases while the authors did provide additional experiments based on reviewers concerns in their reply to reviewers, these experiments did not get incorporated into the revised manuscript. It is not entirely clear why these were provided to the reviewers but not in the manuscript, even if in the supplementary data. Specifically, I am referring to 2 experiments which are the co-IP experiment performed in WT and Zcwpw1 null mice (what is labeled as Figure R2) and the H3K4me3 ChIP-seq experiment in ZCWPW1 null mice (what is labeled as Figure R4). The first experiment shows the level of 'background' or non-specific signal in the coIPs with HDACs as bands are still present at the expected molecular weights in the knock-out strain. This will give readers some indication of the specificity of these experiments. The second experiment (ChIP-seq) provides further support of the authors model that loss of ZCWPW1 leads to loss of H3K9ac and chromatin accessibility specifically, and not loss of PRDM9-dependent modifications. This supports the idea that ZCWPW1 function is downstream from PRDM9 as proposed in their model. I strongly feel that both of these results should be made available in the manuscript.

**Reviewer 3**

ZCWPW1 second review.

I thank the authors for taking the time to respond to my comments. I have only a couple of minor follow-up comments that I think are still important to address in order to help clarify the manuscript and make it more accessible/understandable for their future readers.

Below I refer to the "Comment" heading numbers that the authors used in their rebuttal document.

1. Comment 1: The authors have answered the question I posed (in their rebuttal), but have not updated the relevant figure legends to provide this information for their readers. Thus I feel that this remains an issue that they need to resolve in a further (minor) update to the text.

2. Comment 2: As above, please specify where this information (from the rebuttal) has been added to the manuscript. (Inclusion of a description of the analysis as a statement in the text, rather than needing to add the figure, would be fine.)

3. Comment 8-11: It would be helpful (to their readers) if the main text (not just the methods) contained a brief explanation of what criteria the authors use to define "open". i.e. 25% overlap with ATAC-seq peaks.
